# Women's Causal Beliefs About Their Pelvic Pain: A Systematic Review and Meta‐Ethnography

**DOI:** 10.1111/papr.70194

**Published:** 2026-08-28

**Authors:** Grace A. Ferencz, Amelia K. Mardon, Alice M. Mitchell, Saravana Kumar, K. Jane Chalmers

**Affiliations:** ^1^ IIMPACT in Health, School of Allied Health and Human Performance, College of Health Adelaide University Adelaide South Australia Australia; ^2^ NICM Health Research Institute Western Sydney University Penrith New South Wales Australia; ^3^ Department of Obstetrics, Gynaecology and Newborn Health University of Melbourne Melbourne Victoria Australia; ^4^ School of Health Sciences Western Sydney University Penrith New South Wales Australia

**Keywords:** meta‐ethnography, pain beliefs, pelvic pain, qualitative, systematic review

## Abstract

**Introduction:**

This study identified and synthesized the current qualitative literature on women's causal beliefs about their chronic pelvic pain.

**Methods:**

Academic databases and relevant gray literature sources were searched from their inception to August 2025 with an English language limiter. Study quality was assessed using the Critical Appraisal Skills Programme (CASP) qualitative checklist. A meta‐ethnography was used to synthesize findings.

**Results:**

Thirty studies including 1255 women from a range of countries were deemed eligible for inclusion. Seven studies fully met the CASP criteria and were deemed to have minimal risk of bias. The quality of reporting and demographic diversity of included studies were limited, often lacking consideration of researcher‐participant relationships, and primarily including Caucasian, cis‐gender, heterosexual women from Western countries. Four themes were generated: (1) *It's happening to me because I'm a woman*, (2) *It's happening to me in my body*, (3) *It's happening to me due to my actions*, and (4) *It's happening to me due to my life*. Women's pain attributions varied from siloed biological, psychological, or social explanations to combinations of these factors. Their perceptions of control also ranged from feeling entirely powerless to experiencing a sense of personal agency. A conceptual model was developed to represent the range of beliefs women hold about the cause of their pelvic pain.

**Conclusion:**

Understanding women's causal beliefs about their chronic pelvic pain may inform clinical assessment to provide a foundation for recovery. This study was unfunded and was preregistered on Open Science Framework (https://osf.io/wkc5v/) and PROSPERO (CRD42023444018).

## 1 Introduction

Chronic pelvic pain is a highly prevalent and burdensome condition which disproportionately affects between 6.4% and 25.4% of women [1, 2, 3]. Chronic pelvic pain, herein referred to as pelvic pain, can be defined as pain in the pelvic region which lasts for more than 6 months and results in functional disability or requires medical intervention [4]. Pelvic pain creates billions of dollars of economic burden each year [5] which incorporates diagnostics, treatment, and surgical costs [6]. Pelvic pain can impact all aspects of a woman's life [6, 7, 8, 9], including school [10] and workplace absenteeism [11], lost productivity [6], and termination of employment [7, 12]. Many women with pelvic pain report high levels of depression and anxiety [6, 7, 13], and greater sexual dissatisfaction and difficulty than women without pelvic pain [14, 15]. This broad, multifactorial presentation emphasizes the complex biopsychosocial nature of pelvic pain [14, 16]. Such biopsychosocial contexts are unique to the individual and are heavily intertwined and influenced by an individual's beliefs, which may shape their experiences and outcomes [17]. The biopsychosocial approach to healthcare integrates biological causes (including physiological disease), psychological elements (such as emotions and cognition), and social and structural aspects (including cultural and familial influences), to provide a holistic and person‐centred approach to contemporary healthcare [18]. The biopsychosocial approach to healthcare expands on the narrower biomedical approach (focused on managing illness at a purely biological level), by incorporating a broader viewpoint taking into account an individual's unique health context [18].

Beliefs about pain and the body have the power to influence emotions and behaviors relating to chronic pain. Beliefs can be defined as something one accepts as true or real [19] and, in this study, causal beliefs refer to what individuals believe causes their pain [20]. Individuals who believe that their pain is due to a pathology are more likely to have a higher pain intensity and reduced functional ability [17]. Understanding individuals' causal beliefs provides insight into their knowledge and understanding of pain mechanisms and may help explain their current attitudes and behaviors towards pain management [20, 21]. Beliefs can be modified through education and experiential learning [22, 23]. Addressing women's causal beliefs about their pelvic pain through education has been shown to improve pain knowledge and understanding, which may enhance pain rehabilitation [22, 24]. Understanding the causal beliefs women hold about their pelvic pain may therefore provide scope for improving clinical outcomes.

Previous studies have shown that women with pelvic pain hold misconceptions about the cause of their pain and these misconceptions can influence pain outcomes [25, 26]. For instance, in a previous study it was found that beliefs held by women with pelvic pain about the cause of their pain and the physical severity of their condition influenced their pain experience irrespective of their laparoscopy results [27]. Regardless of the presence or absence of physiological disease found during laparoscopy, women who believed their pain had a physical cause and rated their condition as more severe experienced a greater degree of pain [27]. If causal beliefs can impact pelvic pain experienced by women, then gaining a deep understanding of these beliefs is an important step towards improving pain outcomes. While some studies have investigated the causal pain beliefs held by women with pelvic pain, synthesis of these studies has not been performed. Therefore, the aim of this study was to identify, critically appraise, and synthesize the current literature on women's causal beliefs about their pelvic pain.

## 2 Materials and Methods

This review was designed in accordance with recommendations from Sattar et al. [28] and reported in alignment with the Preferred Reporting Items for Systematic Review and Meta‐Analyses checklist (PRISMA) [29] and eMERGE meta‐ethnography reporting guidance [30]. The study protocol was pre‐registered on Open Science Framework (https://osf.io/wkc5v/) and PROSPERO (CRD42023444018) [31] on July 10, 2023. All deviations are noted within this manuscript and presented in the Open Science Framework preregistered protocol.

### 2.1 Eligibility Criteria

A Sample, Phenomenon of Interest, Design, Evaluation, Research type (SPIDER) framework [32] was used to define the research aim and strategy. Qualitative research exploring the causal beliefs held by women about their pelvic pain was included in this review. Included studies were those that: (1) recruited women of any age diagnosed with pelvic pain; (2) explored the causal beliefs held by participants regarding their pelvic pain; (3) were peer‐reviewed academic or from relevant gray literature sources; and (4) were published in English. Excluded studies were those that: (1) included conditions associated with the gastrointestinal tract, pelvic infections, inflammatory disease, or pregnancy; (2) only included secondary data; or (3) only contained quantitative data. Where a study contained data from both women and men, the study was assessed as to whether the data from women could be extracted separately from the data from men. If this was possible, only then was the study included. For the purpose of this research, the terms woman/women are used to describe individuals whose sex presumed and assigned at birth (AFAB) was female. These terms should be taken to include people who have a gender identity that means they do not identify as women but who are AFAB [33].

### 2.2 Literature Search

The initial search was conducted from inception until July 2023 and updated in August 2025. Six databases were searched including: MEDLINE, EMBASE, EmCare, PsycINFO, Scopus, and Web of Science Core Collection. The Trove Australian online database aggregator and service, and ProQuest Dissertations and Theses Global were searched for degree theses and dissertations. Additional searches were performed in Google and Google Scholar (first 50 references) and through screening the reference lists of included studies and relevant organization's websites (e.g., Endometriosis Australia and the Pelvic Pain Foundation of Australia). Where conference abstracts were identified, we attempted to locate the full text publication. Where the full text publication could not be retrieved, the study was excluded. A comprehensive search strategy was developed by the research team in collaboration with an academic librarian (File S1). Medical Subject Headings and key words associated with ‘chronic pelvic pain’ and ‘beliefs’ were combined with the Boolean operator AND for the search. No time limits were applied, though English language limits were utilized.

### 2.3 Literature Selection

Studies identified by the search were exported to EndNote (version 20.5, Clarivate, Philadelphia, PA, USA) and uploaded to Covidence (Veritas Health Innovation, Melbourne, Victoria, Australia) where duplicates were removed. Title and abstract screening, and full‐text screening were completed using Covidence. Two reviewers (GAF and AKM/AMM/SK/KJC) independently screened the title and abstract of each study against the inclusion and exclusion criteria. Two reviewers (GAF and KJC) then independently screened the full text of remaining studies for inclusion, with reasons for exclusion recorded. Disagreements were resolved through discussion. The updated search followed the same methods as the original, with the exception that studies were only included if they explicitly aimed to explore participants' beliefs about their pelvic pain.

### 2.4 Data Extraction

Two reviewers (GAF and AKM/AMM/KJC) independently extracted data from the eligible studies using a customized and pilot tested Excel spreadsheet (version 16.0, Microsoft Corporation, Redmond, WA, USA). Disagreements were resolved through discussion or by consultation with a third reviewer (AKM/AMM/KJC). Extracted data included publication details, study characteristics (e.g., study design, study location), participant characteristics (e.g., sample size, age), as well as first order data (participant quotes) and second order data (researchers' interpretations of their findings).

### 2.5 Quality and Reporting Appraisal

The Critical Appraisal Skills Programme (CASP) [34] qualitative checklist was used to assess the methodological quality of the included studies. The CASP [34] checklist is commonly used in health‐related qualitative reviews [35] and is endorsed by the Cochrane Qualitative and Implementation Methods Group for use in qualitative studies [36]. Studies are scored out of ten, where a higher rating indicates higher methodological quality [28]. Critical appraisal was independently conducted by two reviewers (GAF and AKM/AMM/KJC), with disagreements resolved through discussion or by consultation with a third reviewer (AKM/AMM/SK/KJC). Additionally, the COnsolidated criteria for REporting Qualitative research (COREQ) [37] checklist was used to evaluate the reporting of included studies and identify any reporting bias. While the COREQ is intended as a reporting guidance, currently no tool exists to identify reporting bias; therefore, the COREQ was used to evaluate consistency of reporting.

### 2.6 Data Synthesis

A meta‐synthesis was performed using a meta‐ethnographic approach. Meta‐ethnography was chosen to allow for higher order interpretation and conceptualisation of women's causal beliefs about their pelvic pain across a range of study approaches, with the intention of providing evidence to inform healthcare policies and practices [28]. In accordance with recommendations by Sattar et al. [28], the following seven stages were performed for the synthesis process: (1) Three reviewers (GAF, AKM, AMM, and KJC) read studies repeatedly to become familiar with their key concepts and content. (2) Two reviewers (GAF and AKM/AMM/KJC) extracted first and second order constructs from the data, consisting of participants' quotes and researchers' interpretations. Data were extracted verbatim and original terminology preserved. (3) The primary researcher (GAF) then grouped similar findings together to establish relationships between key concepts of the different studies. Coding was moderated through member checking and peer debriefing with the research team. (4) The primary researcher then inductively organized similar concepts into relevant categories according to common underlying meaning. Categories were reviewed and revised through discussion with the research team to ensure they encompassed all relevant concepts. (5) The primary researcher then compared each concept extracted from each study with all other studies to identify commonality or opposition. This highlighted the similarities and differences between the concepts and interpretations, and this process was reviewed and revised by the wider research team. (6) The primary researcher then organized concepts into conceptual categories, which resulted in the development of higher third order constructs (themes). Thematic mapping [38] was used to explore similarities and differences and to clarify boundaries between themes, which were iteratively refined to differentiate overlapping concepts. This process was moderated through discussion with the wider research team. (7) The primary researcher then performed a line of argument through interpretation of the relationships between the themes to provide scope for developing new insights. The primary researcher maintained a reflexive journal throughout data analysis to document the iterative coding process, recording key decisions, influences, and reflections as they occurred. The research team reviewed, refined, and collapsed codes as necessary, with revision of codes and themes occurring fortnightly over 6 months to ensure the reliability of theme development. Any disagreements throughout the process were resolved through consultation and consensus with the research team. Confidence in the review findings was assessed using the GRADE‐CERQual approach [39].

### 2.7 Positionality Statement

The research team acknowledges that their own experiences may influence their interpretations and analysis of the data. G.A.F. is a female research honors and physiotherapy student as well as a physiotherapy assistant. K.J.C. is a female senior lecturer, researcher, and physiotherapist with extensive professional experience in gynecological health and chronic pain research. A.K.M. is a female postdoctoral researcher with experience in gynecological health and chronic pain research and has lived experience of pelvic pain. A.M.M. is a female PhD candidate and physiotherapist with experience in gynecological health and chronic pain research. S.K. is a male professor in allied health and health services research, physiotherapist, and lecturer with extensive professional experience in health services research and implementation science.

## 3 Results

### 3.1 Literature Selection

**FIGURE 1:**
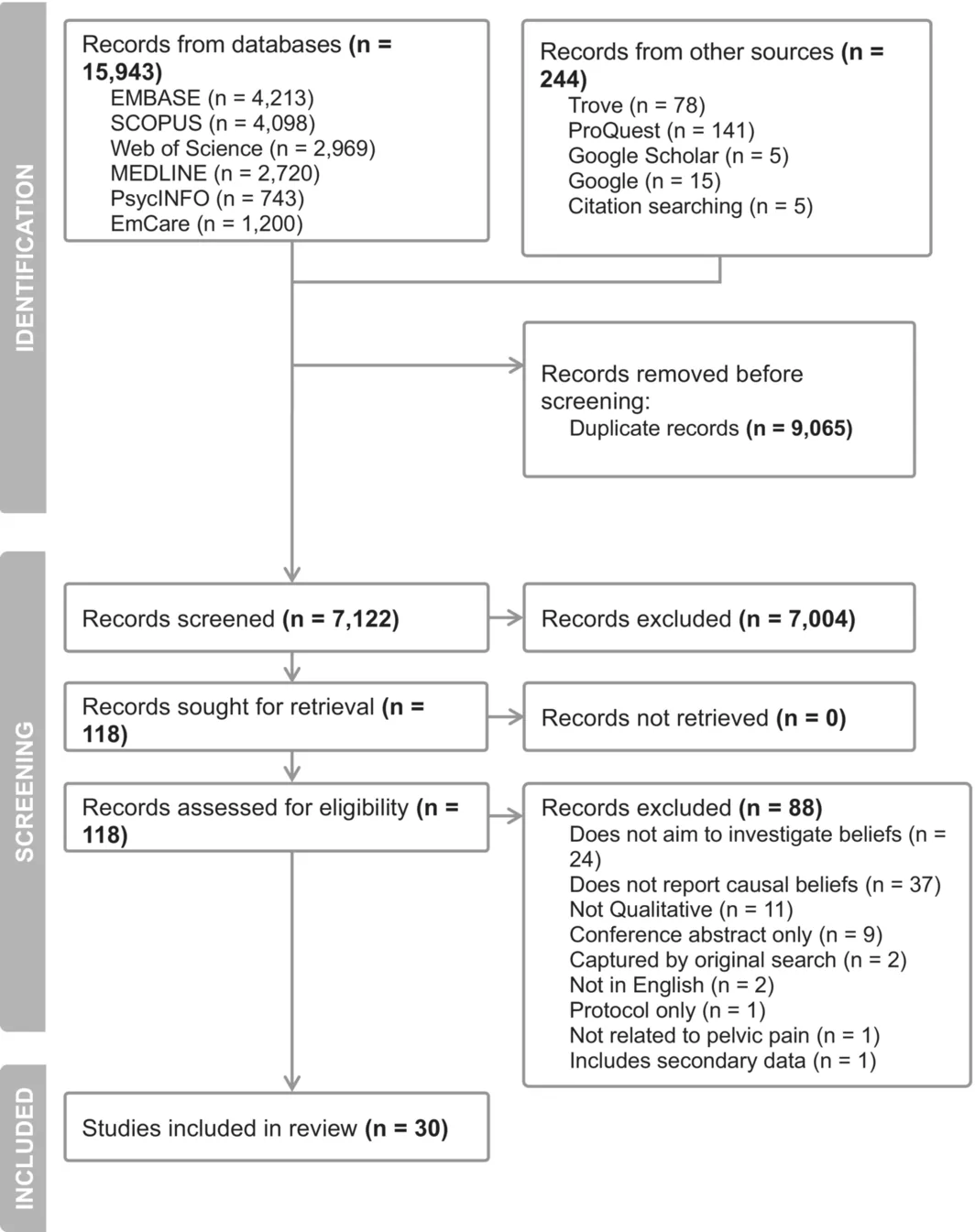
PRISMA flowchart.

The search strategy identified 15,943 records from online databases and 244 records from additional sources (Figure 1). After the removal of duplicates (*n* = 9065) and title and abstract screening (*n* = 7122), 118 records underwent full‐text screening (see Table S1 for excluded articles). Thirty articles, which included data from 1255 women, met the eligibility criteria and were included in the review.

### 3.2 Study Characteristics

An abridged table with the characteristics of the included studies is presented in Table 1 (see Table S2 for the full version). Twenty‐two pelvic pain conditions were reported by women included in the studies, with the most common being endometriosis (*n* = 13 studies), chronic pelvic pain (*n* = 8 studies), and vulvodynia (*n* = 5 studies). Ten countries were represented across the studies, with most being high‐income, western countries, including the United States of America (*n* = 7 studies), the United Kingdom (*n* = 6 studies), Norway (*n* = 5 studies), Canada (*n* = 3 studies), New Zealand (*n* = 2 studies), Australia (*n* = 2 study), and one study each from Ireland, Finland, Switzerland, and Brazil. Data were mostly collected via qualitative interviews (*n* = 26 studies). Participants were primarily Caucasian, cis‐gender, heterosexual, adult women. Three studies [2, 41, 62] had one participant who identified as agender; however, overarching terminology used within all studies remained as woman/women.

**TABLE 1:** Characteristics of included studies.

Article	Country	Study aim	Data collection method	Data analysis methodology	Sample size, age (years)	Diagnoses
Ballard et al. [40]	UK	Investigate the reasons for and impact of endometriosis diagnostic delay	Semi‐structured interviews	Thematic approach	*n* = 32 16–47	Endometriosis
Boge‐Olsnes et al. [41]	Norway	Investigate women's symptom and bodily distress perceptions in relation to their life experiences	Semi‐structured interviews	Phenomenological “discovery‐oriented” approach	*n* = 8 19–56	CPP
Boge‐Olsnes et al. [2]	Norway	Investigate women with CPP's experiences of Norwegian psychomotor physiotherapy	Individual semi‐structured interviews	Phenomenology	*n* = 8 19–56	CPP, endometriosis, IBS
Burbeck and Willig [42]	UK	Uncover the experience of dysmenorrhoea	Semi‐structured interviews	Interpretative phenomenological analysis	*n* = 7 24–36	Dysmenorrhoea
Chen et al. [43]	USA	Describe women's thoughts about their experiences of dysmenorrhoea	Cross‐sectional open‐ended online survey	Thematic analysis	*n* = 225 18–57	Secondary dysmenorrhoea, uterine fibroids, endometriosis, pelvic inflammatory disease
Crouch et al. [44]	Australia	Explore the beliefs and knowledge that females hold about their pelvic pain	Semi‐structured interviews	Interpretive description	*n* = 12 23–42	Endometriosis, dysmenorrhoea, vulvodynia, vaginismus, adenomyosis, prolapsed fibroid, CPP
Danielsen et al. [45]	Norway	Understand the therapeutic experience of women with provoked vestibulodynia undergoing somatocognitive therapy	Interviews	Thematic analysis	*n* = 6 22–37	Provoked vestibulodynia
de Jesus et al. [46]	Brazil	Understand diagnosed women's perspectives on the meaning of CPP and analyze determinants of outpatient discharge	Semi‐structured interviews and field observation	Content analysis	*n* = 14 mean = 42.5	CPP
Denny [47]	UK	Explore women's experience of living with endometriosis	Semi‐structured interviews, diary	Narrative analysis	*n* = 30	Endometriosis
Dogan et al. [48]	USA	Explore Black women's perceptions of their sexual pain and barriers which hinder disclosure to partners	Open‐ended responses from survey	Reflexive thematic analysis	*n* = 79 21–44	Dyspareunia
Donaldson and Meana [49]	USA	Explore experience of early dyspareunia symptoms in young women	Semi‐structured interviews	Grounded theory	*n* = 14 mean = 19.07	Dyspareunia
Girard et al. [50]	Switzerland	Provide a deeper understanding of women's views about endometriosis, fertility, and reproductive options	Semi‐structured interviews	Thematic content analysis	*n* = 11 19–39	Endometriosis
Glazer [51]	Canada	Understand interpretation and meaning of bodily pain and FGC from the perspective of women with FGC	Semi‐structured interviews, diary	Nomothetic analysis	*n* = 14 21–46	Type III FGC
Grace and MacBride‐Stewart [52]	New Zealand	Analyze women's reflections on ‘how come’ they have CPP	Open‐ended interviewing	Phenomenology with a biocultural approach	*n* = 40 22–51	CPP, dysmenorrhoea, dyspareunia
Groven et al. [53]	Norway	Explore women's experiences of vestibulodynia and its impact on daily living	Interviews	Thematic analysis	*n* = 8 23–32	Vestibulodynia
Hamilton [54]	USA	Develop a theory of coping among women with CPP	Interviews, daily tracking form	Grounded theory methodology	*n* = 15 20–44	CPP, endometriosis, IC, IBS, ovarian cysts, BPS, adhesions, PF dysfunction, uterine fibroids, vulvar vestibulitis, diastasis symphysis pubis
Helosvuori and Oikkonen [55]	Finland	Examine how interlocutors use embodied pain sensations to adapt to evolving evaluation and management context	Semi‐structured interviews	Collaborative inductive analysis with iterative theme refinement.	*N* = 26 Early 20s – late 40s	Endometriosis
Huntington and Gilmour [56]	New Zealand	Explore women's perceptions of living with endometriosis	Semi‐structured interviews	Thematic analysis	*n* = 18 16–45	Endometriosis
Katz et al. [57]	Australia	Investigate how women's perspectives relate to biopsychosocial factors	Survey with open ended questions	Template analysis	*n* = 383 18–49	Endometriosis
Katz [57]	USA	Explore the ways women make sense of their chronic vulvar pain	Narrative and semi‐structured interviews	Grounded theory technique and hermeneutic interpretation, comparative methods	*n* = 16 20–65	Chronic vulvar pain
LePage and Selk [58]	Canada	Identify unmet needs among localized provoked vulvodynia patients	Semi‐structured interviews	Descriptive thematic analysis, constant comparative method	*n* = 8 18–64	Localized, provoked vulvodynia
Lightbourne et al. [59]	Ireland	Examine women's perceptions of endometriosis care in Ireland	Semi‐structured interviews	Inductive reflexive thematic analysis	*n* = 20 18–50s	Endometriosis
Maena et al. [60]	Canada	Investigate causal attributions of women with dyspareunia and impact of this	Psychosocial interviews	Coding	*n* = 100 mean = 37.89	CPP, vulvar vestibulitis, vaginal atrophy
Myrtveit‐Stensrud et al. [61]	Norway	To explore how heterosexual couples experience living with vulvodynia	Semi‐structured interviews using an interview guide	Thematic analysis	*n* = 8 19–31	Vulvodynia (either localized, generalized, provoked, or unprovoked)
Pithavadian [62]	Australia	Understand women's help‐seeking experiences for their vaginismus	Semi‐structured interviews	Thematic analysis	*n* = 21 19–37	Vaginismus
Savidge et al. [63]	UK	Understand women's pain perceptions and helpful/unhelpful HCP interactions	Semi‐structured interviews	Qualitative data analysis	*n* = 21 21–61	CPP
Shallcross et al. [64]	UK	Explore the experiences of women with vulvodynia on their journey towards diagnosis	Semi‐structured interviews	Interpretative phenomenological analysis	*n* = 8 23–70	Vulvodynia
Twiss [65]	USA	Examine the lived experiences of three women living with CPP	Semi‐structured interviews	Chronological narratives, listening guide method	*n* = 3 25–30	CPP, endometriosis, ovarian cysts
Ward and Ogden [66]	UK	Examine feelings, experiences and cognitions of people living with vaginismus	Free‐text in questionnaires	Discourse analysis	*n* = 89 21–71	Vaginismus
Young and Miller [67]	USA	Examines how women with vulvodynia co‐construct and understand their condition	Observations of vulvodynia Facebook group members and open‐ended interviews	Thematic analysis	*n* = 11 23–65	Vulvodynia

Abbreviations: BPS, bladder pain syndrome; CPP, chronic pelvic pain; FGC, female genital circumcision; HCP, health care professional; IBS, irritable bowel syndrome; IC, interstitial cystitis; PF, pelvic floor; UK, United Kingdom; USA, United States of America; y, years.

### 3.3 Quality and Reporting Appraisal

The CASP checklist scores for each individual study are presented in Table 2. Across the studies, the average score was 9/10. Seven studies were rated as fully meeting all criteria outlined and were deemed to have minimal risk of bias. No studies addressed all criteria outlined in the COREQ checklist (see Table S3). All included studies except one [44] lacked preregistration and eight lacked ethics approval [42, 50, 54, 55, 60, 63, 65, 66]. The reporting quality of the included studies was overall very poor, predominately in areas involving disclosure of the researchers' experience, background, and relationships with participants. Additionally, details of the interview procedure, data management and participant transcript review were scarce.

**TABLE 2:** Quality appraisal using the CASP qualitative checklist (2023).

Article	1. Statement of aims	2. Appropriate methodology	3. Appropriate design for aims	4. Appropriate recruitment strategy	5. Appropriate data collection	6. Relationship consideration	7. Consideration of ethical issues	8. Rigorous data analysis	9. Clear statement of findings	10. Value of research	Total
Ballard et al. [40]	Y	Y	N	Y	Y	N	Y	Y	Y	Y	8
Boge‐Olsnes et al. [41]	Y	Y	Y	Y	Y	N	Y	Y	Y	Y	9
Boge‐Olsnes et al. [2]	Y	Y	N	Y	Y	Y	Y	Y	Y	Y	9
Burbeck and Willig [42]	Y	Y	Y	Y	Y	N	N	Y	Y	Y	8
Chen et al. [43]	Y	Y	N	Y	Y	N	Y	Y	Y	Y	8
Crouch et al. [44]	Y	Y	Y	Y	Y	Y	Y	Y	Y	Y	10
Danielsen et al. [45]	Y	Y	Y	Y	Y	N	Y	Y	Y	Y	9
de Jesus et al. [46]	Y	Y	Y	Y	Y	Y	Y	Y	Y	Y	10
Denny [47]	Y	Y	Y	Y	Y	Y	Y	Y	N	Y	9
Dogan et al. [48]	Y	Y	N	Y	Y	Y	Y	Y	Y	Y	9
Donaldson and Meana [49]	Y	Y	N	Y	Y	N	Y	Y	Y	Y	8
Girard et al. [50]	Y	N	N	N	Y	Y	N	Y	Y	Y	6
Glazer [51]	Y	Y	Y	Y	Y	Y	Y	Y	Y	Y	10
Grace and MacBride‐Stewart [52]	Y	Y	N	Y	Y	N	Y	Y	Y	Y	8
Groven et al. [53]	Y	Y	Y	Y	Y	Y	Y	Y	Y	Y	10
Hamilton [54]	Y	Y	Y	Y	Y	N	N	Y	Y	Y	8
Helosvuori and Oikkonen [55]	N	Y	N	Y	Y	N	N	N	Y	N	4
Huntington and Gilmour [56]	Y	Y	Y	Y	Y	N	Y	N	Y	Y	8
Katz et al. [57]	Y	Y	Y	Y	Y	NA	Y	Y	Y	Y	9
Katz [57]	Y	Y	Y	Y	Y	Y	Y	Y	Y	Y	10
LePage and Selk [58]	Y	Y	Y	Y	Y	Y	Y	Y	Y	Y	10
Lightbourne et al. [59]	Y	Y	Y	Y	Y	N	Y	Y	Y	Y	9
Maena et al. [60]	Y	Y	N	Y	Y	N	N	Y	Y	Y	7
Myrtveit‐Stensrud et al. [61]	Y	Y	N	Y	Y	Y	Y	Y	Y	Y	9
Pithavadian [62]	Y	Y	Y	Y	Y	N	Y	Y	Y	Y	9
Savidge et al. [63]	Y	Y	Y	Y	Y	N	N	Y	Y	Y	8
Shallcross et al. [64]	Y	Y	Y	Y	Y	Y	Y	Y	Y	Y	10
Twiss [65]	Y	Y	Y	Y	Y	Y	N	Y	Y	Y	9
Ward and Ogden [66]	Y	Y	Y	Y	Y	N	N	N	N	Y	6
Young and Miller [67]	Y	Y	N	Y	Y	Y	Y	Y	Y	Y	9

### 3.4 Data Synthesis

The following section describes the final themes generated through the synthesis process. Five hundred and fifteen unique concepts were derived from study narrative descriptions and participant quotes and organized into 85 categories (see Table S4 for example coding tree). Categories were refined into four generated themes: (1) It's happening to me because I'm a woman; (2) It's happening to me in my body; (3) It's happening to me due to my actions; and (4) It's happening to me due to my life. GRADE‐CERQual assessments for each theme are available in Table S6 [39].

#### 3.4.1 Theme 1: It's Happening to Me Because I'm a Woman

This theme describes how many women without a diagnosed pelvic pain condition frequently made sense of their pelvic pain as something which is “normal for women” [54]. Many described how their pelvic pain was a consequence of normal female biological processes, with one woman articulating that pelvic pain was “to do with women's basic internal organs” [52]. This normalization led some women to dismiss their pain as being “not that serious” [66] or something that must be suffered and endured. Whether or not women experienced pain from these normal biological functions was at times attributed to luck or chance

I just assumed I was just one of them unlucky people that got bad period pains Endometriosis [40].

In certain social groups, such as women who had undergone female genital circumcision, pain was normalized due to the cultural acceptance of this procedure [51]. Beyond those specific contexts, many women also emphasized the broader social aspects contributing to the normalization of their pelvic pain.

You're a woman! And that's what you have to go through! And you don't have a choice Chronic pelvic pain [52].

Throughout this theme, women described feeling that the cause of their pelvic pain was completely out of their control. Because they saw pain as a normal part of being a woman, they felt that the only option available was to accept it.

I always had painful periods, it's normal, all the women in my family have painful periods Endometriosis [50].

The perception that their pain was an inevitable part of being a woman left some participants feeling disempowered and out of control in managing their pain. Many often felt unable to access healthcare supports especially when they did not feel that their condition was valid enough to warrant treatment, with one woman saying, “I never really thought of it as a disease” [49]. However, most women were less likely to view their pain as normal if it developed later in life or increased in severity when compared to their previous pain‐free experiences.

Taken together, the theme ‘It's happening to me because I'm a woman’ describes how women frequently dismissed their pain as something which is normal for women to experience. This normalization left women feeling that their pelvic pain was out of their control because they could not change their female biology.

#### 3.4.2 Theme 2: It's Happening to Me in My Body

This theme describes how most women believed that their pelvic pain was caused by physiological factors within their body. Many women worried that their pain might be indicative of a sinister pathology, with one woman explaining, “I was afraid of having a more serious illness” [46]. Additionally, pain during intercourse was sometimes attributed to anatomical discrepancies such as “being too small down there” [53], with one woman describing, “not quite in the opening, but just a bit further in there's a kind of barrier” [41]. These predominately biological beliefs were often accompanied by participants reporting feeling they lacked knowledge about pelvic pain, with many voicing their uncertainty around the cause of their pain

I didn't know what it was, what it means. I still know nothing about it Localised, provoked vulvodynia [58].

Women often used biological guesses and labels to define and validate their pain experiences, with some believing that diagnosis was the only avenue to receiving appropriate health care management: “I know my body. Just because you don't see [yeast] on a microscope doesn't mean it isn't there” [54].

Women also described feeling that their internal physiology causing their pain was outside of their control. For instance, many attributed their pain to internal bodily processes such as “endometriosis flaring up” [40]. Subsequently, these participants often felt their symptoms were out of their control and could only be influenced by interventions administered by a medical practitioner, as opposed to feeling able to independently self‐manage their condition. They also described feeling that their body, or painful body part, was “broken” [57], which further diminished their sense of control and alienated them from their own bodies.

Pain was often described as something which was located within the painful body part, with many women believing that the surgical removal of this body part would cure their pain. However, of those who had undergone surgery to remove their painful body part, they largely reported that their pain remained the same or worsened.

I had a hysterectomy on 6th December, and now I am regretting this as six months on I am feeling no better at all, in fact I feel worse Endometriosis [47].

Taken together, the theme ‘It's happening to me in my body’ describes how many women believed that their pelvic pain was caused by internal physiological factors, leaving them feeling out of control over their pain and requiring them to rely on external support from healthcare professionals.

#### 3.4.3 Theme 3: It's Happening to Me due to My Actions

In this theme, many women noticed ways in which their actions could influence their pain experience, both positively and negatively. For example, some women attributed their pain to certain movements or physical activity, and this often led to fear‐avoidant behaviors and reduced participation in activities: “On the first day of my period if I have to do something where I have to use my abdominal muscles, then that really sets off the pain” [42]. Some women even worried that their pain might be caused by them being “bad at sex” [61].

As sex was painful every time… I reasoned something had to be wrong… figured I was not prepared, maybe not relaxed enough Vestibulodynia [53].

This self‐observed correlation between pain and actions left some women feeling disempowered. Many acknowledged the emotional burden associated with their belief that they were at fault or contributing to their pain, with one woman saying, “What have I been doing wrong?” [54]. Believing that they were somewhat responsible for their pain left some women feeling helpless and unable to influence their pain management.

I was even telling myself; I'm stressed, I'm somatising pain in my belly – Endometriosis [50].

I thought that it was my fault… I was to blame, in a way. I am the one stressing Vestibulodynia [53].

In the above quotes, participants articulate their belief that they are at fault in relation to stress. Women who felt responsible for the cause of their pain frequently described feeling uncertain about what pain management strategies may be beneficial for them. This sometimes left them feeling unable to access healthcare support as their condition did not familiarly fit within the biomedical paradigm of western healthcare.

However, some women felt empowered in noticing that their actions could influence their pain experience. For example, “I could feel that I was actually tensing my abdomen almost all the time. So, then I started to realise it more, and maybe it made me breathe a lot more as well. I tried to relax because I wanted it to stop hurting. And then it got a lot better” [2]. This awareness and proactive approach to managing their pelvic pain offered these women a greater sense of control and improvement in outcomes.

Taken together, the theme ‘It's happening to me due to my actions’ describes how many women noticed that their actions could influence their pelvic pain. While these beliefs made many women feel out of control and a sense of self‐blame, some women began to develop ways of managing their pelvic pain through their actions and developed a greater sense of control over their pelvic pain.

#### 3.4.4 Theme 4: It's Happening to Me due to My Life

This theme describes how some women attributed their pelvic pain to life events. Several women described specific past events, including previous medical interventions to which they attributed the cause of their pelvic pain and considered outside of their control. One woman described: “I had glandular fever and from then on, I was in pain 24/7 no matter what was happening” [56]. While these women saw past events as causal for their pelvic pain, sometimes they discussed how emotional distress related to the traumatic event also had a role in their pain.

I was abused before I was 16 and people didn't believe me, and then I gradually started to get stabbing pain in my pelvis. I suppose it was because I was so tense after what happened to me CPP [41].

Some women attributed the cause of their pelvic pain to the past traumatic experiences, describing their pain as a protective response from these events: “The reason I have pain during intercourse is because I overstepped my own boundaries as a teenager” [61]. Often these participants were more open to psychosocial intervention as they had already made the link between their pain and psychosocial factors. However, many women still felt that they had no control over their pain as they could not take away the traumatic experience.

I believe I developed vaginismus at a very young age as a defence to a controlling mother who wouldn't allow me to ‘separate’ into another human being. I put a false physical barrier around myself to provide a safe retreat, safe from invasion, and to prevent merger with her, i.e., disintegration of self Vaginismus [66].

Additionally, some believed that their traumatic experienced had caused a physiological condition which required biomedical management. This meant that despite acknowledging psychosocial causal factors, these women did not see the benefit in psychosocial elements to pain rehabilitation, such as psychological therapy.

Some women noticed a connection between their pelvic pain experience and aspects of their life at present such as “having a stressful day at work” [62]. For some, this led them to develop avoidance behaviors regarding elements of their life that they saw as contributing to their pain. As demonstrated by the following quote, some women noticed how their relationships impacted on their experience of pelvic pain:

I would turn the question around to say it is the family relationship that affects the vaginismus rather than vice versa Vaginismus [66].

Women who had noticed a connection between their present life and their pain experiences more often described a biopsychosocial understanding of their pelvic pain and were frequently already integrating psychosocial management strategies into their personal models of care.

When I get stressed or run down, then I notice it flares up so I now know what it is and can adapt round it when I know it's starting to flare Chronic pelvic pain [63].

Taken together, the theme, ‘It's happening to me due to my life’ describes how some women attributed the cause of their pelvic pain to life events. Many women attributed their pain to past medical or traumatic experiences which they could not change leaving them feeling out of control of their pelvic pain. However, some women noticed how alterations to their environment enabled them to positively influence and gain more control over their pelvic pain.

### 3.5 Pelvic Pain: A Triad of Understanding and Sense of Control

**FIGURE 2:**
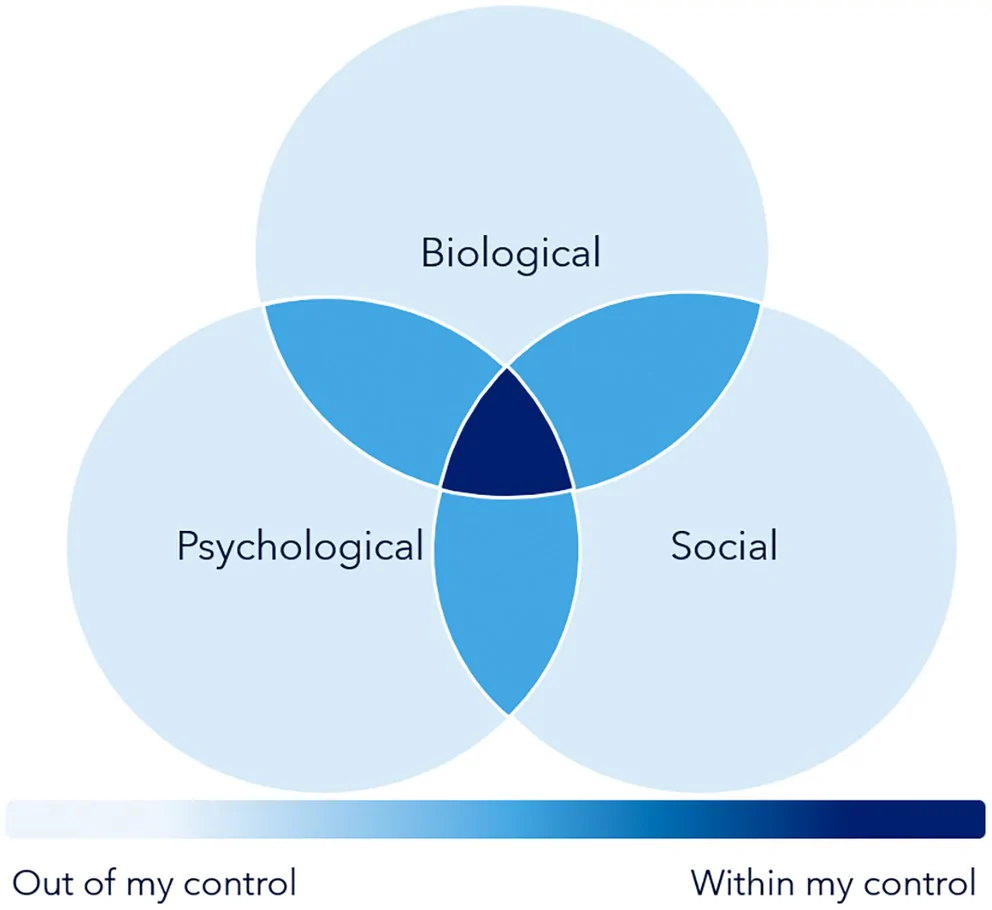
Pelvic pain: A triad of understanding and sense of control.

A conceptual model was developed (Figure 2) to provide a scaffold within which all causal beliefs found by this meta‐ethnography can be represented. The four themes reflected the multifaceted nature of women's causal beliefs about their pelvic pain, encompassing biological, psychological, and social contributors. Accordingly, the findings were interpreted through the lens of the biopsychosocial model [18]. Across the four themes, women's causal beliefs about their pelvic pain ranged from siloed biological, psychological, or social explanations to multidimensional perspectives incorporating a combination of these components. These beliefs also varied in terms of perceived control over their pain, from feeling entirely powerless to experiencing a sense of personal agency.

For example, one participant with chronic pelvic pain expressed her symptoms solely through a biological lens, describing her pain as “to do with women's basic internal organs” [52]. This perspective aligns with themes one and two, reflecting a sense of limited personal control because her symptoms were seen as inherently tied to female biology and therefore outside of the scope of self‐management.

Another participant with endometriosis highlighted the influence of her social environment on her psychological well‐being and how these two factors might contribute to her symptoms. She reflected, “I was in a very stressful job–was my job something to do with it? Am I psychologically making myself have this pain?” [40]. This perspective aligns with themes three and four, and while she voiced her uncertainty, she also discussed factors in her life over which she might have the power to influence her symptoms.

Finally, a participant with chronic pelvic pain observed how her physical and mental health were distinctly connected to her symptoms, explaining “when I get stressed or run down, then I notice it flares up so I now know what it is and can adapt round it when I know it's starting to flare” [63]. Taking this understanding further, this participant integrated biological, psychological, and social factors to actively manage her symptoms, demonstrating a greater sense of control over her health and well‐being.

Only one study explicitly reported beliefs aligned with a contemporary, biopsychosocial understanding of pain, with some participants recognizing the dynamic interplay of factors which contribute to pain persisting: “So many different systems, so many different inputs are all contributing to the response of pain, and those responses then influence our inputs and continue to alter that cycle, continue to alter our response and our output of pain” [44]. No other studies in the sample captured similar participant causal beliefs.

The conceptual model allows for change across this spectrum of control. Women described how their understanding of the various factors contributing to their pain evolved from a singular focus on biological, psychological, or social explanations to a recognition of its multifaceted nature. Similarly, women noted how this evolving understanding empowered them to transition from feeling that their pelvic pain was beyond their control to believing they could exert some influence over their pain management, healthcare decisions, and their lives.

## 4 Discussion

This meta‐ethnography identified and synthesized the current literature on women's causal beliefs about their pelvic pain. Four main themes were identified: it's happening to me because I'm a woman; it's happening to me in my body; it's happening to me due to my actions; and it's happening to me due to my life. Women's pain attributions varied from single‐dimensional biological, psychological, or social explanations to combinations of these factors. Their perceptions of control also ranged from feeling that their pain was ‘out of their control’ to ‘within their control’. A conceptual model was developed to represent this spread of causal beliefs that women hold about their pelvic pain, and how those beliefs may contribute to women's overall feeling of control over their pain.

Women in this meta‐ethnography acknowledged multiple diverse elements which contributed to their pain; however, biological factors were frequently implied to be the underlying cause. One possible reason for an emphasis on biological causal beliefs among women with pelvic pain is a misalignment of their pain beliefs with contemporary concepts in pain science which may reflect broader limitations in pain education and communication within the healthcare system and society [22]. This was reflected in this meta‐ethnography where women frequently voiced their feelings of uncertainty regarding the cause of their pain. Notably, one study included in this review reported women holding causal beliefs more consistent with contemporary pain science [44]; it is possible that, given the study was conducted by a pain science research group, participants in this sample may have had greater exposure to pain science concepts than is typical. Additionally, it has been demonstrated in lower back pain patients that health care professionals have a strong influence on patients' beliefs and may contribute to their biological conceptualisation of pain [68]. Further to this, individuals' beliefs about pain have shown to influence their behavior, including how they seek help and the types of pain management they use [69]. For women with pelvic pain, the diagnostic process is highly medicalised and biological in nature, with a strong focus on surgical and pharmacological interventions [16]. Within this meta‐ethnography women with pelvic pain often used medical labels to define their pain to gain medical and societal recognition of their condition. Given that the western medical system in which these women predominantly seek help is highly biomedical, and biological diagnoses validate these women's pain [70], it is unsurprising that women would continue to hold biological causal beliefs. Conceptualizing pain through a biological lens is not unique to pelvic pain and has been described in other chronic pain conditions including musculoskeletal and back pain [21, 71, 72]. This biological conceptualisation may influence how individuals understand the cause of their pain and their approach towards pain management.

Women in this meta‐ethnography were aware that certain aspects of their pelvic pain might be controllable, however, they were largely uncertain how to claim control over their pain. One possible reason for this is the association between biological causes and external management, as described in studies of musculoskeletal and back pain [21, 68, 73]. In this meta‐ethnography, women often believed that their pain was caused by a pathology and therefore required medical or surgical intervention from an expert health care professional. This belief likely fosters a perception that pain control must come from external sources (i.e., the surgeon needs to remove the pathology to alleviate the pain) [40]. Furthermore, another meta‐ethnography highlighted how diagnostic delays leave women with pelvic pain feeling in limbo and reliant on their health care professionals when seeking a diagnosis for their pain, which is likely to further increase their uncertainty and reliance on external control [70]. In a prospective qualitative investigation of individuals with low back pain, there was a tendency among participants to view more controllable psychosocial variables as pain moderators, rather than causative factors [73]. This perspective is evident among women with endometriosis, where psychosocial factors were perceived as contributors to pain, yet psychosocial management was viewed as redundant because of the belief that pain was directly caused by the pathological endometriosis lesions [74]. This belief that pain is caused by factors outside of individuals' control is well documented in people with pelvic pain [25, 74], as well as other chronic pain conditions such as back pain [73], and feelings of powerlessness are often described. This suggests that feeling unable to claim control over one's healthcare journey is not unique to pelvic pain and is instead a chronic pain wide issue.

Women with pelvic pain generally describe biological causal beliefs and feeling out of control with their pain management. In this meta‐ethnography, women who believed that their pain was caused by multiple interconnected factors typically described feeling more in control of their pain, healthcare, and life. This sense of control may translate to better outcomes, as reflected across both general health and chronic pain populations. Within the general population, greater perceived control has been associated with improved mental health outcomes [75]. In chronic pain populations, greater perceived control has been linked to increased life participation and reduced pain perception [76, 77]. Furthermore, helping individuals with chronic musculoskeletal pain understand the multifaceted nature of pain beyond its biological aspects has been shown to reduce pain and improve function [78]. Taken together, these findings suggest that helping women with pelvic pain to transition towards a multifactorial understanding of health and wellbeing may increase their sense of control and aid them to make decisions that improve their pain.

The findings of this meta‐ethnography and the conceptual model presented have important clinical implications. Empowering women to understand the multifaceted contributors to their pain may foster greater control of their pain. Knowing where a patient is situated on the conceptual model may help guide personalized treatment approaches that match their beliefs. Previous research has shown that causal beliefs in people with chronic pain can be modified through pain science education, in turn resulting in increased knowledge about pain and improved functional capacity [23, 78]. A key step in pain science education is identifying and addressing patients' current beliefs about their pain [79]. Among women with pelvic pain, preliminary data suggest that pain science education can also improve pain outcomes when combined with physiotherapy intervention [22, 24, 80]. By synthesizing evidence on women's causal beliefs about their pelvic pain, this research directly informs the refinement of pain science education approaches in this population. To re‐educate causal beliefs in women with pelvic pain, an earlier introduction of the multifactorial contributors to pain may be necessary. Educational programs provided in schools, such as the Pelvic Pain Foundation of Australia's PPEP Talk [81], may be one avenue for earlier introduction of contemporary pain science principals to shape the way that girls and young women, as well as their social supports and healthcare professionals, think about pelvic pain. Finally, all studies included in this meta‐ethnography were conducted in western countries with traditionally more biomedically orientated healthcare systems. It is important to acknowledge that the nature of the western medicine healthcare model has the potential to teach and reinforce biological health beliefs [68]. Therefore, systemic change based on a biopsychosocial understanding of health and illness may be required to ensure women's experiential learnings reinforce biopsychosocial understandings of pelvic pain.

### 4.1 Strengths, Limitations, and Future Research

This meta‐ethnography has clear strengths. This review followed rigorous methodology; guidance was taken from Sattar et al. [28] and the study was reported in alignment with the PRISMA 2020 checklist [29] and the eMERGE meta‐ethnography reporting guidance [30]. It was registered prospectively on Open Science Framework and PROSPERO [31]. A reflexive journal was kept by the primary researcher during the data analysis process to maintain a written record of the iterative coding process and details of decisions and influences that occurred throughout the coding process. Themes were represented across all studies, irrespective of methodological quality (see Table S5 for sensitivity analysis). This strengthens confidence in the robustness of the findings. The included studies also had clear strengths. The average CASP score was 9/10, with seven studies deemed to have met all CASP criteria and considered to pose minimal risk of bias. Overall, sample characteristics were provided appropriately, increasing study transferability, and the inclusion of participant quotes reinforced study confirmability [82]. Disparate codes were discussed throughout study results, adding to the credibility of included studies [82].

This meta‐ethnography, however, is not without limitations. Non‐English language studies were excluded from this review, and this was reflected in the largely western nature of included studies and the participant sample, which was predominately Caucasian, cis‐gender, heterosexual women of reproductive age. As a result, relevant records in other languages may have been missed, and this sample may not align with the beliefs of culturally and ethnically diverse populations. Cultural and social contexts, such as prevailing health beliefs, gender norms, and access to healthcare, are likely to influence how individuals interpret and respond to pelvic pain [83]. Given the limited demographic diversity in the included studies, the findings and conceptual model may not fully capture these influences, potentially limiting their generalisability to non‐Western populations or gender‐diverse individuals. Poor reporting, particularly regarding reflexivity and positionality, limited the interpretive depth of individual studies and therefore of this synthesis. To mitigate this, the analysis prioritized primary data in the form of participant quotations. Using quotations ensured that the synthesis was grounded in participants' own accounts, reducing the risk that gaps or biases in authors' interpretations obscured important perspectives. The limitations of the studies included in this meta‐ethnography are also worth noting. Dependability and credibility were influenced by the lack of protocols and ethics approval which increases the risk of bias and reduces transparency [84, 85]. The confirmability of the studies was influenced by a lack of assessment into how the researcher's own context, perceptions, and interests may influence the research [82, 86]. The transferability of the studies was reduced through the lack of clear reporting, including interview procedures and data management [82]. One study [2] frequently used language which misgendered a participant who identified as agender. Future studies should ensure inclusive language to avoid misgendering, and consider possible differences in beliefs of cisgender and gender diverse individuals.

The primary researcher acknowledges that her own context as a cisgender, white woman, living in a western country, analyzing beliefs from women of predominantly the same background, limits the transferability of these results beyond this cohort. Furthermore, her clinical experience with chronic pain conditions and seeing patients benefit from a biopsychosocial approach has contributed to her deep‐rooted understanding and belief in a biopsychosocial model of pain. The wider research team has extensive research experience and is from a largely allied health background which informs the methodology and health care focus of this study. Additionally, the research team is affiliated with institutions that advocate biopsychosocial practice as gold standard, and this informed their largely biopsychosocial viewpoints.

Future research may investigate how causal beliefs about pelvic pain relate to clinical outcomes and explore whether interventions addressing women's misconceptions about their pain can result in better pain outcomes. Additionally, future research should embody greater participant diversity, including minority populations, in understanding women's pelvic pain experiences.

## 5 Conclusion

Women with pelvic pain frequently describe causal beliefs relating to a single, often biological, factor and report feeling out of control with their pain management. However, when women recognize and understand the multifaceted nature of their pain, they also tend to feel greater control over their symptoms. These findings suggest that through understanding the causal beliefs held by women regarding their pelvic pain, clinicians may be able to more appropriately tailor pain management interventions to align with patients' perspectives and needs. This can, in turn, empower women to feel more in control of their pelvic pain and provide a foundation for recovery. This may provide scope for reducing the persistent nature of chronic pelvic pain.

## Author Contributions

G.A.F. contributed to the development of the protocol and search strategy, conducted the search of databases, record screening, data extraction, quality assessment, data analysis, and drafting of the paper. A.K.M. contributed to the conception and design of the study, development of the protocol and search strategy, record screening, data extraction, quality assessment, data analysis, and drafting of the paper. A.M.M. contributed to the development of the protocol and search strategy, record screening, data extraction, quality assessment, data analysis, and drafting of the paper. S.K. contributed to the development of the protocol and search strategy, record screening, data analysis, and drafting of the paper. K.J.C. contributed to the conception and design of the study, development of the protocol and search strategy, record screening, data extraction, quality assessment, data analysis, and drafting of the paper. All authors have read and approved the final manuscript.

## Funding

This research did not receive any specific grant from funding agencies in the public, commercial, or not‐for‐profit sectors.

## Disclosure

This study has received no financial support; no funding bodies played any role in developing this study. K.J.C. is supported by a Leadership Investigator grant to Professor Moseley from the National Health & Medical Research Council of Australia (ID 1178444). A.K.M. is supported by a grant awarded to the NICM Health Research Institute, Western Sydney University. A.M.M. is supported by the Research Training Program domestic (RTPd) Stipend as a postgraduate student at the University of Melbourne. S.K. and G.A.F. declare no funding support related to this research. K.J.C. and A.K.M. have received payment for presentations given on pelvic health and pain, and professional bodies have reimbursed them for related travel costs. A.M.M., S.K., and G.A.F. report no conflicts of interest. *Statement on the use of artificial intelligence*: Artificial Intelligence tools were not used to aid the preparation of this manuscript.

## Ethics Statement

Since this project is a systematic review and meta‐synthesis of secondary and de‐identified data, ethics approval is not required.

## Consent

The authors have nothing to report.

## Conflicts of Interest

K.J.C. has received payment for presentations given on pelvic health and pain. A.K.M. has received speaker fees for lectures on pelvic pain. Professional bodies have reimbursed her for travel costs related to the presentation of research on pelvic pain at scientific conferences. A.M.M., S.K., and G.A.F. report no conflicts of interest.

## Supporting information


**Table S1:** Excluded records.
**Table S2:** Characteristics of included studies.
**Table S3:** Appraisal of reporting using the COREQ checklist [37].
**Table S4:** Coding tree.
**Table S5:** Sensitivity analysis.
**Table S6:** GRADE‐CERQual analysis.
**File S1:** Search strategies.

## Data Availability

Dataset sharing not applicable—no new data generated.
