# Supplementary material for: Women's Causal Beliefs About Their Pelvic Pain: A Systematic Review and Meta‐Ethnography
Source: Pain Pract. 2026 Aug 28;26(7):e70194. doi: 10.1111/papr.70194 (PMC13523623; doi:10.1111/papr.70194)
Supplement: Supplementary file 1 [file PAPR-26-0-s001.docx]

# File S1. Search strategies

**MEDLINE**

1	Chronic Pain/

2	((chronic or persist*) adj3 pain).mp. [mp=title, book title, abstract, original title, name of substance word, subject heading word, floating sub-heading word, keyword heading word, organism supplementary concept word, protocol supplementary concept word, rare disease supplementary concept word, unique identifier, synonyms, population supplementary concept word, anatomy supplementary concept word]

3	1 or 2

4	exp Pelvic Pain/

5	((pain* or congest*) adj3 (gynaecolog* or sex* or intercourse* or period or perineal or genit* or cycl* pelvic or pelvic* or cyst* or interstitial cyst* or bladder or pyelocyst* or vulv* or ovulat*)).mp. [mp=title, book title, abstract, original title, name of substance word, subject heading word, floating sub-heading word, keyword heading word, organism supplementary concept word, protocol supplementary concept word, rare disease supplementary concept word, unique identifier, synonyms, population supplementary concept word, anatomy supplementary concept word]

6	exp Cystitis/

7	exp Vulvitis/

8	Endometriosis/

9	Vulvodynia/

10	Dyspareunia/

11	Dysmenorrhea/

12	Vaginismus/

13	Adenomyosis/

14	Pudendal Neuralgia/

15	(vestibulitis or vestibulodynia or mittelschmerz or ovulationmittelschmerz or endometrio* or dyspareunia or dysmenorrh?ea or vaginismus or adenomyosis or pudendal neuralgia).mp. [mp=title, book title, abstract, original title, name of substance word, subject heading word, floating sub-heading word, keyword heading word, organism supplementary concept word, protocol supplementary concept word, rare disease supplementary concept word, unique identifier, synonyms, population supplementary concept word, anatomy supplementary concept word]

16	Or/4-15

17	Perception/

18	Pain Perception/

19	Psychology/

20	Health Knowledge, Attitudes, Practice/

21	(knowledg* or belief* or understand* or attitude* or perception* or misconcept* or psycholog* or psychosocial or "psychosocial effect*").mp. [mp=title, book title, abstract, original title, name of substance word, subject heading word, floating sub-heading word, keyword heading word, organism supplementary concept word, protocol supplementary concept word, rare disease supplementary concept word, unique identifier, synonyms, population supplementary concept word, anatomy supplementary concept word]

22	Or/17-21

23	3 and 16 and 22

**EMBASE/EmCare**

1 chronic pain/
2 ((chronic or persist*) adj3 pain).mp. [mp=title, book title, abstract, original title, name of substance word, subject heading word, floating sub-heading word, keyword heading word, organism supplementary concept word, protocol supplementary concept word, rare disease supplementary concept word, unique identifier, synonyms, population supplementary concept word, anatomy supplementary concept word]
3 1 or 2
4 exp pelvic pain/
5 ((pain* or congest*) adj3 (gynaecolog* or sex* or intercourse* or period or perineal or genit* or cycl* pelvic or pelvic* or cyst* or interstitial cyst* or bladder or pyelocyst* or vulv* or ovulat*)).mp. [mp=title, book title, abstract, original title, name of substance word, subject heading word, floating sub-heading word, keyword heading word, organism supplementary concept word, protocol supplementary concept word, rare disease supplementary concept word, unique identifier, synonyms, population supplementary concept word, anatomy supplementary concept word]
6 exp cystitis/
7 exp vulvitis/
8 endometriosis/
9 vulvodynia/
10 dyspareunia/
11 dysmenorrhea/
12 vaginismus/
13 adenomyosis/
14 pudendal neuralgia/
15 (vestibulitis or vestibulodynia or mittelschmerz or ovulationmittelschmerz or endometrio* or dyspareunia or dysmenorrh?ea or vaginismus or adenomyosis or pudendal neuralgia).mp. [mp=title, book title, abstract, original title, name of substance word, subject heading word, floating sub-heading word, keyword heading word, organism supplementary concept word, protocol supplementary concept word, rare disease supplementary concept word, unique identifier, synonyms, population supplementary concept word, anatomy supplementary concept word]
16 or/4-15
17 perception/
18 psychology/
19 attitude to health/
20 (knowledg* or belief* or understand* or attitude* or perception* or misconcept* or psycholog* or psychosocial or "psychosocial effect*").mp. [mp=title, book title, abstract, original title, name of substance word, subject heading word, floating sub-heading word, keyword heading word, organism supplementary concept word, protocol supplementary concept word, rare disease supplementary concept word, unique identifier, synonyms, population supplementary concept word, anatomy supplementary concept word]
21 or/17-20
22 3 and 16 and 21

**PsychINFO**

1 Chronic Pain/
2 ((chronic or persist*) adj3 pain).mp. [mp=title, book title, abstract, original title, name of substance word, subject heading word, floating sub-heading word, keyword heading word, organism supplementary concept word, protocol supplementary concept word, rare disease supplementary concept word, unique identifier, synonyms, population supplementary concept word, anatomy supplementary concept word]
3 23 or 24
4 ((pain* or congest*) adj3 (gynaecolog* or sex* or intercourse* or period or perineal or genit* or cycl* pelvic or pelvic* or cyst* or interstitial cyst* or bladder or pyelocyst* or vulv* or ovulat*)).mp. [mp=title, book title, abstract, original title, name of substance word, subject heading word, floating sub-heading word, keyword heading word, organism supplementary concept word, protocol supplementary concept word, rare disease supplementary concept word, unique identifier, synonyms, population supplementary concept word, anatomy supplementary concept word]
5 Dyspareunia/
6 Dysmenorrhea/
7 Vaginismus/
8 (vestibulitis or vestibulodynia or mittelschmerz or ovulationmittelschmerz or endometrio* or dyspareunia or dysmenorrh?ea or vaginismus or adenomyosis or pudendal neuralgia).mp. [mp=title, book title, abstract, original title, name of substance word, subject heading word, floating sub-heading word, keyword heading word, organism supplementary concept word, protocol supplementary concept word, rare disease supplementary concept word, unique identifier, synonyms, population supplementary concept word, anatomy supplementary concept word]
9 or/26-30
10 Perception/
11 Pain Perception/
12 Psychology/
13 Health Knowledge/
14 Health Attitudes/
15 (knowledg* or belief* or understand* or attitude* or perception* or misconcept* or psycholog* or psychosocial or "psychosocial effect*").mp. [mp=title, book title, abstract, original title, name of substance word, subject heading word, floating sub-heading word, keyword heading word, organism supplementary concept word, protocol supplementary concept word, rare disease supplementary concept word, unique identifier, synonyms, population supplementary concept word, anatomy supplementary concept word]
16 or/32-37
17 25 and 31 and 38

**SCOPUS**

TITLE-ABS-KEY ( ( chronic  OR  persist* )  W/3  pain )  AND  TITLE-ABS-KEY ( ( pelvi*  W/3  pain )  OR  ( ( pain*  OR  congest* )  W/3  ( gynaecolog*  OR  sex*  OR  intercourse*  OR  period  OR  perineal  OR  genit*  OR  cycl*  OR  pelvic*  OR  cyst*  OR  interstitial  OR  bladder  OR  pyelocyst*  OR  vulv*  OR  ovulat* ) )  OR  cystitis  OR  vulvitis  OR  endometrio*  OR  vulvodynia  OR  dyspareunia  OR  dysmenorrh?ea  OR  vaginismus  OR  adenomyosis  OR  "pudendal neuralgia"  OR  vestibulitis  OR  vestibulodynia  OR  mittelschmerz )  AND  TITLE-ABS-KEY ( perception  OR  "pain perception"  OR  knowledg*  OR  belief*  OR  understand*  OR  attitude*  OR  misconcept*  OR  psycholog*  OR  psychosocial  OR  "psychosocial effect*"  OR  ( health  W/2  ( knowledge  OR  attitudes  OR  practice ) ) )

**Web of Science** **Core Collection**

(TS=((chronic OR persist*) near/3 pain))

**AND**

((TS=(pelvi* near/3 pain)) OR TS=((pain* OR congest*) near/3 (gynaecolog* OR sex* OR intercourse* OR period OR perineal OR genit* OR cycl* OR pelvic* OR cyst* OR interstitial OR bladder OR pyelocyst* OR vulv* OR ovulat*))) OR TS=(cystitis OR vulvitis OR endometrio* OR vulvodynia OR dyspareunia OR dysmenorrh?ea OR vaginismus OR adenomyosis OR "pudendal neuralgia" OR vestibulitis OR vestibulodynia OR mittelschmerz)

**AND**

(TS=(perception OR "pain perception" OR knowledg* OR belief* OR understand* OR attitude* OR misconcept* OR psycholog* OR psychosocial OR "psychosocial effect*")) OR TS=(health near/2 (knowledge OR attitudes OR practice))

**ProQuest  Dissertations and Theses Global**

TITLE-ABS-KEY ( ( chronic  OR  persist* )  N/3  pain )  AND  TITLE-ABS-KEY ( ( pelvi*  N/3  pain )  OR  ( ( pain*  OR  congest* )  N/3  ( gynaecolog*  OR  sex*  OR  intercourse*  OR  period  OR  perineal  OR  genit*  OR  cycl*  OR  pelvic*  OR  cyst*  OR  interstitial  OR  bladder  OR  pyelocyst*  OR  vulv*  OR  ovulat* ) )  OR  cystitis  OR  vulvitis  OR  endometrio*  OR  vulvodynia  OR  dyspareunia  OR  dysmenorrh?ea  OR  vaginismus  OR  adenomyosis  OR  "pudendal neuralgia"  OR  vestibulitis  OR  vestibulodynia  OR  mittelschmerz )  AND  TITLE-ABS-KEY ( perception  OR  "pain perception"  OR  knowledg*  OR  belief*  OR  understand*  OR  attitude*  OR  misconcept*  OR  psycholog*  OR  psychosocial  OR  "psychosocial effect*"  OR  ( health  N/2  ( knowledge  OR  attitudes  OR  practice ) ) )

**Trove** **Australian online database aggregator and service**

“pelvic pain” and belief* 35

“pelvic pain” and knowledg* 41

“pelvic pain” and perception* 28

“pelvic pain” and attitude* 26

“pelvic pain” and psychosocial 23

“pelvic pain” and understand* 46

“pelvic pain” and psycholog* 33

**Google**

Pelvic pain knowledge and beliefs

**Google Scholar**

Experience AND Vaginismus

   Endometriosis

   Vulvodynia

   Vulvitis

   Dyspareunia

   Dysmenorrhea

   Adenomyosis

   Pudendal neuralgia

   Cystitis

   Mittelschmerz

**Organisations**

Pelvic Pain Foundation

Endometriosis Australia

International Pelvic Pain Society

World Endometriosis Society

National Vulvodynia Association

QUENDO

Jean Hailes

Pelvic Pain Support Network

# Table S1. Excluded records

| Study | Reason for exclusion |
| --- | --- |
| Adams et al. (2025) | Not qualitative |
| Adams et al. (2024) | Not qualitative |
| Ayling & Ussher (2008) | Does not report causal beliefs |
| Blair et al. (2015) | Not qualitative |
| Boge-Olsnes et al. (2024) | Does not report causal beliefs |
| Boge-Olsnes et al. (2023) | Captured by original search |
| Brauer et al. (2014) | Does not report causal beliefs |
| Brown et al. (2024) | Does not aim to investigate beliefs |
| Bullo & Hearn (2024) | Does not aim to investigate beliefs |
| Bullo & Hearn (2021) | Does not report causal beliefs |
| Burke et al. (2024) | Does not aim to investigate beliefs |
| Chen et al. (2018) | Does not report causal beliefs |
| Cole et al. (2021) | Does not report causal beliefs |
| Coxon et al. (2024) | Not qualitative |
| Danielsen et al. (2024) | Does not aim to investigate beliefs |
| De C. Williams et al. (2025) | Does not aim to investigate beliefs |
| Denny & Mann (2008) | Thoughts/beliefs not related to their pain |
| Dogan et al. (2023) | Captured by original search |
| Dowding et al. (2024) | Does not aim to investigate beliefs |
| Dowding et al. (2025) | Does not report causal beliefs |
| Elcombe et al. (1997) | Not qualitative |
| Ellis et al. (2023) | Does not report causal beliefs |
| Ellis & Wood (2025) | Does not aim to investigate beliefs |
| Emad et al. (2006) | Does not report causal beliefs |
| Engel & Hoffmann (1994) | Not qualitative |
| Fallon (2025) | Does not aim to investigate beliefs |
| Fernandez-Martinez et al. (2020) | Does not report causal beliefs |
| Fry et al. (1991) | Not qualitative |
| Gouesbet et al. (2023) | Does not report causal beliefs |
| Grace & MacBride-Stewart (2007) | Does not report causal beliefs |
| Grundström et al. (2023) | Not qualitative |
| Grutman et al. (2025) | Does not report causal beliefs |
| Hearn et al. (2024) | Does not aim to investigate beliefs |
| Hirsing et al. (2025) | Does not aim to investigate beliefs |
| Hong et al. (2025) | Does not aim to investigate beliefs |
| Hudson (2024) | Does not aim to investigate beliefs |
| Ito & Pascual (2024) | Does not report causal beliefs |
| Johnson et al. (2025) | Does not aim to investigate beliefs |
| Jones et al. (2004) | Does not report causal beliefs |
| Kaler (2006) | Does not report causal beliefs |
| Kanter et al. (2017) | Does not report causal beliefs |
| Kanter et al. (2016) | Conference abstract only |
| Kumar et al. (2023) | Does not report causal beliefs |
| Laan et al. (2010) | Conference abstract only |
| Leonard Hodges et al. (2024) | Does not report causal beliefs |
| Graham Longsworth et al. (2024) | Protocol only |
| Lountzi & Durand (2024) | Does not aim to investigate beliefs |
| Maggioni (2009) | Conference abstract only |
| Majchrowski (2006) | Does not report causal beliefs |
| Manuela et al. (2023) | Does not report causal beliefs |
| Marani et al. (2024) | Conference abstract only |
| March (2024) | Does not report causal beliefs |
| Mardon et al. (2024) | Does not aim to investigate beliefs |
| McCaughey et al. (2025) | Does not aim to investigate beliefs |
| McDonnell (1998) | Does not report causal beliefs |
| Mellado et al. (2020) | Does not report causal beliefs |
| Meriwether et al. (2023) | Conference abstract only |
| Meriwether et al. (2022) | Conference abstract only |
| Merli et al. (2024) | Not qualitative |
| Montali et al. (2025) | Does not report causal beliefs |
| Munro et al. (2024) | Does not report causal beliefs |
| Nogrady (2024) | Does not report causal beliefs |
| Oesterling et al. (2024) | Conference abstract only |
| O'Hara & Roufeil (2024) | Does not aim to investigate beliefs |
| Panisch et al. (2025) | Does not aim to investigate beliefs |
| Perovic et al. (2021) | Does not report causal beliefs |
| Pickup et al. (2025) | Does not aim to investigate beliefs |
| Pithavadian, Chalmers, et al. (2024) | Does not aim to investigate beliefs |
| Pithavadian, Dune, & Chalmers (2024) | Does not aim to investigate beliefs |
| Pithavadian, Dune, Chalmers, et al. (2024) | Does not aim to investigate beliefs |
| Requadt et al. (2024) | Does not aim to investigate beliefs |
| Rude et al. (2025) | Not qualitative |
| Sayer-Jones & Sherman (2025) | Does not aim to investigate beliefs |
| Seo et al. (2025) | Not related to pelvic pain |
| Suc et al. (2024) | Not in English |
| Taylor & Ridgway (2024) | Includes secondary data |
| Temmes et al. (2025) | Does not report causal beliefs |
| Templeman et al. (2023) | Does not report causal beliefs |
| Turnbull et al. (2009) | Conference abstract only |
| Valihora (2025) | Does not report causal beliefs |
| Warren et al. (2011) | Not qualitative |
| Webber et al. (2023) | Does not aim to investigate beliefs |
| Wissing et al. (2024) | Does not report causal beliefs |
| Youngstrom et al. (2025) | Does not report causal beliefs |
| Youngstrom et al. (2022) | Conference abstract only |
| Zimmermann et al. (2010) | Not in English |
| Zugaj et al. (2024) | Does not report causal beliefs |

Adams, O. R., Gesselman, A. N., & Bennett-Brown, M. (2024). Centering Women of Color: Chronic Vulvovaginal Pain (CVVP) Communication. *SOCIAL SCIENCES-BASEL*, *13*(5). <https://doi.org/10.3390/socsci13050265>

Adams, O. R., Holder-Dixon, A. R., Campbell, J. T., Bennett-Brown, M., Moscovici, Z., & Gesselman, A. N. (2025). Medical Mistrust and Healthcare Seeking Among Women of Color with Chronic Vulvovaginal Pain. *International Journal of Behavioral Medicine*, *32*(1), 21-33. <https://doi.org/https://dx.doi.org/10.1007/s12529-023-10236-4>

Ayling, K., & Ussher, J. M. (2008). "If sex hurts, am I still a woman?" the subjective experience of vulvodynia in hetero-sexual women. *Arch Sex Behav*, *37*(2), 294-304. <https://doi.org/10.1007/s10508-007-9204-1>

Blair, K. L., Pukall, C. F., Smith, K. B., & Cappell, J. (2015). Differential associations of communication and love in heterosexual, lesbian, and bisexual women's perceptions and experiences of chronic vulvar and pelvic pain. *Journal of Sex and Marital Therapy*, *41*(5), 498-524. <https://doi.org/https://dx.doi.org/10.1080/0092623X.2014.931315>

Boge-Olsnes, C., Bech Risor, M., & Oberg, G. K. (2024). Chronic pelvic pain sufferers' experiences of Norwegian psychomotor physiotherapy: a qualitative study on an embodied approach to pain. *European Journal of Physiotherapy*, *26*(1), 2EP-11. <https://doi.org/https://dx.doi.org/10.1080/21679169.2022.2136754>

Boge-Olsnes, C. M., Risor, M. B., & Oberg, G. K. (2023). Exploring the potential of a standardized test in physiotherapy: making emotion, embodiment, and therapeutic alliance count for women with chronic pelvic pain. *Frontiers in Psychology*, *14*, 1166496. <https://doi.org/https://dx.doi.org/10.3389/fpsyg.2023.1166496>

Brauer, M., Lakeman, M., van Lunsen, R., & Laan, E. (2014). Predictors of task-persistent and fear-avoiding behaviors in women with sexual pain disorders. *Journal of Sexual Medicine*, *11*(12), 3051-3063. <https://doi.org/https://dx.doi.org/10.1111/jsm.12697>

Brown, V. L., James, A., Hunleth, J., Bradley, C. S., Farrar, J. T., Gupta, P., Lai, H. H., Lowder, J. L., Moldwin, R., & Rodriguez, L. V. (2024). Believing women: a qualitative exploration of provider disbelief and pain dismissal among women with interstitial cystitis/bladder pain syndrome from the MAPP research network. *International urogynecology journal*, *35*(1), 139-148. <https://doi.org/10.1007/s00192-023-05677-0>

Bullo, S., & Hearn, J. H. (2021). Parallel worlds and personified pain: A mixed-methods analysis of pain metaphor use by women with endometriosis. *British Journal of Health Psychology*, *26*(2), 271-288. <https://doi.org/https://dx.doi.org/10.1111/bjhp.12472>

Bullo, S., & Hearn, J. H. (2024). Visual reconstructions of endometriosis pain: An interdisciplinary visual methodology for illness representation. *British Journal of Health Psychology*, *29*(3), 676-693. <https://doi.org/https://dx.doi.org/10.1111/bjhp.12720>

Burke, E., Di Renna, T., Mustafa, N., Ginter, C., Carter, W., Corkery, C., Sheffe, S., Wilson, R., Lemos, N., & Bosma, R. (2024). Empowered management for pelvic pain: The experiences of women with persistent pelvic pain participating in an online self-directed self-management program while they wait for interprofessional care. *Women's health (London, England)*, *20*, 17455057231224960. <https://doi.org/https://dx.doi.org/10.1177/17455057231224960>

Chen, C. X., Shieh, C., Draucker, C. B., & Carpenter, J. S. (2018). Reasons women do not seek health care for dysmenorrhea. *Journal of Clinical Nursing*, *27*(1-2), e301-e308. <https://doi.org/https://dx.doi.org/10.1111/jocn.13946>

Cole, J. M., Grogan, S., & Turley, E. (2021). "The most lonely condition I can imagine": Psychosocial impacts of endometriosis on women's identity. *Feminism & Psychology*, *31*(2), 171-191. <https://doi.org/https://dx.doi.org/10.1177/0959353520930602>

Coxon, L., Lugt, C., Horne, A. W., Evans, E., Abreu-Mendes, P., Arendt-Nielsen, L., Aziz, Q., Becker, C. M., Birch, J., & Charrua, A. (2024). Symptom flares in women with chronic pelvic pain: Questionnaire study within a cohort study (translational research in pelvic pain (TRiPP)). *BJOG: An International Journal of Obstetrics and Gynaecology*, *131*(13), 1832-1840. <https://doi.org/10.1111/1471-0528.17915>

Danielsen, K. G., Kaarbo, M. B., Groven, K. S., Helgesen, A. L. O., Haugstad, G. K., & Wojniusz, S. (2024). Towards improved sexual health among women with provoked vestibulodynia: experiences from a somatocognitive therapy approach. *European Journal of Physiotherapy*, *26*(1), 51EP-61. <https://doi.org/https://dx.doi.org/10.1080/21679169.2023.2168749>

De C. Williams, A. C., Azadi, A., & McGrigor, H. (2025). Experience of endometriosis pain: A qualitative study. *Pain*. <https://doi.org/10.1097/j.pain.0000000000003763>

Denny, E., & Mann, C. H. (2008). Endometriosis and the primary care consultation. *European journal of obstetrics, gynecology, and reproductive biology*, *139*(1), 111-115. <http://ovidsp.ovid.com/ovidweb.cgi?T=JS&PAGE=reference&D=med7&NEWS=N&AN=18068292>

Dogan, J. N., Thorpe, S. Y., Malone, N., Jester, J., Stevens-Watkins, D., & Hargons, C. (2023). 'My partner will think I'm weak or overthinking my pain': how being superwoman inhibits Black women's sexual pain disclosure to their partners. *Culture, Health & Sexuality*, *25*(5), 567-581. <https://doi.org/https://dx.doi.org/10.1080/13691058.2022.2072956>

Dowding, C., Mikocka-Walus, A., Skvarc, D., O'Shea, M., Olive, L., & Evans, S. (2024). Learning to cope with the reality of endometriosis: A mixed-methods analysis of psychological therapy in women with endometriosis. *British Journal of Health Psychology*, *29*(3), 644-661. <https://doi.org/10.1111/bjhp.12718>

Dowding, C., Mikocka-Walus, A., Skvarc, D., O'Shea, M., Olive, L., & Evans, S. (2025). Sharing stories, building connections, and regaining control: a qualitative study of cognitive behavioural therapy for endometriosis and persistent pelvic pain. *Counselling Psychology Quarterly*. <https://doi.org/https://dx.doi.org/10.1080/09515070.2025.2536559>

Elcombe, S., Gath, D., & Day, A. (1997). The psychological effects of laparoscopy on women with chronic pelvic pain. *Psychological medicine*, *27*(5), 1041-1050. <http://ovidsp.ovid.com/ovidweb.cgi?T=JS&PAGE=reference&D=med4&NEWS=N&AN=9300510>

Ellis, K., Munro, D., & Wood, R. (2023). Dismissal informs the priorities of endometriosis patients in New Zealand. *Frontiers in medicine*, *10*, 1185769. <https://doi.org/https://dx.doi.org/10.3389/fmed.2023.1185769>

Ellis, K., & Wood, R. (2025). 'I Wish I Fought for Myself More Instead of Just Letting Doctors Dismiss Me': A Combined Qualitative Analysis of Four Cohorts of Aotearoa New Zealand Endometriosis Patients. *Australian & New Zealand journal of obstetrics & gynaecology*. <https://doi.org/10.1111/ajo.70038>

Emad, M. C. (2006). At WITSENDO: Communal embodiment through storytelling in women's experiences with endometriosis. *Women's Studies International Forum*, *29*(2), 184-196. <https://doi.org/https://dx.doi.org/10.1016/j.wsif.2006.03.005>

Engel, J., & Hoffmann, S. O. (1994). Cultural influences on chronic pelvic pain: A study on women in Brazil. *European Journal of Pain (London, England)*, *15*(4), 61-67. <http://ovidsp.ovid.com/ovidweb.cgi?T=JS&PAGE=reference&D=emed5&NEWS=N&AN=25014604>

Fallon, L. (2025). *Qualitatively Exploring Challenges in Treatment Decision-Making for Endometriosis* <https://doi.org/10.25949/25498366.v1>

Fernandez-Martinez, E., Abreu-Sanchez, A., Perez-Corrales, J., Ruiz-Castillo, J., Velarde-Garcia, J. F., & Palacios-Cena, D. (2020). Living with Pain and Looking for a Safe Environment: A Qualitative Study among Nursing Students with Dysmenorrhea. *International Journal of Environmental Research and Public Health*, *17*(18). <https://doi.org/https://dx.doi.org/10.3390/ijerph17186670>

Fry, R. P., Crisp, A. H., & Beard, R. W. (1991). Patients' illness models in chronic pelvic pain. *Psychotherapy and Psychosomatics*, *55*(2-4), 158-163. <http://ovidsp.ovid.com/ovidweb.cgi?T=JS&PAGE=reference&D=med3&NEWS=N&AN=1891563>

Gouesbet, S., Kvaskoff, M., Riveros, C., Diard, E., Pane, I., Goussé-Breton, Z., Valenti, M., Gabillet, M., Garoche, C., Ravaud, P., & Tran, V. T. (2023). Patients' Perspectives on How to Improve Endometriosis Care: A Large Qualitative Study Within the ComPaRe-Endometriosis e-Cohort. *JOURNAL OF WOMENS HEALTH*, *32*(4), 463-470. <https://doi.org/10.1089/jwh.2022.0323>

Grace, V. M., & MacBride-Stewart, S. (2007). "How to say it": women's descriptions of pelvic pain. *Women & Health*, *46*(4), 81-98. <http://ovidsp.ovid.com/ovidweb.cgi?T=JS&PAGE=reference&D=med6&NEWS=N&AN=18512453>

Grundström, H., Engman, L., Rimhagen, E., Söderstierna, C., & Flink, I. (2023). Experiences of communication in women with endometriosis: perceived validation and invalidation in different contexts, and associations with health-related quality of life. *Journal of Psychosomatic Obstetrics & Gynecology*, *44*(1). <https://doi.org/10.1080/0167482X.2023.2264483>

Grutman, A. J., Perelmuter, S., Perez, A., Meurer, J., Contractor, M., Mathews, E., Shearer, K., Burnett, L. A., & Uloko, M. (2025). Understanding Patient Experiences of Vulvodynia Through Reddit: Qualitative Analysis. *JMIR infodemiology*, *5*, e63072. <https://doi.org/https://dx.doi.org/10.2196/63072>

Hearn, J. H., Bryson, K., Barsauskaite, L., & Bullo, S. (2024). A COM-B and Theoretical Domains Framework Mapping of the Barriers and Facilitators to Effective Communication and Help-Seeking Among People With, or Seeking a Diagnosis Of, Endometriosis. *Journal of health communication*, *29*(3), 174-186. <https://doi.org/https://dx.doi.org/10.1080/10810730.2023.2299003>

Hirsing, N., Nestoriuc, Y., Buchweitz, O., & Meyrose, A. K. (2025). Pre-operative expectations in patients with endometriosis - a qualitative interview study. *BMC WOMENS HEALTH*, *25*(1). <https://doi.org/10.1186/s12905-025-03686-3>

Hong, M., Crone, A., Gashi, E., Pietluch, M., Reinders, M., Uchida, J., Nella, A., MacKay, C., Drodge, O., & Bosma, R. (2025). Where best practice pain care and patient expectations for care meet: Exploring patient expectations around chronic pelvic pain, physiotherapy, and the biopsychosocial model of care. *Women's health (London, England)*, *21*, 17455057251349626. <https://doi.org/https://dx.doi.org/10.1177/17455057251349626>

Hudson, B. C. (2024). Private parts: A heuristic inquiry into the relational-cultural experience of chronic pelvic pain. *Dissertation Abstracts International: Section B: The Sciences and Engineering*, *85*(2-B), No-Specified. <http://ovidsp.ovid.com/ovidweb.cgi?T=JS&PAGE=reference&D=psyc23&NEWS=N&AN=2024-12026-270https://library.unisa.edu.au/go/openurl/?url_ver=Z39.88-2004&rft_val_fmt=info:ofi/fmt:kev:mtx:journal&rfr_id=info:sid/Ovid:psyc23&rft.genre=article&rft_id=info:doi/&rf>

Ito, V., & Pascual, M. (2024). Exploring women's chronic disease experiences: A mixed-methods analysis of endometriosis narratives. *Language and Health*, *2*(1), 58-65. <https://doi.org/10.1016/j.laheal.2024.02.001>

Johnson, S., Evans, E., & Hapangama, D. K. (2025). Chronic pelvic pain treatment understanding what matters: a social media survey. *Reproduction & fertility*, *6*(1). <https://doi.org/https://dx.doi.org/10.1530/RAF-24-0038>

Jones, G., Jenkinson, C., & Kennedy, S. (2004). The impact of endometriosis upon quality of life: A qualitative analysis. *Journal of Psychosomatic Obstetrics and Gynaecology*, *25*(2), 123-133. <https://doi.org/https://dx.doi.org/10.1080/01674820400002279>

Kaler, A. (2006). Unreal women: Sex, gender, identity and the lived experience of vulvar pain. *Fem Rev*(82), 49-74. <https://doi.org/10.1057/palgrave.fr.9400262>

Kanter, G., Volpe, K., Dunivan, G. C., Cichowski, S., Rogers, R. G., Jeppson, P. C., & Komesu, Y. M. (2016). Women speak up: How to make interstitial cystitis a little less painful, a focus group based approach. *Female Pelvic Medicine & Reconstructive Surgery*, *22*(5), S131. <https://doi.org/https://dx.doi.org/10.1097/SPV.0000000000000332>

Kanter, G., Volpe, K. A., Dunivan, G. C., Cichowski, S. B., Jeppson, P. C., Rogers, R. G., & Komesu, Y. M. (2017). Important role of physicians in addressing psychological aspects of interstitial cystitis/bladder pain syndrome (IC/BPS): a qualitative analysis. *Int Urogynecol J*, *28*(2), 249-256. <https://doi.org/https://dx.doi.org/10.1007/s00192-016-3109-2>

Kumar, K., Narvekar, N. N., & Maslin, K. (2023). Self-managed dietary changes and functional gut symptoms in endometriosis: A qualitative interview study. *EUROPEAN JOURNAL OF OBSTETRICS & GYNECOLOGY AND REPRODUCTIVE BIOLOGY-X*, *19*. <https://doi.org/10.1016/j.eurox.2023.100219>

Laan, E., Brauer, M., Lakeman, M., & Van Lunsen, R. (2010). Inadequate pain behavior in women with dyspareunia and primary vaginismus. *Journal of Sexual Medicine*, *7*(3), 144. <https://doi.org/https://dx.doi.org/10.1111/j.1743-6109.2010.01828-4.x>

Leonard Hodges, A. L. (2024). Investigating the experiences of evangelical couples coping with painful intercourse during early marriage. *Dissertation Abstracts International: Section B: The Sciences and Engineering*, *85*(11-B), No-Specified. <http://ovidsp.ovid.com/ovidweb.cgi?T=JS&PAGE=reference&D=psyc23&NEWS=N&AN=2024-95395-008https://library.unisa.edu.au/go/openurl/?url_ver=Z39.88-2004&rft_val_fmt=info:ofi/fmt:kev:mtx:journal&rfr_id=info:sid/Ovid:psyc23&rft.genre=article&rft_id=info:doi/&rf>

Lountzi, A. Z., & Durand, H. (2024). Help-seeking experiences and intimate partner support in vulvodynia: A qualitative exploration. *Women's health (London, England)*, *20*, 17455057241241866. <https://doi.org/https://dx.doi.org/10.1177/17455057241241866>

Maggioni, C. (2009). Body image changes in different gynaecologic disorders. *Int J Gynecol Obstet*, *107*(2), S50-S51. <https://doi.org/https://dx.doi.org/10.1016/S0020-7292%2809%2960203-4>

Majchrowski, M. H. (2006). *Experience, coping, and meaning in endometriosis: A biopsychosocial model* (Publication Number 12-B) [Doctoral thesis, Alliant International University]. ProQuest Dissertations and Theses Global. <http://ovidsp.ovid.com/ovidweb.cgi?T=JS&PAGE=reference&D=psyc6&NEWS=N&AN=2006-99012-145>

Manuela, G., Rene, W., Ines, R., & Margret, J. (2023). "I think to myself 'why now?'" - a qualitative study about endometriosis and pain in Austria. *BMC WOMENS HEALTH*, *23*(1). <https://doi.org/10.1186/s12905-023-02576-w>

Marani, S., Ahmad, M., Kingsley, B., Reyes, L., & Poirier, A. (2024). HYPNOTHERAPY FOR CHRONIC PELVIC PAIN: PATIENT & HEALTHCARE PROVIDER PERSPECTIVES. *Urogynecology*, *30*(10 Supplement), S141. <http://ovidsp.ovid.com/ovidweb.cgi?T=JS&PAGE=reference&D=emed26&NEWS=N&AN=645804102https://library.unisa.edu.au/go/openurl/?url_ver=Z39.88-2004&rft_val_fmt=info:ofi/fmt:kev:mtx:journal&rfr_id=info:sid/Ovid:emed26&rft.genre=article&rft_id=info:doi/&rft_id=>

March, K. (2024). Nether Space(s): Unveiling endometriosis experiences in performance. *Choreographic Practices*, *15*(1), 61-87. <https://doi.org/10.1386/chor_00070_1>

Mardon, A. K., Chalmers, K. J., Heathcote, L. C., Curtis, L.-A., Freedman, L., Malani, R., Parker, R., Neumann, P. B., Moseley, G. L., & Leake, H. B. (2024). "I wish I knew then what I know now" - pain science education concepts important for female persistent pelvic pain: a reflexive thematic analysis. *Pain*, *165*(9), 1990-2001. <https://doi.org/https://dx.doi.org/10.1097/j.pain.0000000000003205>

McCaughey, T., Younes, M. M., Raoofi, M., Hicks, L., Amir, M., Reddington, C., Cheng, C., Healey, M., & Peate, M. (2025). Beyond pathology: Patient experiences of laparoscopy for persistent pelvic pain with no identifiable cause found. *The Australian & New Zealand journal of obstetrics & gynaecology*, *65*(3), 351-356. <https://doi.org/https://dx.doi.org/10.1111/ajo.13905>

McDonnell, K. A. (1998). *Psychosocial adjustment among women with chronic pelvic pain: Application of a general model* (Publication Number 5-B) [Doctoral thesis, Johns Hopkins University]. ProQuest Dissertations and Theses Global. <http://ovidsp.ovid.com/ovidweb.cgi?T=JS&PAGE=reference&D=psyc3&NEWS=N&AN=1998-95022-119>

Mellado, B. H., Pilger, T. L., Poli Neto, O. B., Rosa E Silva, J. C., Nogueira, A. A., do Vale Brandao, C., & Candido Dos Reis, F. J. (2020). Daily life attitudes of women with moderate or severe chronic pelvic pain. A qualitative study. *European journal of obstetrics, gynecology, and reproductive biology*, *254*, 109-113. <https://doi.org/https://dx.doi.org/10.1016/j.ejogrb.2020.09.001>

Meriwether, K. V., Griego, J. L., Jansen, S. M., Abudushalamu, F., Petersen, T. R., Dunivan, G. C., Komesu, Y. M., & Page-Reeves, J. (2023). Beliefs and Narratives Associated with the Treatment of Chronic Pelvic Pain in Women. *Journal of Minimally Invasive Gynecology*, *30*(3), 216-229. <https://doi.org/https://dx.doi.org/10.1016/j.jmig.2022.12.002>

Meriwether, K. V., Jansen, S. M., Abudushalamu, F., Griego, J. L., Dunivan, G., Komesu, Y., & Page-Reeves, J. M. (2022). Beliefs and narratives associated with the treatment of chronic pelvic pain in women. *American Journal of Obstetrics and Gynecology*, *226*(3), S1298-S1299. <https://doi.org/https://dx.doi.org/10.1016/j.ajog.2021.12.051>

Merli, C. E. M., Cetera, G. E., Caia, C., Facchin, F., & Vercellini, P. (2024). "The sound of silence" Giving voice to endometriosis-related positional dyspareunia. *Archives of gynecology and obstetrics*, *309*(3), 887-893. <https://doi.org/10.1007/s00404-023-07205-3>

Montali, L., Bernareggi, C., & Crispiatico, V. (2025). Aggravating and protective factors in patients' experiences of vulvodynia: a qualitative study with Italian women. *BMC psychology*, *13*(1), 260. <https://doi.org/https://dx.doi.org/10.1186/s40359-024-02318-z>

Munro, C. B., Flanagan, M., Moussaoui, D., Kite, L., & Grover, S. R. (2024). Missing and dismissing the impact of periods. Outcomes of focus groups of teens with period concerns. *Women's health (London, England)*, *20*, 17455057241303003. <https://doi.org/https://dx.doi.org/10.1177/17455057241303003>

Nogrady, B. (2024). How pain is misunderstood and ignored in women. *Nature*, *633*(8031), S31EP-S33. <https://doi.org/https://dx.doi.org/10.1038/d41586-024-03005-0>

O'Hara, R., & Roufeil, L. (2024). Self-Management Among People Living With Endometriosis: A Qualitative Study. *Qualitative Health Research*, 10497323241291787. <https://doi.org/https://dx.doi.org/10.1177/10497323241291787>

Oesterling, C. F., Borg, C., & De Jong, P. (2024). FACTORS INFLUENCING WOMEN'S PERSISTENCE IN SEXUAL ACTIVITY AMIDST PAINFUL EXPERIENCES. *Journal of Sexual Medicine*, *21*(Supplement 4), iv31EP-iv32. <https://doi.org/https://dx.doi.org/10.1093/jsxmed/qdae041.066>

Panisch, L. S., Jansen, S. M., Abudushalamu, F., Petersen, T. R., & Meriwether, K. V. (2025). Patient Perspectives on the Psychosocial Impact of Chronic Pelvic Pain and Implications for Integrated Behavioral Care Approaches. *The journal of behavioral health services & research*. <https://doi.org/https://dx.doi.org/10.1007/s11414-024-09926-y>

Perovic, M., Jacobson, D., Glazer, E., Pukall, C., & Einstein, G. (2021). Are you in pain if you say you are not? Accounts of pain in Somali-Canadian women with female genital cutting. *Pain*, *162*(4), 1144-1152. <https://doi.org/10.1097/j.pain.0000000000002121>

Pickup, B., Sharpe, L., & Todd, J. (2025). Development of the COMPASS model of endometriosis: A COmprehensive model of pain encompassing agency, systemic factors and sense making. *British Journal of Health Psychology*, *30*(2), e12794. <https://doi.org/https://dx.doi.org/10.1111/bjhp.12794>

Pithavadian, R., Chalmers, K., Ramanathan, V., & Dune, T. (2024). People discuss the men who can't get it up, but what about the women who can't get it in? Women's help-seeking experiences for sexual pain-penetration disorder. *SSM - Qualitative Research in Health*. <https://doi.org/10.1016/j.ssmqr.2024.100480>

Pithavadian, R., Dune, T., & Chalmers, K. (2024). Patients' recommendations to improve help-seeking for vaginismus: a qualitative study. *BMC WOMENS HEALTH*, *24*(1), 203. <https://doi.org/10.1186/s12905-024-03026-x>

Pithavadian, R., Dune, T., Chalmers, K., & Ramanathan, V. (2024). The interrelationship between women's help-seeking experiences for vaginismus and their sense of self: a qualitative study and abductive analysis. *Health Psychology and Behavioral Medicine*. <https://doi.org/10.1080/21642850.2024.2396134>

Requadt, E., Nahlik, A. J., Jacobsen, A., & Ross, W. T. (2024). Patient experiences of endometriosis diagnosis: A mixed methods approach. *BJOG : an international journal of obstetrics and gynaecology*, *131*(7), 941-951. <https://doi.org/https://dx.doi.org/10.1111/1471-0528.17719>

Rude, T., Sevilla, C., Chen, N., Marin, P., Chavez, A., Yosufi, N., Unger, J. B., Baezconde-Garbanati, L., Dancz, C. E., Ginsberg, D., Rodriguez, L. V., & Stern, M. C. (2025). Self-identification of Pelvic Floor Disorder Symptoms Among Latina Women: The Roles of Knowledge, Attitude, Behaviors, Beliefs, and Psychosocial Factors. *Journal of Racial and Ethnic Health Disparities*. <https://doi.org/10.1007/s40615-025-02421-w>

Sayer-Jones, K., & Sherman, K. A. (2025). “My body…tends to betray me sometimes”:a qualitative analysis of affective and perceptual body image in individuals living with endometriosis. <https://researchers.mq.edu.au/en/publications/7ad0ff58-3696-41e1-8288-1ed65e50cf6e>

SE G Longsworth, B. B. D. M., Camille Boyer, Max Cressent, Katia Gheraieb. (2024). *New-onset Chronic Pelvic Pain in Transgender People Using Testosterone Therapy: an Exploratory Qualitative Study* <https://clinicaltrials.gov/study/NCT06487754>

Seo, H., Burkett, K. M., Okocha, M., Ha, H., Chaif, R., Izhar, N., Coelho, M. B., Jona, B., & Iqbal, A. (2025). Social media activism and women's health: Endometriosis awareness and support. *DIGITAL HEALTH*, *11*. <https://doi.org/10.1177/20552076251314905>

Suc, A., Stocker, A., Leroy, E., Garczynski, C., Cartault, A., & Troupel, O. (2024). Listening to teenagers suffering from severe dysmenorrhoea: DEMETER, an innovative qualitative study to provide them with better support. *Douleurs*, *25*(4), 202EP-211. <https://doi.org/https://dx.doi.org/10.1016/j.douler.2024.06.003>

Taylor, J., & Ridgway, A. (2024). When Descriptor is Diagnosis: An Autoethnographic Response to the Medical Treatment of Women with Vulvodynia. *AUSTRALIAN FEMINIST STUDIES*. <https://doi.org/10.1080/08164649.2024.2333051>

Temmes, M., Helosvuori, E., & Oikkonen, V. (2025). Chronic pain across clinical settings: the changing understanding of pain and its treatment in endometriosis. *BIOSOCIETIES*. <https://doi.org/10.1057/s41292-025-00361-6>

Templeman, L., Eberhardt, J., & Ling, J. (2023). Exploring the health care experiences of women diagnosed with vulvodynia [Physical & Somatic Disorders 3290

Promotion & Maintenance of Health & Wellness 3365]. *Journal of Sexual Medicine*, *20*(1), 97-106. <https://doi.org/https://dx.doi.org/10.1093/jsxmed/qdac023>

Turnbull, H., Mukhopadhyay, S., & Morris, E. (2009). The effect of endometriosis on quality of life in patients with a diagnosis in their younger reproductive years. *Int J Gynecol Obstet*, *107*(2), S631. <https://doi.org/https://dx.doi.org/10.1016/S0020-7292%2809%2962259-1>

Valihora, H. P. (2025). Experiences of disempowerment amongst endometriosis patients: Toward comprehensive care to address the psychosocial impact of chronic illness. *Journal of health psychology*. <https://doi.org/10.1177/13591053251331279>

Warren, J. W., Diggs, C., Horne, L., & Greenberg, P. (2011). Interstitial cystitis/painful bladder syndrome: what do patients mean by "perceived" bladder pain? *Urology*, *77*(2), 309-312. <https://doi.org/https://dx.doi.org/10.1016/j.urology.2010.08.030>

Webber, V., Miller, M., Gustafson, D., & Bajzak, K. (2023). VULVODYNIA VIEWED FROM A DISEASE PREVENTION FRAMEWORK: INSIGHTS FROM PATIENT PERSPECTIVES. *Journal of Sexual Medicine*, *20*(3), ii24. <https://doi.org/https://dx.doi.org/10.1093/jsxmed/qdad068.046>

Wissing, M. C., van der Net, L., Engels, Y., Vissers, K. C. P., Kluivers, K. B., van der Wal, S., & Notten, K. J. B. (2024). Lessons learned from the experiences of patients with chronic pelvic pain syndrome (CPP syndrome) with a multidisciplinary consultation: a qualitative study. *Regional Anesthesia and Pain Medicine*. <https://doi.org/10.1136/rapm-2024-105548>

Youngstrom, M., Buchanan, M., Sanchez-Medina, M., Voltaire, C., Collins, E., Bednarczyk, R., Northington, G., & Flowers, L. (2025). Beyond the Binary: Pelvic Pain and Pelvic Floor Disorders in Transmasculine Patients. *Urogynecology (Philadelphia, Pa.)*, *31*(7), 675-681. <https://doi.org/https://dx.doi.org/10.1097/SPV.0000000000001534>

Youngstrom, M., Voltaire, C., Harroche, J., Ujunwa, K., Flowers, L., & Northington, G. (2022). Examining pelvic floor disorders and pelvic pain in transgender persons: a piolot study. *Female Pelvic Medicine & Reconstructive Surgery*, *28*(6), S28. <https://doi.org/https://dx.doi.org/10.1097/SPV.0000000000001202>

Zimmermann, A., Brandes, I., & Babitsch, B. (2010). Information Needs of Women with Endometriosis within the Scope of Healthcare Provision. *Geburtshilfe und Frauenheilkunde*, *70*(7), 568-573. <https://doi.org/10.1055/s-0030-1250114>

Zugaj, M. R., Germeyer, A., Kranz, K., Zueger, A., & Kessler, J. (2024). Experiences of patients with endometriosis with a digital health application: a qualitative analysis. *Archives of gynecology and obstetrics*, *310*(4), 2253-2263. <https://doi.org/10.1007/s00404-024-07651-7>

# Tables S2. Characteristics of included studies

| **PUBLICATION SPECIFIC DATA** | **Ballard et al. (2006)** |
| --- | --- |
| **TITLE OF PAPER** | What’s the delay? A qualitative study of women’s experiences of reaching a diagnosis of endometriosis |
| **COUNTRY/REGION OF ORIGIN** | UK |
| **STUDY AIM** | To investigate the reasons women, experience delays in the diagnosis of endometriosis and the impact of this |
| **STUDY DESIGN** | Qualitative |
| **INTERVENTION** | NA |
| **INCLUSION CRITERIA** | Women referred to a hospital pelvic pain clinic from May 2004 - April 2005 with either a suspected or confirmed diagnosis of endometriosis |
| **RECRUITMENT STRATEGY** | 32 women referred to a hospital pelvic pain clinic from May 2004 to April 2005, with either a suspected or confirmed diagnosis of endometriosis, were recruited, convenience sampling |
| **DATA COLLECTION METHOD** | Semi structured interviews with individual women |
| **THEORETICAL FRAMEWORK** | Not reported |
| **DATA ANALYSIS METHODOLOGY** | Thematic approach (inductive from first 4 interviews and then applied to remaining) |
| **FUNDING** | Not reported |
| **CONFLICT OF INTEREST** | Not reported |
| **ETHICS APPROVAL** | Local Research Ethics and Research Governance Committees (approval number PORO 5-02) |
| **PRE-REGISTERED PROTOCOL IDENTIFICATION** | Not reported |

| **POPULATION CHARACTERISTICS** | **Ballard et al. (2006)** |
| --- | --- |
| **SAMPLE SIZE** | 32 recruited, 28 included in analysis |
| **AGE** | 16-47 (median 32 years, IQR 28–36 years) |
| **GENDER** | Female |
| **ETHNICITY** | Not reported |
| **EMPLOYMENT** | Not reported |
| **EDUCATION** | Not reported |
| **RELATIONSHIP STATUS** | Not reported |
| **SEXUALITY** | Not reported |
| **CHILDEREN** | Not reported |
| **DIAGNOSES** | Endometriosis |
| **DIAGNOSIS METHOD** | Not reported |
| **DURATION SINCE DIAGNOSIS** | Not reported |
| **DURATION OF ASSOCIATED SYMPTOMS** | 24-408 months (median: 180m, IQR: 96-234m) |
| **COMORBIDITIES** | Not reported |

| **PUBLICATION**  **SPECIFIC DATA** | **Boge-Olsenes et al. (2023)** |
| --- | --- |
| **TITLE OF PAPER** | How life events are perceived to link to bodily distress: A qualitative study of women with chronic pelvic pain |
| **COUNTRY/REGION OF ORIGIN** | Norway |
| **STUDY AIM** | The research question we address in this paper is how the women in our study perceived their complex symptoms and bodily distress in relation to their life experiences |
| **STUDY DESIGN** | Qualitative |
| **INTERVENTION** | Norwegian Psychomotor Physiotherapy |
| **INCLUSION CRITERIA** | Adult, female patients with CPP symptoms |
| **RECRUITMENT STRATEGY** | A request was addressed to the professional group of NPMP therapists in Norway – at their annual conference – to invite patients to participate in our study - Convenience sampling |
| **DATA COLLECTION METHOD** | Semi-structured interviews combined with video recordings |
| **THEORETICAL FRAMEWORK** | Phenomenology |
| **DATA ANALYSIS METHODOLOGY** | The five step phenomenological “discovery-oriented” approach of Giorgi (Giorgi, 1997; Giorgi, 1975) and Brinkmann and Kvale (2015) |
| **FUNDING** | The author(s) reported there is no funding associated with the work featured in this article |
| **CONFLICT OF INTEREST** | Not reported |
| **ETHICS APPROVAL** | We received approval of the study from the Regional Ethical Committee under the Norwegian Health Research Act in January 2019 (REK-Nord 2018/2533), and from the Norwegian Center for Research Data regarding personal data protection |
| **PRE-REGISTERED PROTOCOL IDENTIFICATION** | Not reported |

| **POPULATION CHARACTERISTICS** | **Boge-Olsenes et al. (2023)** |
| --- | --- |
| **SAMPLE SIZE** | 8 |
| **AGE** | The average age was 30 (range: 19–56) |
| **GENDER** | 7 Female, 1 Agender |
| **ETHNICITY** | Not reported, the participants were all from urban areas in northern (6), central and southern (2) Norway |
| **EMPLOYMENT** | Two were students, three were working and three were on long-term sick leave |
| **EDUCATION** | Not reported |
| **RELATIONSHIP STATUS** | Three were married or cohabiting, three had partners and two were single |
| **SEXUALITY** | All were heterosexual except one who did not specify sexuality |
| **CHILDEREN** | Two participants had children |
| **DIAGNOSES** | Chronic pelvic pain treated with Norwegian psychomotor physiotherapy. Only two had received a diagnosis relevant to the pain in the pelvic area, in one case endometriosis, and irritable bowel syndrome in the other |
| **DIAGNOSIS METHOD** | Not reported |
| **DURATION SINCE DIAGNOSIS** | Not reported |
| **DURATION OF ASSOCIATED SYMPTOMS** | Not reported |
| **COMORBIDITIES** | Two of them had other diagnoses, namely epilepsy, psychogenic non-epileptic seizures, and weakened immune system. They all suffered muscular pain from many parts of the body, and for some of them exhaustion, anxiety and depression were their main problems |

| **PUBLICATION SPECIFIC DATA** | **Boge-Olsnes et al. (2022)** |
| --- | --- |
| **TITLE OF PAPER** | Chronic pelvic pain sufferers’ experiences of Norwegian psychomotor physiotherapy: a qualitative study on an embodied approach to pain |
| **COUNTRY/REGION OF ORIGIN** | Norway |
| **STUDY AIM** | The aim of this study was to investigate women with CPP experiences of Norwegian psychomotor physiotherapy (NPMP) as an embodied approach. An increased understanding of how NPMP is experienced by women with CPP may enhance our understanding of CPP and inform physiotherapy for patients with CPP |
| **STUDY DESIGN** | Qualitative |
| **INTERVENTION** | Norwegian psychomotor physiotherapy |
| **INCLUSION CRITERIA** | Adult, female patients with CPP symptoms |
| **RECRUITMENT STRATEGY** | A request was addressed to the professional group of NPMP therapists in Norway – at their annual conference – to invite patients to participate in our study - Convenience sampling |
| **DATA COLLECTION METHOD** | Individual semi-structured interviews |
| **THEORETICAL FRAMEWORK** | Phenomenology |
| **DATA ANALYSIS METHODOLOGY** | Phenomenological method described by Høffding and Martiny |
| **FUNDING** | The author(s) reported there is no funding associated with the work featured in this article |
| **CONFLICT OF INTEREST** | The author(s) reported no potential conflict of interest |
| **ETHICS APPROVAL** | The Regional Ethical Committee approved the study in January 2019 (REK-Nord 2018/2533), and the study received approval from the Norwegian Centre for Research Data regarding personal data protection. Written informed consent was obtained from all participants upon inclusion in the study |
| **PRE-REGISTERED PROTOCOL IDENTIFICATION** | Not reported |

| **POPULATION CHARACTERISTICS** | **Boge-Olsnes et al. (2022)** |
| --- | --- |
| **SAMPLE SIZE** | 8 |
| **AGE** | The average age was 30 (range: 19–56) |
| **GENDER** | 7 Female, 1 Agender |
| **ETHNICITY** | Not reported, the participants were all from urban areas in northern, central and southern Norway |
| **EMPLOYMENT** | Two were students, three were working and three were on long-term sick leave |
| **EDUCATION** | Not reported |
| **RELATIONSHIP STATUS** | Three were married or cohabiting, three had partners and two were single |
| **SEXUALITY** | All were heterosexual except one who did not specify sexuality |
| **CHILDEREN** | Two participants had children |
| **DIAGNOSES** | Chronic pelvic pain treated with Norwegian psychomotor physiotherapy. Only two had received a diagnosis relevant to the pain in the pelvic area, in one case endometriosis, and irritable bowel syndrome in the other |
| **DIAGNOSIS METHOD** | Not reported |
| **DURATION SINCE DIAGNOSIS** | Not reported |
| **DURATION OF ASSOCIATED SYMPTOMS** | Not reported |
| **COMORBIDITIES** | One was diagnosed with epilepsy and another with psychogenic non-epileptic seizures (PNES) and weakened immune system |

| **PUBLICATION SPECIFIC DATA** | **Burbeck & Willig (2014)** |
| --- | --- |
| **TITLE OF PAPER** | The personal experience of dysmenorrhoea: An interpretative phenomenological analysis |
| **COUNTRY/REGION OF ORIGIN** | UK |
| **STUDY AIM** | To uncover the experience of dysmenorrhoea from the point of view of sufferers using interpretative phenomenological analysis |
| **STUDY DESIGN** | Qualitative |
| **INTERVENTION** | NA |
| **INCLUSION CRITERIA** | Female, regular menstrual cycle (a period every 21–33 days), experienced pain, which, so far as they were aware, did not result from organic pathology, and which interfered with everyday activity at least one cycle in three. they were required not to have been receiving medical treatment for dysmenorrhoea for more than three cycles at the time of interview, nor to be using oral contraceptives since these are used to treat dysmenorrhoea |
| **RECRUITMENT STRATEGY** | A snowball technique was utilised via the lead researcher’s contacts and the interviewees themselves |
| **DATA COLLECTION METHOD** | Semi structured interviews |
| **THEORETICAL FRAMEWORK** | Interpretative phenomenological analysis |
| **DATA ANALYSIS METHODOLOGY** | IPA |
| **FUNDING** | This research received no specific grant from any funding agency in the public, commercial, or not for profit sectors |
| **CONFLICT OF INTEREST** | Not reported |
| **ETHICS APPROVAL** | Not reported |
| **PRE-REGISTERED PROTOCOL IDENTIFICATION** | Not reported |

| **POPULATION CHARACTERISTICS** | **Burbeck & Willig  (2014)** |
| --- | --- |
| **SAMPLE SIZE** | 7 recruited, 6 included in analysis |
| **AGE** | 24-36y (mean 29y) n=7 |
| **GENDER** | Female |
| **ETHNICITY** | "White" (further details on ethnicity were not recorded) |
| **EMPLOYMENT** | Not reported |
| **EDUCATION** | Not reported |
| **RELATIONSHIP STATUS** | Not reported |
| **SEXUALITY** | Not reported |
| **CHILDEREN** | Not reported |
| **DIAGNOSES** | Painful periods |
| **DIAGNOSIS METHOD** | Not reported |
| **DURATION SINCE DIAGNOSIS** | Not reported |
| **DURATION OF ASSOCIATED SYMPTOMS** | Not reported |
| **COMORBIDITIES** | Nil diagnosed |

| **PUBLICATION SPECIFIC DATA** | **Chen et al. (2018)** |
| --- | --- |
| **TITLE OF PAPER** | What women say about their dysmenorrhea: a qualitative thematic analysis |
| **COUNTRY/REGION OF ORIGIN** | USA |
| **STUDY AIM** | Describe women’s salient thoughts about their experiences of dysmenorrhea |
| **STUDY DESIGN** | Cross-sectional qualitative |
| **INTERVENTION** | NA |
| **INCLUSION CRITERIA** | Women were eligible if they were over 18 years old, self-identified as having experienced dysmenorrhea symptoms in the last 6 months, were able to read and write in English, and were living in the United States |
| **RECRUITMENT STRATEGY** | Participants recruited from a list of survey panel registrants (Qualtrics, Provo, UT) who were willing to be contacted for surveys |
| **DATA COLLECTION METHOD** | Cross-sectional open ended online survey |
| **THEORETICAL FRAMEWORK** | Not reported |
| **DATA ANALYSIS METHODOLOGY** | Thematic analysis |
| **FUNDING** | During preparation of this manuscript, the first author was supported by Grant Number 5T32 NR007066 from the National Institute of Nursing Research of the National Institutes of Health and the Enhanced Mentoring Program with Opportunities for Ways to Excel in Research (EMPOWER) grant from Indiana University–Purdue University Indianapolis. The parent study was supported by the Eckburg Fund from University of Wisconsin-Madison. The funding agencies did not participate in the design of the study and collection, analysis, and interpretation of data, and writing the manuscript. The content is solely the responsibility of the authors and does not necessarily represent the official views of the National Institutes of Health |
| **CONFLICT OF INTEREST** | The authors declare that they have no competing interests |
| **ETHICS APPROVAL** | The study did not involve identifiable information from the participants. The parent study was reviewed by the University of Wisconsin-Madison Health Sciences Minimal Risk Institutional Review Board (Protocol Number 2014-1326), and the Institutional Review Board determined that the parent study was qualified for exemption. An information page was provided to potential participants with key elements of informed consent, and participants were informed that completion of the survey implied consent to participate |
| **PRE-REGISTERED PROTOCOL IDENTIFICATION** | Not reported |

| **POPULATION CHARACTERISTICS** | **Chen et al. (2018)** |
| --- | --- |
| **SAMPLE SIZE** | 225 |
| **AGE** | Mean age of 34.7 years (SD = 6.8, Range = 18 to 57) |
| **GENDER** | Female |
| **ETHNICITY** | Most were White/Caucasian (73.8%), and 93.3% grew up in the United States. 11.6% Hispanic, 5.3% Asian/Pacific Islander, 12.9% Black/African American, 3.1% Native American |
| **EMPLOYMENT** | Not reported |
| **EDUCATION** | About one third had a bachelor’s degree or above (36.4%), and the majority had insurance (86.7%) |
| **RELATIONSHIP STATUS** | Not reported |
| **SEXUALITY** | Not reported |
| **CHILDEREN** | Not reported |
| **DIAGNOSES** | About one-quarter (27.8%) reported having been diagnosed with conditions related to secondary dysmenorrhea. 9.8%v uterine fibroids, 5.3% endometriosis, 4.9% pelvic inflammatory disease, 3.6% other |
| **DIAGNOSIS METHOD** | Not reported |
| **DURATION SINCE DIAGNOSIS** | Not reported |
| **DURATION OF ASSOCIATED SYMPTOMS** | Not reported |
| **COMORBIDITIES** | Not reported |

| **PUBLICATION SPECIFIC DATA** | **Crouch et al. (2024)** |
| --- | --- |
| **TITLE OF PAPER** | I am in Charge, But Not Always in Control A Qualitative Exploration of Female’s Beliefs and Knowledge About Their Pelvic Pain |
| **COUNTRY/REGION OF ORIGIN** | Australia |
| **STUDY AIM** | This study aimed to explore the beliefs and knowledge that females hold about their pelvic pain through the lens of the CSM |
| **STUDY DESIGN** | Qualitative |
| **INTERVENTION** | NA |
| **INCLUSION CRITERIA** | Participants eligible were those assigned female at birth, aged 18 years or older, having pelvic pain lasting ≥6 months, having the ability to communicate in English, and having access to the internet and Zoom video conferencing software |
| **RECRUITMENT STRATEGY** | Participants were recruited by purposive sampling using the criterion-i strategy to allow for a diverse range of perspectives to be captured, with attempts made to account for age, level of education, socioeconomic status, employment, relationship status, and diagnosis |
| **DATA COLLECTION METHOD** | Semi structured, individual, video-recorded interviews were performed to allow for rapport-building and privacy, which is desirable when discussing potentially sensitive topics |
| **THEORETICAL FRAMEWORK** | Interpretive Description framework, interpretive naturalistic |
| **DATA ANALYSIS METHODOLOGY** | Interpretive Description, As such, the analysis drew on clinical understandings of pelvic pain in relation to the CSM to construct themes from the data, with an overarching goal of informing clinical practice |
| **FUNDING** | Not reported |
| **CONFLICT OF INTEREST** | K.J.C. is supported by a Leadership Investigator grant to Professor Lorimer Moseley from the National Health & Medical Research Council of Australia (ID 1178444) and has received payment for presentations given on pelvic health. She is supported by a grant awarded from The Hospital Research Fund (Grant No. PG105576). Professional bodies have reimbursed her for travel costs related to presentation of research on pelvic pain at scientific conferences. A.K.M. is supported by a research grant from the Wilson Foundation. Also, received speaker fees for lectures on pelvic pain. The remaining authors declare no conflict of interest |
| **ETHICS APPROVAL** | The study was approved by the University of Adelaide Human Research Ethics Committee (Ethics approval no: H-2023-121) |
| **PRE-REGISTERED PROTOCOL IDENTIFICATION** | A protocol was prospectively registered on Open Science Framework (<https://osf.io/jnf75/>) on July 18, 2023 |

| **POPULATION CHARACTERISTICS** | **Crouch et al. (2024)** |
| --- | --- |
| **SAMPLE SIZE** | The eligibility survey yielded 83 responses, with 34 being eligible for an interview. Of those eligible to participate, 22 were invited for an interview. Twelve people participated in an interview and 10 participants did not continue to the interview |
| **AGE** | 23-42 |
| **GENDER** | Female |
| **ETHNICITY** | Not reported |
| **EMPLOYMENT** | Employed: 3 <10 hrs/pw, 3 10-25, |
| **EDUCATION** | Diploma/certificate: 2  Bachelor's degree: 8  Masters/PhD: 1 Completed high school: 1 |
| **RELATIONSHIP STATUS** | De facto/married: 3  In a relationship: 6 Single: 3 |
| **SEXUALITY** | Not reported |
| **CHILDEREN** | Not reported |
| **DIAGNOSES** | Endometriosis: 10 Dysmenorrhea: 6  Vulvodynia: 2 Vaginismus: 2 Adenomyosis: 1 Prolapsed fibroids: 1 Not diagnosed: 1 |
| **DIAGNOSIS METHOD** | GP: 2 OBGYN: 10 Physio: 1 Not diagnosed: 1 |
| **DURATION SINCE DIAGNOSIS** | Not reported |
| **DURATION OF ASSOCIATED SYMPTOMS** | 2->10 yrs |
| **COMORBIDITIES** | Not reported |

| **PUBLICATION SPECIFIC DATA** | **Danielsen et al. (2019)** |
| --- | --- |
| **TITLE OF PAPER** | Recovering from provoked vestibulodynia: Experiences from encounters with somatocognitive therapy |
| **COUNTRY/REGION OF ORIGIN** | Norway |
| **STUDY AIM** | The aim of this paper is to enhance our knowledge of the way that the therapeutic process is experienced by women with PVD undergoing somatocognitive therapy (SCT) |
| **STUDY DESIGN** | Qualitative |
| **INTERVENTION** | Somatocognitive therapy |
| **INCLUSION CRITERIA** | Women diagnosed with PVD |
| **RECRUITMENT STRATEGY** | The participants were recruited into this study from an outpatient clinic based on information about the study provided by the outpatient clinic. After treatment, treating student physios were contacted by the interviewer and assisted in setting up a meeting with their respective patients at the clinic. |
| **DATA COLLECTION METHOD** | Interviews |
| **THEORETICAL FRAMEWORK** | Not reported |
| **DATA ANALYSIS METHODOLOGY** | Thematic analysis |
| **FUNDING** | Not reported |
| **CONFLICT OF INTEREST** | The authors report no conflicts of interest. |
| **ETHICS APPROVAL** | The study, which went through a human subject review process, was approved by both the Norwegian Social Sciences Data Services and the Regional Committee for Medical and Health Research Ethics |
| **PRE-REGISTERED PROTOCOL IDENTIFICATION** | Not reported |

| **POPULATION CHARACTERISTICS** | **Danielsen et al. (2019)** |
| --- | --- |
| **SAMPLE SIZE** | 6 |
| **AGE** | Between the ages of 22 and 37, with a mean age of 29 |
| **GENDER** | Female |
| **ETHNICITY** | Scandinavian |
| **EMPLOYMENT** | Assistant job =1 Admin. job =3 Student =2 |
| **EDUCATION** | Most of the women had or were in the process of getting higher education |
| **RELATIONSHIP STATUS** | Five of the women were in a committed relationship, while one referred to herself as single |
| **SEXUALITY** | All six women identified themselves as heterosexual |
| **CHILDEREN** | None of the participants had any children |
| **DIAGNOSES** | Provoked vestibulodynia |
| **DIAGNOSIS METHOD** | All women were diagnosed with PVD by the same gynaecologist working at a University Hospital. |
| **DURATION SINCE DIAGNOSIS** | Not reported |
| **DURATION OF ASSOCIATED SYMPTOMS** | Most had lived with PVD for several years with the duration of pain ranging from just under a year to over 13 years, with an average duration of 5.5 years |
| **COMORBIDITIES** | Not reported |

| **PUBLICATION SPECIFIC DATA** | **de Jesus et al. (2024)** |
| --- | --- |
| **TITLE OF PAPER** | Chronic pelvic pain in women: the path to outpatient discharge |
| **COUNTRY/REGION OF ORIGIN** | Brazil |
| **STUDY AIM** | This study aimed to understand the meaning of CPP from the perspective of diagnosed women and analyze the determining factors for outpatient discharge |
| **STUDY DESIGN** | Qualitative |
| **INTERVENTION** | NA |
| **INCLUSION CRITERIA** | Women with CPP undergoing outpatient treatment or who had been discharged from an outpatient clinic, aged 18 years or older, who were not pregnant or had a history of pregnancy in the last 12 months, were included |
| **RECRUITMENT STRATEGY** | Randomly selected patients undergoing discharge/discharged from outpatient clinic, obtained access to records containing the name and telephone number of patients who were discharged from the outpatient clinic and those undergoing treatment |
| **DATA COLLECTION METHOD** | Interviews with a semi-structured questionnaire and field observation |
| **THEORETICAL FRAMEWORK** | Social research, strategic research, The study of CPP is directed towards social research, proposed by Minayo, as a theoretical methodological reference.(11) Among the five modalities of social research proposed by Bulmer, strategic research is more appropriate for this study, as this type of research is based on social science theories, but is oriented towards concrete, focal problems that arise in society, proving to be appropriate for knowledge and intervention of CPP(11) |
| **DATA ANALYSIS METHODOLOGY** | Data were analyzed using content analysis proposed by Bardin.(12) This technique consists of three major stages: 1) pre-analysis; 2) material exploration; 3) treatment of results, inference and interpretation. Data analysis was carried out using systematic and objective procedures to describe what was obtained in the responses to the questionnaire(12) |
| **FUNDING** | Not reported |
| **CONFLICT OF INTEREST** | Nothing to declare |
| **ETHICS APPROVAL** | The research was approved by the institution’s Research Ethics Committee, under Opinion 4.654.237 and CAAE (Certificado de Apresentação para Apreciação Ética - Certificate of Presentation for Ethical Consideration) 45022721.8.0000.5078, in compliance with Resolution 466/2012 of the Brazilian National Health Council |
| **PRE-REGISTERED PROTOCOL IDENTIFICATION** | Not reported |

| **POPULATION CHARACTERISTICS** | **de Jesus et al. (2024)** |
| --- | --- |
| **SAMPLE SIZE** | 14 |
| **AGE** | Mean age of 42.5 years |
| **GENDER** | Women |
| **ETHNICITY** | Not reported |
| **EMPLOYMENT** | Not reported |
| **EDUCATION** | On average, they studied for 10 years |
| **RELATIONSHIP STATUS** | 57.1% were married |
| **SEXUALITY** | Not reported |
| **CHILDEREN** | 85.7% had children |
| **DIAGNOSES** | CPP, no specific diagnosis reported |
| **DIAGNOSIS METHOD** | Not reported |
| **DURATION SINCE DIAGNOSIS** | Not reported |
| **DURATION OF ASSOCIATED SYMPTOMS** | On average they had felt pelvic pain for 12 years |
| **COMORBIDITIES** | Not reported |

| **PUBLICATION SPECIFIC DATA** | **Denny (2009)** |
| --- | --- |
| **TITLE OF PAPER** | “I Never Know From One Day to Another How I Will Feel”: Pain and Uncertainty in Women With Endometriosis |
| **COUNTRY/REGION OF ORIGIN** | UK |
| **STUDY AIM** | The aim of this prospective study was to explore women’s experience of living with endometriosis |
| **STUDY DESIGN** | A prospective study over a 1-year period, qualitative descriptive |
| **INTERVENTION** | NA |
| **INCLUSION CRITERIA** | Laparoscopically diagnosed endometriosis |
| **RECRUITMENT STRATEGY** | Purposive sampling from a dedicated endometriosis clinic in a specialist women’s hospital in the United Kingdom |
| **DATA COLLECTION METHOD** | Semi-structured interviews, participant diary |
| **THEORETICAL FRAMEWORK** | Storytelling approach |
| **DATA ANALYSIS METHODOLOGY** | Narrative analysis |
| **FUNDING** | Birmingham Women’s Hospital grant |
| **CONFLICT OF INTEREST** | Not reported |
| **ETHICS APPROVAL** | Ethical approval for Denny / Endometriosis and Uncertainty 987 the study was obtained from the relevant local research ethics committee of the National Health Service (NHS) |
| **PRE-REGISTERED PROTOCOL IDENTIFICATION** | Not reported |

| **POPULATION CHARACTERISTICS** | **Denny (2009)** |
| --- | --- |
| **SAMPLE SIZE** | 30 |
| **AGE** | Not reported |
| **GENDER** | Female |
| **ETHNICITY** | Twenty-seven women described their ethnicity as White British, one as Afro-Caribbean British, one as Indo Caribbean, and one as South American Indian |
| **EMPLOYMENT** | Not reported |
| **EDUCATION** | Not reported |
| **RELATIONSHIP STATUS** | Twenty women were married or cohabiting |
| **SEXUALITY** | Not reported |
| **CHILDEREN** | 11 had children, ranging in number from one to three |
| **DIAGNOSES** | Endometriosis |
| **DIAGNOSIS METHOD** | Laparoscopy |
| **DURATION SINCE DIAGNOSIS** | Not reported |
| **DURATION OF ASSOCIATED SYMPTOMS** | The average time from experiencing symptoms to diagnosis was 5.65 years, the range being from less than 1 year to 18 years |
| **COMORBIDITIES** | Not reported |

| **PUBLICATION SPECIFIC DATA** | **Dogan et al. (2022)** |
| --- | --- |
| **TITLE OF PAPER** | ‘My partner will think I’m weak or overthinking my pain’: how being superwoman inhibits Black women’s sexual pain disclosure to their partners |
| **COUNTRY/REGION OF ORIGIN** | USA |
| **STUDY AIM** | This study explored if Black women perceived their sexual pain to be an issue and what cultural, interpersonal and intrapsychic barriers hinder Black women’s disclosure of sexual pain to their partners |
| **STUDY DESIGN** | Qualitative |
| **INTERVENTION** | NA |
| **INCLUSION CRITERIA** | (a) Identifying as a Black cisgender woman, (b) being between 18-50 years of age, (c) being pre-menopausal, (d) living in a Southern US state, and (e) having previous experience of sexual intercourse |
| **RECRUITMENT STRATEGY** | Social media platforms (e.g. Instagram and Facebook) and by word-of-mouth |
| **DATA COLLECTION METHOD** | Open-ended responses from survey |
| **THEORETICAL FRAMEWORK** | sexual script theory with the Superwoman Schema |
| **DATA ANALYSIS METHODOLOGY** | Reflexive thematic analysis |
| **FUNDING** | This study was partially funded by a Center for Positive Sexuality’s Race and Sexuality grant |
| **CONFLICT OF INTEREST** | No potential conflict of interest was reported by the authors |
| **ETHICS APPROVAL** | Institutional review board approval from the University of Kentucky |
| **PRE-REGISTERED PROTOCOL IDENTIFICATION** | Not reported |

| **POPULATION CHARACTERISTICS** | **Dogan et al. (2022)** |
| --- | --- |
| **SAMPLE SIZE** | 79 - 65 completed open ended question |
| **AGE** | Between 21 and 44 years of age (M¼30.18, SD ¼ 6.01) |
| **GENDER** | Female |
| **ETHNICITY** | African American 71 (89.9) African 5 (6.3) Caribbean 6 (7.6) Latinx 5 (6.3) Biracial 3 (3.8) |
| **EMPLOYMENT** | Not reported |
| **EDUCATION** | Technical college 1 (1.3) Some college 9 (11.4) Bachelor’s degree 22 (27.8) Graduate/Professional Degree 47 (59.5) |
| **RELATIONSHIP STATUS** | Single 44 (55.7) In a relationship 13 (16.5) Cohabitating 4 (5.1) Married 9 (11.4) Divorced 3 (3.8) Dating 6 (7.6) |
| **SEXUALITY** | Heterosexual 64 (81.0) Bisexual 7 (8.9) Gay/Lesbian 2 (2.5) Queer 3 (3.8) Pansexual 3 (3.8) |
| **CHILDEREN** | Not reported |
| **DIAGNOSES** | Sexual pain |
| **DIAGNOSIS METHOD** | Self-report from survey |
| **DURATION SINCE DIAGNOSIS** | Not reported |
| **DURATION OF ASSOCIATED SYMPTOMS** | Not reported |
| **COMORBIDITIES** | Not reported |

[8]

| **PUBLICATION SPECIFIC DATA** | **Donaldson & Meana (2011)** |
| --- | --- |
| **TITLE OF PAPER** | Early Dyspareunia Experience in Young Women: Confusion, Consequences, and Help-seeking Barriers |
| **COUNTRY/REGION OF ORIGIN** | USA |
| **STUDY AIM** | To explore the subjective experience of early dyspareunia symptoms in young women so as to model its cognitive, emotional, behavioural, and help-seeking trajectory |
| **STUDY DESIGN** | Qualitative methodology broadly based on grounded theory |
| **INTERVENTION** | NA |
| **INCLUSION CRITERIA** | The Female Sexual Functioning Index (FSFI) was used as a screening tool to recruit women with sexual pain [17]. They were considered to meet inclusion criteria if they had a pain score of 3.6 or less, indicating that pain of at least a moderate intensity was experienced on at least half of sexual intercourse attempts |
| **RECRUITMENT STRATEGY** | Participants were recruited from psychology undergraduate classes and advertisements placed in the University of Nevada, Las Vegas Psychology Subject Pool Website |
| **DATA COLLECTION METHOD** | Semi-structured interviews |
| **THEORETICAL FRAMEWORK** | Broadly based on grounded theory |
| **DATA ANALYSIS METHODOLOGY** | Grounded theory |
| **FUNDING** | Not reported |
| **CONFLICT OF INTEREST** | None |
| **ETHICS APPROVAL** | This study was reviewed and approved by the Institutional Review Board of the University of Nevada, Las Vegas |
| **PRE-REGISTERED PROTOCOL IDENTIFICATION** | Not reported |

| **POPULATION CHARACTERISTICS** | **Donaldson & Meana (2011)** |
| --- | --- |
| **SAMPLE SIZE** | 14 |
| **AGE** | Av. 19.07 (SD = 1.44) |
| **GENDER** | Female |
| **ETHNICITY** | Nine were European-American, three were African-American, one was Hispanic, and one was Asian-American |
| **EMPLOYMENT** | Not reported |
| **EDUCATION** | Not reported |
| **RELATIONSHIP STATUS** | 13 either had a steady boyfriend or were actively dating, and one was cohabitating with her partner |
| **SEXUALITY** | Not reported |
| **CHILDEREN** | No children |
| **DIAGNOSES** | Sexual pain/dyspareunia |
| **DIAGNOSIS METHOD** | The Female Sexual Functioning Index (FSFI) from screening |
| **DURATION SINCE DIAGNOSIS** | Not reported |
| **DURATION OF ASSOCIATED SYMPTOMS** | Average of 15 months (SD = 14.15; range = 3–59 months) |
| **COMORBIDITIES** | Not reported |

| **PUBLICATION SPECIFIC DATA** | **Girard et al. (2023)** |
| --- | --- |
| **TITLE OF PAPER** | Women’s lived experience of endometriosis related fertility issues |
| **COUNTRY/REGION OF ORIGIN** | Switzerland |
| **STUDY AIM** | The aim of the present study is to further elaborate on the quantitative study by a qualitative investigation to provide a deeper understanding of the women’s views about endometriosis, fertility and their perception of reproductive options |
| **STUDY DESIGN** | Qualitative |
| **INTERVENTION** | NA |
| **INCLUSION CRITERIA** | Not reported |
| **RECRUITMENT STRATEGY** | Eleven participants who had responded to the online quantitative survey [13] and who had accepted to be contacted for a qualitative interview were recruited |
| **DATA COLLECTION METHOD** | Questioned using face-to-face semi-structured interviews, conducted in French |
| **THEORETICAL FRAMEWORK** | (i.e., a grounded theory, open-enquiry approach) |
| **DATA ANALYSIS METHODOLOGY** | Thematic content analysis |
| **FUNDING** | The study was funded by a grant from Geneva University Hospitals (PRD 1-2017-2) received by Dr Isabelle Navarria-Forney. The funders had no role in the study design, data collection and analysis, decision to publish or preparation of the manuscript |
| **CONFLICT OF INTEREST** | The authors have declared that no competing interests exist |
| **ETHICS APPROVAL** | Not reported |
| **PRE-REGISTERED PROTOCOL IDENTIFICATION** | Not reported |

| **POPULATION CHARACTERISTICS** | **Girard et al. (2023)** |
| --- | --- |
| **SAMPLE SIZE** | 11 |
| **AGE** | 19-39, 31.4 +/- 5.7 |
| **GENDER** | Women |
| **ETHNICITY** | Not reported |
| **EMPLOYMENT** | 1 student, all others employed:  Student  Secretary  Civil servant Teacher  Saleswoman  Project manager Nurse  Instructor Complementary medicine therapist Project manager |
| **EDUCATION** | Not reported |
| **RELATIONSHIP STATUS** | 3 single, 3 defacto, 4 married |
| **SEXUALITY** | Not reported |
| **CHILDEREN** | Not reported |
| **DIAGNOSES** | Endo |
| **DIAGNOSIS METHOD** | Not reported |
| **DURATION SINCE DIAGNOSIS** | Not reported |
| **DURATION OF ASSOCIATED SYMPTOMS** | Not reported |
| **COMORBIDITIES** | Not reported |

| **PUBLICATION SPECIFIC DATA** | **Glazer (2010)** |
| --- | --- |
| **TITLE OF PAPER** | EMBODIMENT, PAIN, AND CIRCUMCISION IN SOMALI-CANADIAN WOMEN |
| **COUNTRY/REGION OF ORIGIN** | Canada |
| **STUDY AIM** | We aimed to explore this possibly primarily through subjective reports of pain and secondarily, by objective measures of pain including hyperalgesia localized to the vulvar region, and survey reports of multiple regions of pain (in the vulvar region or globally). We were interested in discussing testable pain along with interpretation and meaning of bodily pain and FGC from the perspective of women with FGC |
| **STUDY DESIGN** | Qualitative |
| **INTERVENTION** | NA |
| **INCLUSION CRITERIA** | Somali women of reproductive age (pre-menopausal for consistent hormonal status; approximately ages 18-45) who had undergone Type III FGC in Somalia, minimum of two years living in Canada, without chronic illness |
| **RECRUITMENT STRATEGY** | Recruitment was carried out by members of the CAG comprising a convenience sample |
| **DATA COLLECTION METHOD** | Semi-structured interviews, participant diary, Additional notes came from CAG meetings, qualitative team meetings, and one knowledge translation and feedback seminar with five study participants. |
| **THEORETICAL FRAMEWORK** | Phenomenology |
| **DATA ANALYSIS METHODOLOGY** | Nomothetic analysis |
| **FUNDING** | Not reported |
| **CONFLICT OF INTEREST** | Not reported |
| **ETHICS APPROVAL** | Ethics approval for our study was obtained from the University of Toronto Research Ethics Board |
| **PRE-REGISTERED PROTOCOL IDENTIFICATION** | Not reported |

| **POPULATION CHARACTERISTICS** | **Glazer (2010)** |
| --- | --- |
| **SAMPLE SIZE** | 14 |
| **AGE** | 21 - 46, mean 38.3 |
| **GENDER** | Female |
| **ETHNICITY** | Somalian |
| **EMPLOYMENT** | Mostly working, 1 not, 4 WFH, 1 unknown |
| **EDUCATION** | Unknown, no formal education, 16 yrs of school, some high school, high school, college, university |
| **RELATIONSHIP STATUS** | Mostly married, 1 divorced, 1 unknown |
| **SEXUALITY** | Not reported |
| **CHILDEREN** | All had children |
| **DIAGNOSES** | Type III FCG |
| **DIAGNOSIS METHOD** | Not reported |
| **DURATION SINCE DIAGNOSIS** | FCG performed between ages 6-13y |
| **DURATION OF ASSOCIATED SYMPTOMS** | Not reported |
| **COMORBIDITIES** | Not reported |

| **PUBLICATION SPECIFIC DATA** | **Grace & MacBride-Stewart (2007)** |
| --- | --- |
| **TITLE OF PAPER** | ‘Women get this’: gendered meanings of chronic pelvic pain |
| **COUNTRY/REGION OF ORIGIN** | New Zealand |
| **STUDY AIM** | Aim to analyse women's reflections on ‘how come’ they have chronic pelvic pain |
| **STUDY DESIGN** | Qualitative |
| **INTERVENTION** | NA |
| **INCLUSION CRITERIA** | Women who had chronic pain in the pelvic area that was not associated with either periods or sexual activity, and also women who had chronic dysmenorrhoea, and/or chronic dyspareunia. ‘Chronic’ pain was described as pain that had been present (either continually or on and off) for at least six months. Pain was indicated as ‘moderate’ or ‘severe’ on a verbal rating question (scale of mild/moderate/severe), and/or 4.6 or higher on a 10-centimetre visual analogue scale |
| **RECRUITMENT STRATEGY** | Survey database - prevalence of chronic pelvic pain |
| **DATA COLLECTION METHOD** | Open-ended approach to interviewing |
| **THEORETICAL FRAMEWORK** | Informed by phenomenology with a biocultural approach |
| **DATA ANALYSIS METHODOLOGY** | Not reported |
| **FUNDING** | University of Canterbury funded this project through a grant and provision of a Postdoctoral Fellowship |
| **CONFLICT OF INTEREST** | Not reported |
| **ETHICS APPROVAL** | Ethical approval was obtained from the relevant regional ethics committees (seven regions in total). |
| **PRE-REGISTERED PROTOCOL IDENTIFICATION** | Not reported |

| **POPULATION CHARACTERISTICS** | **Grace & MacBride-Stewart (2007)** |
| --- | --- |
| **SAMPLE SIZE** | 40 |
| **AGE** | Between 22 and 51 years |
| **GENDER** | Female |
| **ETHNICITY** | New Zealand women of European descent. Very few women describing themselves as Māori, or Māori and another ethnicity, returned the questionnaire, and none met the criteria for inclusion. |
| **EMPLOYMENT** | Not reported |
| **EDUCATION** | Not reported |
| **RELATIONSHIP STATUS** | Not reported |
| **SEXUALITY** | Not reported |
| **CHILDEREN** | Not reported |
| **DIAGNOSES** | Thirty-three had chronic pelvic pain that was not associated with menstruation or sexual activity; 38 had dysmenorrhoea; 29 had dyspareunia; 24 had all three |
| **DIAGNOSIS METHOD** | Not reported |
| **DURATION SINCE DIAGNOSIS** | Not reported |
| **DURATION OF ASSOCIATED SYMPTOMS** | Not reported |
| **COMORBIDITIES** | Not reported |

| **PUBLICATION SPECIFIC DATA** | **Groven et al. (2016)** |
| --- | --- |
| **TITLE OF PAPER** | “Will I ever be a true woman?” An exploration of the experiences of women with vestibulodynia |
| **COUNTRY/REGION OF ORIGIN** | Norway |
| **STUDY AIM** | Explore women’s bodily experiences of vestibulodynia and how they describe its impact on their daily lives |
| **STUDY DESIGN** | Qualitative |
| **INTERVENTION** | NA |
| **INCLUSION CRITERIA** | Female, struggled with vestibulodynia for several years |
| **RECRUITMENT STRATEGY** | Recruited through two clinics offering physiotherapy treatment within the primary health care system |
| **DATA COLLECTION METHOD** | Interviews |
| **THEORETICAL FRAMEWORK** | Phenomenological approach |
| **DATA ANALYSIS METHODOLOGY** | Thematic analysis |
| **FUNDING** | Not reported |
| **CONFLICT OF INTEREST** | Not reported |
| **ETHICS APPROVAL** | This study was approved by the Norwegian Regional Committee for Medical and Health Research Ethics |
| **PRE-REGISTERED PROTOCOL IDENTIFICATION** | Not reported |

| **POPULATION CHARACTERISTICS** | **Groven et al. (2016)** |
| --- | --- |
| **SAMPLE SIZE** | 8 |
| **AGE** | Aged between 23 and 32 |
| **GENDER** | Female |
| **ETHNICITY** | Norwegian |
| **EMPLOYMENT** | NA |
| **EDUCATION** | NA |
| **RELATIONSHIP STATUS** | Six of them were in a relationship (one was married), while two described themselves as single |
| **SEXUALITY** | All identified themselves as heterosexual |
| **CHILDEREN** | None of the women had children. |
| **DIAGNOSES** | Vestibulodynia |
| **DIAGNOSIS METHOD** | Not reported |
| **DURATION SINCE DIAGNOSIS** | Not reported |
| **DURATION OF ASSOCIATED SYMPTOMS** | "Several years" |
| **COMORBIDITIES** | Not reported |

| **PUBLICATION SPECIFIC DATA** | **Hamilton (2011)** |
| --- | --- |
| **TITLE OF PAPER** | A GROUNDED THEORY OF COPING AMONG WOMEN WITH CHRONIC PELVIC PAIN |
| **COUNTRY/REGION OF ORIGIN** | USA |
| **STUDY AIM** | The primary aims of this dissertation research were to develop a theory of coping among women with CPP and to develop the groundwork of an integrated model of CPP in women |
| **STUDY DESIGN** | Nonexperimental mixed methods study design |
| **INTERVENTION** | NA |
| **INCLUSION CRITERIA** | (a) A medical diagnosis of chronic pelvic pain or a subcategory (e.g., vulvodynia); (b) length of time since diagnosis of the condition was at least 6 months; and (c) access to a private internet connection (e.g., at home in a room that could be made private) |
| **RECRUITMENT STRATEGY** | The researcher posted study announcements through online pelvic pain support groups (Facebook and several Yahoo listservs), the United Kingdom Pelvic Pain Support Network, and the National Vulvodynia Association in the United States |
| **DATA COLLECTION METHOD** | Interviews, daily tracking form of pain distress, CPP stressors, and coping efforts that included space for written comments |
| **THEORETICAL FRAMEWORK** | Grounded theory Constructionist epistemological perspective |
| **DATA ANALYSIS METHODOLOGY** | Grounded theory methodology |
| **FUNDING** | Saybrook Alumni Council awarded a scholarship that helped to complete this dissertation |
| **CONFLICT OF INTEREST** | Not reported |
| **ETHICS APPROVAL** | Not reported |
| **PRE-REGISTERED PROTOCOL IDENTIFICATION** | Not reported |

| **POPULATION CHARACTERISTICS** | **Hamilton (2011)** |
| --- | --- |
| **SAMPLE SIZE** | 15 |
| **AGE** | Avr. 31.7 years, with a range between 20 and 44 years |
| **GENDER** | Female |
| **ETHNICITY** | 73% white, 7% respectively, African American, Asian American & White, Latina, Native American & White |
| **EMPLOYMENT** | Employed outside of home 60%, Full-time student 20% Unemployed 13% Working at home 7% |
| **EDUCATION** | Some college 40%, Bachelor’s degree 20%, Some graduate school 27% Master’s degree 13% |
| **RELATIONSHIP STATUS** | Single 20%, In a relationship 27%, Married 47%, Divorced 13% |
| **SEXUALITY** | Not reported |
| **CHILDEREN** | 67% no children |
| **DIAGNOSES** | CPP Endo = 8 IC = 4 IBS = 2 Ovarian cysts = 1 PBS = 1 pelvic adhesions = 1 PF dysfunction = 3 uterine fibroids = 2 vulvar vestibulitis = 4 diastasis symphysis pubis = 1 |
| **DIAGNOSIS METHOD** | Not reported |
| **DURATION SINCE DIAGNOSIS** | Not reported |
| **DURATION OF ASSOCIATED SYMPTOMS** | 1-22 years, av. 8.47y |
| **COMORBIDITIES** | Ten of the research participants had a diagnosis of a medical condition unrelated to CPP (e.g., asthma). Six of the research participants (40% of the sample) had CPP that was comorbid with a secondary stress-related medical condition (e.g., migraines, temporomandibular joint disorder, irritable bowel syndrome, etc.) |

| **PUBLICATION SPECIFIC DATA** | **Helosvuori & Oikkonen (2024)** |
| --- | --- |
| **TITLE OF PAPER** | Sensing pain: Embodied knowledge in endometriosis |
| **COUNTRY/REGION OF ORIGIN** | Finland |
| **STUDY AIM** | We examine how our interlocutors use their embodied sensations of pain to adapt to the evolving biomedical and lived surroundings in which their pain is evaluated and managed |
| **STUDY DESIGN** | Not reported |
| **INTERVENTION** | NA |
| **INCLUSION CRITERIA** | People diagnosed with endometriosis in Finland |
| **RECRUITMENT STRATEGY** | Call posted on our project website and circulated through social media platforms, including a national patient organization’s networks |
| **DATA COLLECTION METHOD** | Semi-structured interviews |
| **THEORETICAL FRAMEWORK** | Not reported |
| **DATA ANALYSIS METHODOLOGY** | Both authors read the materials and discussed the inclusion of examples, identification of emerging themes and interpretation of quotations. In the case of differences in interpretation, we discussed the premises on which our readings of the material relied on to arrive at a shared interpretation. The analysis began with a tentative identification of broad themes, such as ‘past experiences of pain as a source of knowledge’, or ‘difficulty of fitting experiences of pain within medical frameworks’. With further rounds of reading, we conceptualized these themes as the above-described four processes of attuning to changes in pain, learning to distinguish between harmless and dangerous pain, preparing to communicate pain and negotiating the discrepancies between experiences and biomedical verification and measuring |
| **FUNDING** | The author(s) disclosed receipt of the following financial support for the research, authorship, and/or publication of this article: This research was funded by the Research Council of Finland (decision number 339143) and the Kone Foundation |
| **CONFLICT OF INTEREST** | The author(s) declared no potential conflicts of interest with respect to the research, authorship, and/or publication of this article |
| **ETHICS APPROVAL** | Not reported - We followed the ethical guidelines of the Finnish National Board on Research Integrity throughout research design, data gathering, analysis and  reporting of the results |
| **PRE-REGISTERED PROTOCOL IDENTIFICATION** | Not reported |

| **POPULATION CHARACTERISTICS** | **Helosvuori & Oikkonen (2024)** |
| --- | --- |
| **SAMPLE SIZE** | 26 |
| **AGE** | A range of ages, from the early 20s to the late 40s |
| **GENDER** | Not reported |
| **ETHNICITY** | Not reported |
| **EMPLOYMENT** | Not reported |
| **EDUCATION** | Not reported |
| **RELATIONSHIP STATUS** | Not reported |
| **SEXUALITY** | Not reported |
| **CHILDEREN** | Not reported |
| **DIAGNOSES** | Endometriosis |
| **DIAGNOSIS METHOD** | Not reported |
| **DURATION SINCE DIAGNOSIS** | Not reported |
| **DURATION OF ASSOCIATED SYMPTOMS** | Not reported |
| **COMORBIDITIES** | Not reported |

| **PUBLICATION SPECIFIC DATA** | **Huntington & Gilmour (2005)** |
| --- | --- |
| **TITLE OF PAPER** | A life shaped by pain: women and endometriosis |
| **COUNTRY/REGION OF ORIGIN** | New Zealand |
| **STUDY AIM** | The research aim was to explore women’s perceptions of living with endometriosis, its effects on their lives and the strategies used to manage their disease |
| **STUDY DESIGN** | A qualitative research design informed by feminist research principles |
| **INTERVENTION** | NA |
| **INCLUSION CRITERIA** | Female, endometriosis |
| **RECRUITMENT STRATEGY** | Presentation at a local endometriosis support groups and publication in national and local endometriosis support group newsletters |
| **DATA COLLECTION METHOD** | Semi-structured, interactive interviews |
| **THEORETICAL FRAMEWORK** | Feminist |
| **DATA ANALYSIS METHODOLOGY** | Thematic analysis |
| **FUNDING** | Not reported |
| **CONFLICT OF INTEREST** | Not reported |
| **ETHICS APPROVAL** | Regional Health Authority Ethics Committee and the institutional ethics committee |
| **PRE-REGISTERED PROTOCOL IDENTIFICATION** | Not reported |

| **POPULATION CHARACTERISTICS** | **Huntington & Gilmour (2005)** |
| --- | --- |
| **SAMPLE SIZE** | 18 |
| **AGE** | 16-45y |
| **GENDER** | Female |
| **ETHNICITY** | Not reported |
| **EMPLOYMENT** | Either currently held or had held positions of responsibility in terms of paid work |
| **EDUCATION** | Were educated at a tertiary level |
| **RELATIONSHIP STATUS** | Not reported |
| **SEXUALITY** | Not reported |
| **CHILDEREN** | Not reported |
| **DIAGNOSES** | Endometriosis |
| **DIAGNOSIS METHOD** | Not reported |
| **DURATION SINCE DIAGNOSIS** | Not reported |
| **DURATION OF ASSOCIATED SYMPTOMS** | Not reported |
| **COMORBIDITIES** | Not reported |

| **PUBLICATION SPECIFIC DATA** | **Katz et al. (2024)** |
| --- | --- |
| **TITLE OF PAPER** | ‘Listen to women as if they were your most cherished person’: Australian women’s perspectives on living with the pain of endometriosis: A mixed-methods study |
| **COUNTRY/REGION OF ORIGIN** | Australia |
| **STUDY AIM** | This study aims to use the strength of mixed-methods to investigate the relationship between women’s salient perspectives and biopsychosocial factors of fatigue, perceived social support, psychological symptoms, and QOL |
| **STUDY DESIGN** | mixed-methods cross-sectional online survey |
| **INTERVENTION** | NA |
| **INCLUSION CRITERIA** | Content analysis proposed by Bardin.(12) |
| **RECRUITMENT STRATEGY** | Participants were recruited through social media (health groups on Facebook and Instagram), university forums, and gym membership groups |
| **DATA COLLECTION METHOD** | Online survey with open ended questions |
| **THEORETICAL FRAMEWORK** | Data were analysed using a subtle realist lens which assumes existence of an objective truth however acknowledges that observation and inquiry is enmeshed within the perspective of, and contexts and concepts held by the researcher, but allows for meaningful interpretation of phenomena (Brooks et al., 2015; Hesse-Biber, 2016). A rich descriptive approach was adopted to determine important themes and insights on what is considered a poorly understood phenomenon (Kim et al., 2017). Inductive approach guided qualitative analysis of responses from the open-ended health communication question |
| **DATA ANALYSIS METHODOLOGY** | Mixed-methods - template analysis and logistic regressions |
| **FUNDING** | The authors received no financial support for the research, authorship, and/or publication of this article |
| **CONFLICT OF INTEREST** | The authors declared no potential conflicts of interest with respect to the research, authorship, and/or publication of this article |
| **ETHICS APPROVAL** | University Human Ethics Advisory Group (HEAG-H 10_2019) approved this study |
| **PRE-REGISTERED PROTOCOL IDENTIFICATION** | Not reported |

| **POPULATION CHARACTERISTICS** | **Katz et al. (2024)** |
| --- | --- |
| **SAMPLE SIZE** | 383 |
| **AGE** | 18–49 |
| **GENDER** | Women |
| **ETHNICITY** | Australian English 255 (66) Aboriginal/Torres Strait Islander 14 (4%) European 89 (23%) Other (Asian, African, Middle Eastern) 25 (7%) |
| **EMPLOYMENT** | Australian English 255 (66) Aboriginal/Torres Strait Islander 14 (4%) European 89 (23%) Other (Asian, African, Middle Eastern) 25 (7%) |
| **EDUCATION** | Year 12 or less 88 (23%) Vocational education (TAFE) 86 (22%) University degree (Bachelor/Master/PhD) 180 (47%) Other 29 (8%) |
| **RELATIONSHIP STATUS** | Single 145 (37%) Married/de Facto 217 (57%) Separated/Divorced 15 (4%) Other 6 (2%) |
| **SEXUALITY** | Not reported |
| **CHILDEREN** | Children: 1 or more n (%) 284 (74%) |
| **DIAGNOSES** | Endometriosis |
| **DIAGNOSIS METHOD** | Laparoscopy was the main form of diagnosis (85%) |
| **DURATION SINCE DIAGNOSIS** | Not reported |
| **DURATION OF ASSOCIATED SYMPTOMS** | Women reported on average, 15 years of period pain |
| **COMORBIDITIES** | Moderate to extreme fatigue on least fatigued day 111 (29%) Moderate to extreme fatigue on most fatigued day 340 (89%) Depression symptoms Mild – moderate 124 (32%) Severe – extremely severe 96 (25%) Anxiety symptoms Mild – moderate 107 (28%) Severe – extremely severe 121 (32%) Stress symptoms Mild – moderate 221 (58%) Severe – extremely severe 81 (21%) |

| **PUBLICATION SPECIFIC DATA** | **Katz (1995)** |
| --- | --- |
| **TITLE OF PAPER** | THE EXPERIENCE OF CHRONIC VULVAR PAIN: PSYCHOSOCIAL DIMENSIONS AND THE SENSE OF SELF |
| **COUNTRY/REGION OF ORIGIN** | USA |
| **STUDY AIM** | Explore the ways women make sense of their chronic vulvar pain using four psychological reference points (symptom perception, cognitive and emotional response, social situation, and identity issues) in a textual analytic exploration.  Explore how women with chronic vulvar pain make sense of their experience as related in their own terms |
| **STUDY DESIGN** | Qualitative |
| **INTERVENTION** | NA |
| **INCLUSION CRITERIA** | Had experienced ongoing or recurrent chronic vulvar pain symptoms for 1 year or more, over 18, no mental or psychological disability/illness |
| **RECRUITMENT STRATEGY** | Contacted providers (physicians, psychologists, nurses, and physical therapists) in California who treat chronic vulvar pain. Contacted director of Vulvar Pain Foundation who notified support group leaders in California |
| **DATA COLLECTION METHOD** | Narrative & semi structured interviews |
| **THEORETICAL FRAMEWORK** | Grounded in phenomenological, narrative, interactionist, and hermeneutic perspectives |
| **DATA ANALYSIS METHODOLOGY** | Analysis of the data following Hoshmand's (1989) description of grounded theory technique and hermeneutic interpretation, utilized constant comparative methods as propounded in the technique of grounded theory data analysis |
| **FUNDING** | Not reported |
| **CONFLICT OF INTEREST** | Not reported |
| **ETHICS APPROVAL** | The study was approved by the Research Ethics Committee of The Fielding Institute |
| **PRE-REGISTERED PROTOCOL IDENTIFICATION** | Not reported |

| **POPULATION CHARACTERISTICS** | **Katz (1995)** |
| --- | --- |
| **SAMPLE SIZE** | 16 |
| **AGE** | 20-65 |
| **GENDER** | Female |
| **ETHNICITY** | White 7 Caucasian 3 Mostly Irish 1 Hispanic 1 Black 1 European mix 1 White Anglo 1 Jewish 1" "relatively light skinned" |
| **EMPLOYMENT** | Not reported |
| **EDUCATION** | Master’s student 6 College student/2 years college/some college 3 High school diploma 1 L.Ac 1 J.D. (Juris Doctor) 1 Pharmacy degree 1 Bachelor of Arts 3 |
| **RELATIONSHIP STATUS** | Married 10 Committed relationship 2 Single 2 Committed lesbian relationship 1 Divorced 1 |
| **SEXUALITY** | Heterosexual 14 Lesbian 1 Bisexual 1 |
| **CHILDEREN** | 0: 14 1: 1 3: 1 |
| **DIAGNOSES** | Self-reported chronic vulvar pain (ongoing intractable symptoms of discomfort in the skin of the vulva and/or the vaginal vestibule, which may be diffuse or in specific loci, and either persistent or episodic, for at least 1 year of more) |
| **DIAGNOSIS METHOD** | Not reported |
| **DURATION SINCE DIAGNOSIS** | Not reported |
| **DURATION OF ASSOCIATED SYMPTOMS** | Avr. 5.4y, 1-14y |
| **COMORBIDITIES** | Not reported |

| **PUBLICATION SPECIFIC DATA** | **LePage & Selk (2016)** |
| --- | --- |
| **TITLE OF PAPER** | What Do Patients Want? A Needs Assessment of Vulvodynia Patients Attending a Vulvar Diseases Clinic |
| **COUNTRY/REGION OF ORIGIN** | Canada |
| **STUDY AIM** | The goal of this study was to identify unmet needs among localized provoked vulvodynia patients |
| **STUDY DESIGN** | Qualitative |
| **INTERVENTION** | NA |
| **INCLUSION CRITERIA** | Between 18 and 80 years old, have an established diagnosis of localized, provoked vulvodynia that had been confirmed by a physician in the VDC at WCH, and be capable of communicating in English |
| **RECRUITMENT STRATEGY** | A convenience method of sampling was used to recruit eligible participants from the Vulvar Diseases Clinic (VDC) at WCH |
| **DATA COLLECTION METHOD** | Semi-structured interviews using a pre-set interview guide of open-ended questions |
| **THEORETICAL FRAMEWORK** | Grounded theory |
| **DATA ANALYSIS METHODOLOGY** | Descriptive thematic analysis using the constant comparative method |
| **FUNDING** | None |
| **CONFLICT OF INTEREST** | The authors report no conflicts of interest |
| **ETHICS APPROVAL** | This study was approved by the Research Ethics Board at Women’s College Hospital (WCH) (2013-0080-E) |
| **PRE-REGISTERED PROTOCOL IDENTIFICATION** | Not reported |

| **POPULATION CHARACTERISTICS** | **LePage & Selk (2016)** |
| --- | --- |
| **SAMPLE SIZE** | 8 |
| **AGE** | 18-64y |
| **GENDER** | Female |
| **ETHNICITY** | Not reported |
| **EMPLOYMENT** | part time 12%, full time 38%, self-employed 25%, student 25%. Total annual household income: <25-150 k PA |
| **EDUCATION** | <=high school 12%, college 38%, university 25%, professional school 25% |
| **RELATIONSHIP STATUS** | single 38%, married/de facto 38%, divorced/separated 25% |
| **SEXUALITY** | Not reported |
| **CHILDEREN** | Not reported |
| **DIAGNOSES** | Localised, provoked vulvodynia |
| **DIAGNOSIS METHOD** | Confirmed by a physician in the VDC at WCH |
| **DURATION SINCE DIAGNOSIS** | Not reported |
| **DURATION OF ASSOCIATED SYMPTOMS** | Not reported |
| **COMORBIDITIES** | Not reported |

| **PUBLICATION SPECIFIC DATA** | **Lightbourne et al. (2023)** |
| --- | --- |
| **TITLE OF PAPER** | Living With Endometriosis: A Reflexive Thematic Analysis Examining Women’s Experiences with the Irish Healthcare Services |
| **COUNTRY/REGION OF ORIGIN** | Ireland |
| **STUDY AIM** | Aims to examine the experiences of 20 women with endometriosis in the Irish healthcare system in relation to diagnosis, treatment and support and to amplify their recommendations for service improvements |
| **STUDY DESIGN** | Qualitative, Thematic analysis of semi-structured interviews |
| **INTERVENTION** | NA |
| **INCLUSION CRITERIA** | Consists of three major stages: 1) pre-analysis; 2) material exploration; 3) treatment of results, inference and interpretation. Data analysis was carried out using systematic and objective procedures to describe what was obtained in the responses to the questionnaire (12) |
| **RECRUITMENT STRATEGY** | Purposive sampling, the Endometriosis Association of Ireland (EAI) aided study recruitment by emailing study information to their members |
| **DATA COLLECTION METHOD** | Online semi structured interviews |
| **THEORETICAL FRAMEWORK** | "Drawing on the work of the NCWI (2006, 2019), it takes a feminist and health equity approach with a primary focus on women’s lived experiences", constructionist perspective |
| **DATA ANALYSIS METHODOLOGY** | Braun & Clarke inductive reflexive thematic analysis |
| **FUNDING** | The author(s) received no financial support for the research, authorship, and/or publication of this article |
| **CONFLICT OF INTEREST** | The author(s) declared no potential conflicts of interest with respect to the research, authorship, and/or publication of this article |
| **ETHICS APPROVAL** | Institutional ethical approval was granted |
| **PRE-REGISTERED PROTOCOL IDENTIFICATION** | Not reported |

| **POPULATION CHARACTERISTICS** | **Lightbourne et al. (2023)** |
| --- | --- |
| **SAMPLE SIZE** | 20 |
| **AGE** | 18 - 50s |
| **GENDER** | Women |
| **ETHNICITY** | Not reported |
| **EMPLOYMENT** | Not reported |
| **EDUCATION** | Not reported |
| **RELATIONSHIP STATUS** | Not reported |
| **SEXUALITY** | Not reported |
| **CHILDEREN** | Not reported |
| **DIAGNOSES** | Endo |
| **DIAGNOSIS METHOD** | Laparoscopy |
| **DURATION SINCE DIAGNOSIS** | Time taken to establish a diagnosis ranged from 2 to 32 years, with an average of 6 to 7 years |
| **DURATION OF ASSOCIATED SYMPTOMS** | Not reported |
| **COMORBIDITIES** | Not reported |

| **PUBLICATION SPECIFIC DATA** | **Maena et al. (1999)** |
| --- | --- |
| **TITLE OF PAPER** | Psychosocial Correlates of Pain Attributions in Women with Dyspareunia |
| **COUNTRY/REGION OF ORIGIN** | Canada |
| **STUDY AIM** | Sought, in part, to investigate the causal attributions of women with dyspareunia and the relation of these attributions to the experience of pain, psychological adjustment, and relationship adjustment |
| **STUDY DESIGN** | Matched controlled clinical study |
| **INTERVENTION** | NA |
| **INCLUSION CRITERIA** | Inclusion criteria for study participation were 1) the experience of genital or pelvic pain with penile-vaginal intercourse and 2) fluency in English or French. Exclusion criteria were 1) chronic genital or pelvic pain at times other than during sexual intercourse, 2) pregnancy, and 3) dementia. |
| **RECRUITMENT STRATEGY** | Participants were recruited through the publication of two articles on the topic of dyspareunia, which appearedin two Montreal daily newspapers (one English and one French) |
| **DATA COLLECTION METHOD** | Qualitative data collected through psychosocial interviews. Additionally, self-report measures of pain, sexual attitudes, psychological distress, marital adjustment; and three gynaecological examinations were utilised |
| **THEORETICAL FRAMEWORK** | Causal attribution (Kleinman's method) |
| **DATA ANALYSIS METHODOLOGY** | Coding |
| **FUNDING** | Not reported |
| **CONFLICT OF INTEREST** | Not reported |
| **ETHICS APPROVAL** | Not reported |
| **PRE-REGISTERED PROTOCOL IDENTIFICATION** | Not reported |

| **POPULATION CHARACTERISTICS** | **Maena et al. (1999)** |
| --- | --- |
| **SAMPLE SIZE** | 100 |
| **AGE** | Mean age of 37.89 (standard deviation [SD]: 12.62). |
| **GENDER** | Female |
| **ETHNICITY** | Eighty were North American-born, 50 were primarily English speaking, 50 were primarily French speaking |
| **EMPLOYMENT** | Not reported |
| **EDUCATION** | The mean years of formal schooling was 14.77 (SD: 3.08), the equivalent of 1 year of undergraduate university. |
| **RELATIONSHIP STATUS** | Single with no regular sexual partner 15 Regular sexual partner 16 Cohabiting with partner 23 Married 46 |
| **SEXUALITY** | Not reported |
| **CHILDEREN** | The mean number of children was 0.68 (SD: 1.08) |
| **DIAGNOSES** | No pain related organic findings 25 Vulvar vestibulitis 49 Vaginal atrophy 11 Organic findings of different varieties that were neither atrophy nor vulvar vestibulitis 15 |
| **DIAGNOSIS METHOD** | Clinical gynaecological examination |
| **DURATION SINCE DIAGNOSIS** | Not reported |
| **DURATION OF ASSOCIATED SYMPTOMS** | Not reported |
| **COMORBIDITIES** | Not reported |

| **PUBLICATION SPECIFIC DATA** | **Myrtveit-Stensrud et al. (2023)** |
| --- | --- |
| **TITLE OF PAPER** | “It's all my fault”: a qualitative study of how heterosexual couples experience living with vulvodynia |
| **COUNTRY/REGION OF ORIGIN** | Norway |
| **STUDY AIM** | The aim of this study is to explore how heterosexual couples experience living with vulvodynia |
| **STUDY DESIGN** | Qualitative |
| **INTERVENTION** | NA |
| **INCLUSION CRITERIA** | Heterosexual couples in their 20s, where the woman had been diagnosed with vulvodynia, either localized, generalized, provoked,or unprovoked |
| **RECRUITMENT STRATEGY** | The women were recruited through a women's clinic at Oslo University Hospital, a physiotherapy clinic at Oslo Metropolitan University and from the patient organization for vulvodynia patients in Norway |
| **DATA COLLECTION METHOD** | Semi-structured interviews using an interview guide |
| **THEORETICAL FRAMEWORK** | Not Reported |
| **DATA ANALYSIS METHODOLOGY** | Thematic analysis |
| **FUNDING** | This article is part of a Ph.D. project funded by the Norwegian Women Public Health Association (project number 40017). |
| **CONFLICT OF INTEREST** | None |
| **ETHICS APPROVAL** | Approved by the Norwegian Centre for Research Data in Oslo, Norway (registration form 846535). Date of approval: May 3, 2021 |
| **PRE-REGISTERED PROTOCOL IDENTIFICATION** | Not reported |

| **POPULATION CHARACTERISTICS** | **Myrtveit-Stensrud et al. (2023)** |
| --- | --- |
| **SAMPLE SIZE** | 8 Women 7 Men |
| **AGE** | 19–31 years |
| **GENDER** | Women 8 Men 7 |
| **ETHNICITY** | Women: 8 Norwegian Men: 4 Norwegian, 3 other countries |
| **EMPLOYMENT** | One of the women was on sick leave due to another chronic pain condition. The remaining participants were either students or working full time. |
| **EDUCATION** | Not reported |
| **RELATIONSHIP STATUS** | The length of their relationships varied from 1 to 10 years. One couple was married |
| **SEXUALITY** | Heterosexual |
| **CHILDEREN** | None of the couples had children. |
| **DIAGNOSES** | Vulvodynia, either localized, generalized, provoked, or unprovoked |
| **DIAGNOSIS METHOD** | Diagnosed by gynaecologist |
| **DURATION SINCE DIAGNOSIS** | Not reported |
| **DURATION OF ASSOCIATED SYMPTOMS** | Not reported |
| **COMORBIDITIES** | Anxiety or depression |

| **PUBLICATION SPECIFIC DATA** | **Pithavadian (2021)** |
| --- | --- |
| **TITLE OF PAPER** | Understanding Women’s Help-Seeking Experiences for Vaginismus: Implications for Their Constructions of Sense of Self |
| **COUNTRY/REGION OF ORIGIN** | Australia |
| **STUDY AIM** | This project aims to understand Australian women’s help-seeking experiences for their vaginismus, and the impact of such help-seeking on their sense of self |
| **STUDY DESIGN** | A retrospective qualitative research design |
| **INTERVENTION** | NA |
| **INCLUSION CRITERIA** | 1) Received a medical diagnosis of vaginismus from a health professional; 2) Live in Australia; and 3) Be aged 18 years or above. |
| **RECRUITMENT STRATEGY** | In the hope of engaging participants from a diverse background, Service for the Treatment and Rehabilitation of Torture and Trauma Survivors (STARTTS), Community Migrant Resource Centre (CMRC), and Settlement Services International (SSI) were contacted. Please see Table 6.1 for a summary of the domains contacted for recruitment. Out of the 54 people, 23 heard about the project from Reddit subgroups, 11 from Facebook, six from an Endometriosis Support Group, three through word of mouth, three from Twitter, one from LinkedIn, and seven did not mention where they heard about the project |
| **DATA COLLECTION METHOD** | Semi structured interviews |
| **THEORETICAL FRAMEWORK** | Feminist theory |
| **DATA ANALYSIS METHODOLOGY** | Thematic analysis |
| **FUNDING** | NA |
| **CONFLICT OF INTEREST** | N |
| **ETHICS APPROVAL** | This project was approved by the Human Research Ethics Committee (HREC) at Western Sydney University (WSU) and given HREC Approval Number: H13618 on January 6th, 2020 |
| **PRE-REGISTERED PROTOCOL IDENTIFICATION** | N |

| **POPULATION CHARACTERISTICS** | **Pithavadian (2021)** |
| --- | --- |
| **SAMPLE SIZE** | 21 |
| **AGE** | 19-37, mean 27.6 |
| **GENDER** | 20 Female. One participant identified as non-binary gender, specifically agender |
| **ETHNICITY** | Five women identified from diverse non-white ethnic backgrounds. Sixteen participants identified as white, Caucasian, Anglo-Australian/Saxon, or European |
| **EMPLOYMENT** | Not reported |
| **EDUCATION** | Highschool-honours degree |
| **RELATIONSHIP STATUS** | Eight participants stated that they were single, and 13 women were in a relationship or married. |
| **SEXUALITY** | 17 women identified as heterosexual, two participants identified as bisexual, one participant stated that they were pansexual, and another participant identified as greysexual. This means that 19% of the sample did not identify as heterosexual (4/21 participants) |
| **CHILDEREN** | Not reported |
| **DIAGNOSES** | Vaginismus |
| **DIAGNOSIS METHOD** | Medical diagnosis from HCP |
| **DURATION SINCE DIAGNOSIS** | 0-14 years (mean 3.5y) |
| **DURATION OF ASSOCIATED SYMPTOMS** | Not reported |
| **COMORBIDITIES** | Twelve women stated that they also had pelvic pain co-morbidities. Nine of those women stated they had vulvodynia and/or endometriosis |

| **PUBLICATION SPECIFIC DATA** | **Savidge et al. (1998)** |
| --- | --- |
| **TITLE OF PAPER** | Women’s Perspectives on their Experiences of Chronic Pelvic Pain and Medical Care |
| **COUNTRY/REGION OF ORIGIN** | UK |
| **STUDY AIM** | To generate descriptive data regarding women’s involvement with professionals, and their perceptions of the pain, and to gain insight into factors perceived as helpful or unhelpful with regard to care |
| **STUDY DESIGN** | Qualitative |
| **INTERVENTION** | NA |
| **INCLUSION CRITERIA** | 1. The participant was aged 18 or over 2. She had experienced CPP for at least 6 months prior to the laparoscopy 3. The laparoscopy identified no recognizable organic pathology considered sufficient to explain the pain 4. The laparoscopy was performed 12 to 18 months previously. |
| **RECRUITMENT STRATEGY** | Consultation of operating lists for diagnostic laparoscopy over an 8-month period at two teaching hospitals in Sheffield |
| **DATA COLLECTION METHOD** | Semi structured interviews |
| **THEORETICAL FRAMEWORK** | Not reported |
| **DATA ANALYSIS METHODOLOGY** | Qualitative data analysis is described by Dey (1993) |
| **FUNDING** | Not reported |
| **CONFLICT OF INTEREST** | Not reported |
| **ETHICS APPROVAL** | Not reported |
| **PRE-REGISTERED PROTOCOL IDENTIFICATION** | Not reported |

| **POPULATION CHARACTERISTICS** | **Savidge et al. (1998)** |
| --- | --- |
| **SAMPLE SIZE** | 21 |
| **AGE** | Mean age of the participants was 34.2 years (SD 5 10.08, range 5 21–61) |
| **GENDER** | Female |
| **ETHNICITY** | All described themselves as White/European |
| **EMPLOYMENT** | 15 Employed  3 Housewives 2 Unemployed 1 Retired |
| **EDUCATION** | Not reported |
| **RELATIONSHIP STATUS** | 13 Married or cohabiting 8 Single |
| **SEXUALITY** | Not reported |
| **CHILDEREN** | 16 had children |
| **DIAGNOSES** | Chronic pelvic pain |
| **DIAGNOSIS METHOD** | Diagnosis of exclusion (negative laparoscopy) |
| **DURATION SINCE DIAGNOSIS** | Not reported (had the laparoscopy 12-18 months ago) |
| **DURATION OF ASSOCIATED SYMPTOMS** | The mean number of years for which women had experienced CPP was 6.5 (SD 5 5.57), with a range of 1 to 19 years |
| **COMORBIDITIES** | Not reported |

| **PUBLICATION SPECIFIC DATA** | **Shallcross et al. (2019)** |
| --- | --- |
| **TITLE OF PAPER** | Women’s Experiences of Vulvodynia: An Interpretative Phenomenological Analysis of the Journey Toward Diagnosis |
| **COUNTRY/REGION OF ORIGIN** | UK |
| **STUDY AIM** | The aim of the current study was to explore the experiences of women with vulvodynia on their journey toward diagnosis, by using semi-structured interviews and an interpretative phenomenological analysis (IPA) methodology |
| **STUDY DESIGN** | Qualitative |
| **INTERVENTION** | NA |
| **INCLUSION CRITERIA** | Women with medical diagnosis of vulvodynia (diagnosed by a qualified medical practitioner), over 18 years of able, and able to speak and understand sufficient English to take part in the interview. Diagnosis could not be less than 6 months ago and no more than 7 years ago |
| **RECRUITMENT STRATEGY** | Women living in the North-West of England, who were also supporters of the VpS were invited to take part in the study via VPS website advertisements and email lists |
| **DATA COLLECTION METHOD** | Semi structured interviews |
| **THEORETICAL FRAMEWORK** | Phenomenology |
| **DATA ANALYSIS METHODOLOGY** | Interpretative phenomenological analysis (IPA) |
| **FUNDING** | This study was funded as part of the lead author’s Clinical Psychology Training at the University of Liverpool |
| **CONFLICT OF INTEREST** | The authors declare that they have no conflict of interest |
| **ETHICS APPROVAL** | Ethical approval was obtained from a University Research Ethics Committee |
| **PRE-REGISTERED PROTOCOL IDENTIFICATION** | Not reported |

| **POPULATION CHARACTERISTICS** | **Shallcross et al. (2019)** |
| --- | --- |
| **SAMPLE SIZE** | 8 |
| **AGE** | Between 23 and 70 years |
| **GENDER** | Women 8 Men 7 |
| **ETHNICITY** | White, British women |
| **EMPLOYMENT** | Not reported |
| **EDUCATION** | Not reported |
| **RELATIONSHIP STATUS** | Single 2 Boyfriend 1 Complicated 1 Relationship 1 Married 3 |
| **SEXUALITY** | Heterosexual |
| **CHILDEREN** | Not reported |
| **DIAGNOSES** | Vulvodynia |
| **DIAGNOSIS METHOD** | Not specified - by a qualified medical practitioner |
| **DURATION SINCE DIAGNOSIS** | <7 years |
| **DURATION OF ASSOCIATED SYMPTOMS** | Not reported |
| **COMORBIDITIES** | Spastic paraplegia 1 Lichen Sclerosis 1 Vulval eczema 1 Urethral syndrome 1 Fissures 1 Varicose vein 1 Pudendal neuropathy 1 MRI revealed some scar tissue 1 |

| **PUBLICATION SPECIFIC DATA** | **Twiss (2018)** |
| --- | --- |
| **TITLE OF PAPER** | NARRATIVES OF YOUNG WOMEN LIVING WITH CHRONIC PELVIC PAIN |
| **COUNTRY/REGION OF ORIGIN** | USA |
| **STUDY AIM** | Examine the lived experiences of three women living with chronic pelvic pain including treatment experiences as well as the impact of these experiences on the psychological wellbeing of patients through a sociocultural lens |
| **STUDY DESIGN** | Narrative inquiry |
| **INTERVENTION** | NA |
| **INCLUSION CRITERIA** | Aged 18-30 years of age and had either a) experienced and sought treatment for chronic pelvic pain related to endometriosis prior to the date of the interview or b) received a diagnosis of endometriosis during adolescence or your adulthood |
| **RECRUITMENT STRATEGY** | Posting a flyer on social media websites requesting participation in the study, purposeful sampling. |
| **DATA COLLECTION METHOD** | Semi-structured interviews |
| **THEORETICAL FRAMEWORK** | Feminist, post modern |
| **DATA ANALYSIS METHODOLOGY** | Riessman 1993 - chronological narratives, Gilligan’s listening guide method (2015) |
| **FUNDING** | Not reported |
| **CONFLICT OF INTEREST** | Not reported |
| **ETHICS APPROVAL** | Not reported |
| **PRE-REGISTERED PROTOCOL IDENTIFICATION** | Not reported |

| **POPULATION CHARACTERISTICS** | **Twiss (2018)** |
| --- | --- |
| **SAMPLE SIZE** | 3 |
| **AGE** | 25, 29, 30 years |
| **GENDER** | Female |
| **ETHNICITY** | Latina 1 White 2 |
| **EMPLOYMENT** | Not reported |
| **EDUCATION** | Not reported |
| **RELATIONSHIP STATUS** | Married 2 Committed relationship 1 |
| **SEXUALITY** | Heterosexual |
| **CHILDEREN** | 2-year-old daughter: 1  No children: 2 |
| **DIAGNOSES** | chronic pelvic pain, endometriosis, ovarian cysts, |
| **DIAGNOSIS METHOD** | Not reported |
| **DURATION SINCE DIAGNOSIS** | Not reported |
| **DURATION OF ASSOCIATED SYMPTOMS** | 9 years  2 years  13 years   av. 9 |
| **COMORBIDITIES** | Not reported |

| **PUBLICATION SPECIFIC DATA** | **Ward & Ogden (1994)** |
| --- | --- |
| **TITLE OF PAPER** | Experiencing vaginismus— Sufferers' beliefs about causes and effects |
| **COUNTRY/REGION OF ORIGIN** | UK |
| **STUDY AIM** | The aim of the present study was to examine the feelings, experiences and cognitions of vaginismus sufferers and to examine ideas of causality and consequences of vaginismus from the sufferer’s point of view using both quantitative and qualitative methodologies |
| **STUDY DESIGN** | Mixed methods |
| **INTERVENTION** | NA |
| **INCLUSION CRITERIA** | Sufferers and ex-sufferers of vaginismus |
| **RECRUITMENT STRATEGY** | Questionnaires sent out through advertisement in a newspaper, Resolve support group, Relate, Institute of Psychosexual Medicine, and psychosexual therapists in Manchester, the USA, & Australia |
| **DATA COLLECTION METHOD** | Free text in questionnaires |
| **THEORETICAL FRAMEWORK** | Not reported |
| **DATA ANALYSIS METHODOLOGY** | Discourse analysis |
| **FUNDING** | Not reported |
| **CONFLICT OF INTEREST** | Not reported |
| **ETHICS APPROVAL** | Not reported |
| **PRE-REGISTERED PROTOCOL IDENTIFICATION** | Not reported |

| **POPULATION CHARACTERISTICS** | **Ward & Ogden (1994)** |
| --- | --- |
| **SAMPLE SIZE** | 89 |
| **AGE** | 21 to 71 with a mean age of 33.6 2 9.74 |
| **GENDER** | Not reported, language suggests all female |
| **ETHNICITY** | Not reported |
| **EMPLOYMENT** | Came from a wide variety of social classes, and with their occupations including administrators, civil servants, hairdressers, teachers, nurses, secretaries, lecturers, clerks, housewives and librarians |
| **EDUCATION** | Not reported |
| **RELATIONSHIP STATUS** | 71.9% married/de facto, 14.6% single, 4.5% separated/divorced, 7.9% in a relationship |
| **SEXUALITY** | 95.5% heterosexual, 2.2% bisexual, 1.1% "Claimed to have no sexual feelings or orientation", 1.1% did not complete this section |
| **CHILDEREN** | 48.30% |
| **DIAGNOSES** | Vaginismus |
| **DIAGNOSIS METHOD** | Not reported |
| **DURATION SINCE DIAGNOSIS** | Not reported |
| **DURATION OF ASSOCIATED SYMPTOMS** | Ranged from one year to a whole lifetime |
| **COMORBIDITIES** | Not reported |

| **PUBLICATION SPECIFIC DATA** | **Young & Miller (2019)** |
| --- | --- |
| **TITLE OF PAPER** | "This girl is on fire". Sensemaking in an online health community for vulvodynia |
| **COUNTRY/REGION OF ORIGIN** | USA |
| **STUDY AIM** | Examines the ways that women with vulvodynia co-construct an understanding for their condition, build personalised management plans for their individual symptoms, and devise ways to be taken seriously by medical professionals. It examines how the uncertainty of the condition impacts participants' emotional and interpersonal wellbeing |
| **STUDY DESIGN** | Qualitative |
| **INTERVENTION** | NA |
| **INCLUSION CRITERIA** | Members of a private vulvodynia Facebook group |
| **RECRUITMENT STRATEGY** | Facebook vulvodynia support group - 13000 members at time of observation |
| **DATA COLLECTION METHOD** | Participant observations of one vulvodynia Facebook group and open-ended interviews with group members, 11 open ended interviews |
| **THEORETICAL FRAMEWORK** | Not reported |
| **DATA ANALYSIS METHODOLOGY** | Thematic analysis |
| **FUNDING** | Not reported |
| **CONFLICT OF INTEREST** | Not reported |
| **ETHICS APPROVAL** | Ethics approval was obtained from Indiana University’s Human Research & Institutional Review Board prior to con-ducting the research |
| **PRE-REGISTERED PROTOCOL IDENTIFICATION** | Not reported |

| **POPULATION CHARACTERISTICS** | **Young & Miller (2019)** |
| --- | --- |
| **SAMPLE SIZE** | 11 |
| **AGE** | 23 to 65 years old, with a median age of 36.4 |
| **GENDER** | Female |
| **ETHNICITY** | Lived in:  United States 8 Australia 1 Canada 1 Columbia 1 |
| **EMPLOYMENT** | Not reported |
| **EDUCATION** | Not reported |
| **RELATIONSHIP STATUS** | Boyfriend 3 Married 6 Single 2 |
| **SEXUALITY** | Heterosexual |
| **CHILDEREN** | Not reported |
| **DIAGNOSES** | Vulvodynia |
| **DIAGNOSIS METHOD** | Not reported |
| **DURATION SINCE DIAGNOSIS** | Not reported |
| **DURATION OF ASSOCIATED SYMPTOMS** | Not reported |
| **COMORBIDITIES** | Not reported |

Ballard, K., Lowton, K., & Wright, J. (2006). What’s the delay? A qualitative study of women’s experiences of reaching a diagnosis of endometriosis. *Fertility and Sterility*, *86*(5), 1296-1301. https://doi.org/10.1016/j.fertnstert.2006.04.054

Boge-Olsnes, C. M., Risor, M. B., & Oberg, G. K. (2023). How life events are perceived to link to bodily distress: A qualitative study of women with chronic pelvic pain. *Health Care for Women International*, *44*(9), 1218-1238. https://doi.org/https://dx.doi.org/10.1080/07399332.2022.2087076

Boge-Olsnes, C., Bech Risør, M., & Øberg, G. K. (2022). Chronic pelvic pain sufferers' experiences of Norwegian psychomotor physiotherapy: a qualitative study on an embodied approach to pain. *European Journal of Physiotherapy*, *26*(1), 2-11. https://doi.org/10.1080/21679169.2022.2136754

Burbeck, R., & Willig, C. (2014). The personal experience of dysmenorrhoea: An interpretative phenomenological analysis. *Journal of Health Psychology*, *19*(10), 1334-1344. https://doi.org/10.1177/1359105313490313

Chen, C. X., Draucker, C. B., & Carpenter, J. S. (2018). What women say about their dysmenorrhea: a qualitative thematic analysis. *BMC Women's Health*, *18*(1), 47. https://doi.org/10.1186/s12905-018-0538-8

Crouch, S. M., Baranoff, J. A., Bunzli, S., Mardon, A. K., & Chalmers, K. J. (2024). I am in Charge, But Not Always in Control. *Clinical Journal of Pain*, *40*(11), 673-683. https://doi.org/10.1097/AJP.0000000000001242

Danielsen, K. G., Dahl-Michelsen, T., Håkonsen, E., & Haugstad, G. K. (2019). Recovering from provoked vestibulodynia: Experiences from encounters with somatocognitive therapy. *Physiotherapy Theory and Practice*, *35*(3), 1-228. https://doi.org/10.1080/09593985.2018.1442540

de Jesus, L. M., de Sousa, M. B. D., de Oliveira, N. P., Santos, L. F., Conde, D. M., Pacheco, L. R., & de Deus, J. M. (2024). Chronic pelvic pain in women: the path to outpatient discharge. *Africa Journal of Nursing and Midwifery*, *37*, 1-9. https://doi.org/https://dx.doi.org/10.37689/acta-ape/2024AO00028822

Denny, E. (2009). “I Never Know From One Day to Another How I Will Feel”: Pain and Uncertainty in Women With Endometriosis. *Qualitative Health Research*, *19*(7), 985-995. https://doi.org/10.1177/1049732309338725

Dogan, J. N., Thorpe, S. Y., Malone, N., Jester, J., Stevens-Watkins, D., & Hargons, C. (2023). 'My partner will think I'm weak or overthinking my pain': how being superwoman inhibits Black women's sexual pain disclosure to their partners. *Culture, Health & Sexuality*, *25*(5), 567-581. https://doi.org/10.1080/13691058.2022.2072956

Donaldson, R. L., & Meana, M. (2011). Early Dyspareunia Experience in Young Women: Confusion, Consequences, and Help‐seeking Barriers. *Journal of Sexual Medicine*, *8*(3), 814-823. https://doi.org/10.1111/j.1743-6109.2010.02150.x

Girard, E., Mazloum, A., Navarria-Forney, I., Pluchino, N., Streuli, I., & Cedraschi, C. (2023). Women's lived experience of endometriosis-related fertility issues. *PLoS ONE*, *18*(11), e0293531.

Glazer, E. (2012). *Embodiment, pain, and circumcision in Somali-Canadian women* [Masters thesis, University of Toronto]. ProQuest Dissertations and Theses Global.

Grace, V. M., & MacBride-Stewart, S. (2007). 'Women get this': gendered meanings of chronic pelvic pain. *Health*, *11*(1), 47-67. https://doi.org/10.1177/1363459307070803

Groven, K. S., Råheim, M., Håkonsen, E., & Haugstad, G. K. (2016). "Will I ever be a true woman?" An exploration of the experiences of women with vestibulodynia. *Health Care for Women International*, *37*(8), 818-835. https://doi.org/10.1080/07399332.2015.1103739

Hamilton, E. S. (2010). *A grounded theory of coping among women with chronic pelvic pain* [Doctoral thesis, Saybrook University]. ProQuest Dissertations and Theses Global.

Helosvuori, E., & Oikkonen, V. (2024). Sensing pain: Embodied knowledge in endometriosis. *Health, 28*(6), 937-952. https://doi.org/https://dx.doi.org/10.1177/13634593231214938

Huntington, A., & Gilmour, J. A. (2005). A life shaped by pain: women and endometriosis. *Journal of Clinical Nursing*, *14*(9), 1124-1132. https://doi.org/10.1111/j.1365-2702.2005.01231.x

Katz, C., Evans, S., & Mikocka-Walus, A. (2025). 'Listen to women as if they were your most cherished person': Australian women's perspectives on living with the pain of endometriosis: A mixed-methods study. *Journal of health psychology*, *30*(8), 1899-1914. https://doi.org/10.1177/13591053241250101

Katz, S. R. (1995). *The experience of chronic vulvar pain: Psychosocial dimensions and the sense of self* [Doctoral thesis, University of Lethbridge]. ProQuest Dissertations and Theses Global.

LePage, K., & Selk, A. (2016). What Do Patients Want? A Needs Assessment of Vulvodynia Patients Attending a Vulvar Diseases Clinic. *Journal of Sexual Medicine*, *4*(4), e242-e248. https://doi.org/10.1016/j.esxm.2016.06.003

Lightbourne, A., Foley, S., Dempsey, M., & Cronin, M. (2023). Living With Endometriosis: A Reflexive Thematic Analysis Examining Women's Experiences With the Irish Healthcare Services. *Qualitative Health Research*. https://doi.org/https://dx.doi.org/10.1177/10497323231214114

Meana, M., Binik, Y. M., Khalife, S., & Cohen, D. (1999). Psychosocial Correlates of Pain Attributions in Women With Dyspareunia. *Psychosomatics*, *40*(6), 497-502. https://doi.org/10.1016/S0033-3182(99)71188-6

Myrtveit-Stensrud, L., Haugstad, G. K., Rème, S. E., Schaller, S. L., & Groven, K. S. (2023). "It's all my fault": a qualitative study of how heterosexual couples experience living with vulvodynia. *Acta Obstetricia et Gynecologica Scandinavica*, *102*(10), 1378-1389. https://doi.org/10.1111/aogs.14537

Pithavadian, R. (2021). *Understanding Women’s Help-Seeking Experiences for Vaginismus: Implications for Their Constructions of Sense of Self* [Masters thesis, Western Sydney University]. Sydney, Australia.

Savidge, C. J., Slade, P., Stewart, P., & Li, T. C. (1998). Women's Perspectives on their Experiences of Chronic Pelvic Pain and Medical Care. *Journal of Health Psychology*, *3*(1), 103-116. https://doi.org/10.1177/135910539800300108

Shallcross, R., Dickson, J. M., Nunns, D., Taylor, K., & Kiemle, G. (2019). Women’s Experiences of Vulvodynia: An Interpretative Phenomenological Analysis of the Journey Toward Diagnosis. *Archives of Sexual Behavior*, *48*(3), 961-974. https://doi.org/10.1007/s10508-018-1246-z

Twiss, C. E. (2018). *Narratives of Young Women Living with Chronic Pelvic Pain* [Doctoral thesis, New Mexico State University]. ProQuest Dissertations and Theses Global.

Ward, E., & Ogden, J. (2010). Experiencing vaginismus-sufferers' beliefs about causes and effects. *Sexual and Relationship Therapy*, *25*(4), 434-446. https://doi.org/10.1080/14681994.2010.518724

Young, A. L., & Miller, A. D. (2019). "This Girl is on Fire" Sensemaking in an Online Health Community for Vulvodynia. Proceedings of the 2019 CHI Conference on Human Factors in Computing Systems, 1-13. https://doi.org/10.1145/3290605.3300359

# Table S3. Appraisal of reporting using the COREQ checklist (Tong et al., 2007)

|  | Ballard et al. (2006) | Boge-Olsenes et al. (2023) | Boge-Olsnes et al. (2022) | Burbeck & Willig (2014) | Chen et al. (2018) | Crouch et al. (2024) | Danielsen et al. (2019) | de Jesus et al. (2024) | Denny (2009) | Dogan et al. (2022) | Donaldson & Meana (2011) | Girard et al. (2023) | Glazer (2010) | Grace & MacBride-Stewart (2007) | Groven et al. (2016) | Hamilton (2011) | Helosvuori & Oikkonen (2024) | Huntington & Gilmour (2005) | Katz et al. (2024) | Katz (1995) | LePage & Selk (2016) | Lightbourne et al. (2023) | Maena et al. (1999) | Myrtveit-Stensrud et al. (2023) | Pithavadian (2021) | Savidge et al. (1998) | Shallcross et al. (2019) | Twiss (2018) | Ward & Ogden (1994) | Young & Miller (2019) |
| --- | --- | --- | --- | --- | --- | --- | --- | --- | --- | --- | --- | --- | --- | --- | --- | --- | --- | --- | --- | --- | --- | --- | --- | --- | --- | --- | --- | --- | --- | --- |
| 1. Reported which author/s conducted the interview or focus group. | Y | Y | Y | Y | NA | Y | Y | N | Y | NA | N | Y | Y | N | Y | Y | Y | N | NA | Y | Y | N | N | Y | Y | Y | Y | Y | NA | N |
| 2. Reported the researcher’s credentials. | Y | Y | Y | N | Y | Y | N | N | Y | N | Y | N | Y | N | N | Y | N | Y | Y | Y | Y | N | Y | N | Y | Y | N | Y | Y | N |
| 3. Reported their occupation at the time of the study. | Y | Y | Y | N | Y | Y | N | N | Y | N | N | N | Y | Y | N | N | Y | Y | Y | N | Y | N | N | Y | Y | Y | Y | Y | Y | N |
| 4. Reported whether the researcher was male or female. | N | N | N | N | N | Y | N | N | N | Y | N | Y | N | N | Y | N | Y | N | N | Y | Y | N | N | N | N | Y | Y | N | N | Y |
| 5. Reported what experience or training the researcher had. | N | Y | Y | N | Y | Y | N | N | Y | N | N | Y | N | Y | N | N | N | N | Y | N | Y | Y | Y | Y | N | Y | Y | Y | N | N |
| 6. Reported whether relationships were established prior to study commencement. | N | N | Y | N | N | Y | N | Y | Y | Y | N | Y | Y | N | Y | N | N | N | NA | Y | Y | N | N | Y | N | N | Y | Y | N | Y |
| 7. Reported what the participants knew about the researcher. | N | N | N | N | N | Y | N | Y | Y | N | N | N | N | N | N | N | N | N | N | N | N | N | N | N | N | N | N | Y | N | Y |
| 8. Reported characteristics about the interviewer/facilitator. | N | Y | Y | Y | N | Y | N | N | Y | Y | N | N | Y | Y | N | Y | N | N | Y | Y | N | Y | N | N | N | Y | Y | Y | N | Y |
| 9. Reported what methodological orientation was stated to underpin the study. | N | Y | Y | Y | N | Y | N | Y | Y | Y | Y | Y | Y | Y | Y | Y | Y | Y | Y | Y | Y | Y | Y | N | Y | N | Y | Y | N | N |
| 10. Reported how were participants selected. | Y | Y | Y | Y | Y | Y | Y | Y | Y | Y | Y | Y | Y | Y | Y | Y | Y | Y | Y | Y | Y | Y | Y | Y | Y | Y | Y | Y | Y | Y |
| 11. Reported how participants were approached. | N | Y | Y | N | N | Y | Y | Y | N | Y | Y | N | N | N | N | Y | Y | Y | Y | N | N | Y | Y | N | Y | N | Y | Y | Y | Y |
| 12. Reported how many participants were in the study. | Y | Y | Y | Y | Y | Y | Y | Y | Y | Y | Y | Y | Y | Y | Y | Y | Y | Y | Y | Y | Y | Y | Y | Y | Y | Y | Y | Y | Y | Y |
| 13. Reported how many people refused to participate or dropped out. Reported reasons. | N | N | N | Y | Y | Y | N | N | N | Y | Y | N | N | N | N | Y | N | N | Y | Y | Y | N | Y | N | Y | Y | Y | N | Y | Y |
| 14. Reported where the data was collected. | Y | Y | Y | Y | NA | Y | Y | Y | Y | NA | Y | Y | Y | N | N | N | N | N | Y | N | Y | Y | Y | Y | Y | Y | N | N | NA | Y |
| 15. Reported whether anyone else was present besides the participants and researchers. | N | N | Y | Y | NA | Y | Y | N | N | NA | N | N | N | N | Y | N | N | N | NA | N | Y | N | N | Y | N | N | N | N | NA | N |
| 16. Reported the important characteristics of the sample. | Y | Y | Y | Y | Y | Y | Y | Y | Y | Y | Y | N | Y | Y | Y | Y | N | Y | Y | Y | Y | N | Y | Y | Y | Y | Y | Y | Y | Y |
| 17. Reported whether questions, prompts, guides, were provided by the authors. Reported whether it was pilot tested. | N | N | N | Y | N | Y | N | N | N | N | N | Y | N | N | N | N | N | N | N | Y | N | N | N | N | N | Y | N | N | Y | N |
| 18. Reported whether repeat interviews were carried out and if yes, how many. | Y | N | Y | N | NA | N | N | N | Y | NA | N | N | N | N | N | Y | N | N | NA | Y | Y | N | N | N | N | N | N | N | NA | N |
| 19. Reported whether the research used audio or visual recording to collect the data. | Y | N | Y | Y | NA | Y | Y | N | Y | Y | Y | N | Y | Y | Y | Y | N | Y | NA | Y | Y | N | Y | N | Y | Y | N | Y | Y | Y |
| 20. Reported whether field notes were made during and/or after the interview or focus group. | N | Y | Y | N | NA | N | Y | N | N | Y | N | N | N | N | N | Y | N | N | Y | Y | Y | Y | N | N | N | Y | N | Y | NA | Y |
| 21.Reported the duration of the interviews or focus group. | Y | Y | Y | N | NA | Y | Y | N | N | Y | N | Y | N | N | N | Y | N | N | Y | Y | Y | Y | Y | Y | Y | N | Y | Y | N | Y |
| 22. Was data saturation discussed? | N | N | N | Y | N | Y | N | Y | Y | N | N | Y | Y | N | N | N | N | N | NA | Y | Y | NA | N | Y | Y | N | N | N | N | N |
| 23. Reported if transcripts were returned to participants for comment and/or correction. | N | N | N | N | N | Y | N | N | Y | N | N | Y | N | N | N | N | N | N | NA | Y | Y | N | N | N | Y | N | N | Y | N | N |
| 24. Reported how many data coders coded the data. | Y | Y | Y | N | Y | Y | Y | N | N | Y | Y | Y | Y | N | N | Y | Y | N | Y | Y | Y | Y | Y | Y | N | Y | Y | Y | N | N |
| 25. Did authors provide a description of the coding tree? | Y | Y | Y | N | Y | Y | Y | Y | N | N | Y | N | Y | Y | N | N | N | N | N | Y | Y | N | N | Y | Y | Y | Y | N | N | N |
| 26. Reported whether themes were identified in advance or derived from the data. | Y | Y | Y | Y | Y | Y | Y | Y | Y | Y | Y | Y | Y | Y | Y | Y | Y | Y | Y | Y | Y | Y | Y | Y | Y | Y | Y | Y | N | Y |
| 27. Reported what software, if applicable, was used to manage the data. | N | Y | Y | N | N | Y | N | N | N | N | N | N | Y | Y | N | Y | N | N | Y | Y | N | N | N | N | Y | N | Y | N | N | N |
| 28. Reported whether participants provided feedback on the findings. | N | N | N | N | N | Y | N | N | Y | N | N | N | N | N | N | N | N | Y | N | Y | Y | N | N | N | N | N | N | Y | N | N |
| 29. Were participant quotations presented to illustrate the themes/findings? Was each quotation identified? | Y | Y | Y | Y | N | Y | Y | Y | N | N | Y | Y | Y | Y | Y | Y | Y | Y | Y | Y | Y | Y | N | Y | Y | Y | Y | Y | Y | Y |
| 30. Was there consistency between the data presented and the findings? | Y | Y | Y | Y | Y | Y | Y | Y | Y | Y | Y | Y | Y | Y | Y | Y | Y | Y | Y | Y | Y | Y | Y | Y | Y | Y | Y | Y | Y | Y |
| 31. Were major themes clearly presented in the findings? | Y | Y | Y | Y | Y | Y | Y | Y | Y | Y | Y | Y | Y | Y | Y | Y | Y | Y | Y | Y | Y | Y | Y | Y | Y | Y | Y | Y | N | Y |
| 32. Is there a description of diverse cases or discussion of minor themes? | Y | N | Y | Y | Y | Y | N | N | Y | Y | Y | Y | Y | Y | Y | Y | N | Y | Y | Y | Y | N | Y | Y | Y | Y | Y | Y | N | Y |

Ballard, K., Lowton, K., & Wright, J. (2006). What’s the delay? A qualitative study of women’s experiences of reaching a diagnosis of endometriosis. *Fertility and Sterility*, *86*(5), 1296-1301. https://doi.org/10.1016/j.fertnstert.2006.04.054

Boge-Olsnes, C. M., Risor, M. B., & Oberg, G. K. (2023). How life events are perceived to link to bodily distress: A qualitative study of women with chronic pelvic pain. *Health Care for Women International*, *44*(9), 1218-1238. https://doi.org/https://dx.doi.org/10.1080/07399332.2022.2087076

Boge-Olsnes, C., Bech Risør, M., & Øberg, G. K. (2022). Chronic pelvic pain sufferers' experiences of Norwegian psychomotor physiotherapy: a qualitative study on an embodied approach to pain. *European Journal of Physiotherapy*, *26*(1), 2-11. https://doi.org/10.1080/21679169.2022.2136754

Burbeck, R., & Willig, C. (2014). The personal experience of dysmenorrhoea: An interpretative phenomenological analysis. *Journal of Health Psychology*, *19*(10), 1334-1344. https://doi.org/10.1177/1359105313490313

Chen, C. X., Draucker, C. B., & Carpenter, J. S. (2018). What women say about their dysmenorrhea: a qualitative thematic analysis. *BMC Women's Health*, *18*(1), 47. https://doi.org/10.1186/s12905-018-0538-8

Crouch, S. M., Baranoff, J. A., Bunzli, S., Mardon, A. K., & Chalmers, K. J. (2024). I am in Charge, But Not Always in Control. *Clinical Journal of Pain*, *40*(11), 673-683. https://doi.org/10.1097/AJP.0000000000001242

Danielsen, K. G., Dahl-Michelsen, T., Håkonsen, E., & Haugstad, G. K. (2019). Recovering from provoked vestibulodynia: Experiences from encounters with somatocognitive therapy. *Physiotherapy Theory and Practice*, *35*(3), 1-228. https://doi.org/10.1080/09593985.2018.1442540

de Jesus, L. M., de Sousa, M. B. D., de Oliveira, N. P., Santos, L. F., Conde, D. M., Pacheco, L. R., & de Deus, J. M. (2024). Chronic pelvic pain in women: the path to outpatient discharge. *Africa Journal of Nursing and Midwifery*, *37*, 1-9. https://doi.org/https://dx.doi.org/10.37689/acta-ape/2024AO00028822

Denny, E. (2009). “I Never Know From One Day to Another How I Will Feel”: Pain and Uncertainty in Women With Endometriosis. *Qualitative Health Research*, *19*(7), 985-995. https://doi.org/10.1177/1049732309338725

Dogan, J. N., Thorpe, S. Y., Malone, N., Jester, J., Stevens-Watkins, D., & Hargons, C. (2023). 'My partner will think I'm weak or overthinking my pain': how being superwoman inhibits Black women's sexual pain disclosure to their partners. *Culture, Health & Sexuality*, *25*(5), 567-581. https://doi.org/10.1080/13691058.2022.2072956

Donaldson, R. L., & Meana, M. (2011). Early Dyspareunia Experience in Young Women: Confusion, Consequences, and Help‐seeking Barriers. *Journal of Sexual Medicine*, *8*(3), 814-823. https://doi.org/10.1111/j.1743-6109.2010.02150.x

Girard, E., Mazloum, A., Navarria-Forney, I., Pluchino, N., Streuli, I., & Cedraschi, C. (2023). Women's lived experience of endometriosis-related fertility issues. *PLoS ONE*, *18*(11), e0293531.

Glazer, E. (2012). *Embodiment, pain, and circumcision in Somali-Canadian women* [Masters thesis, University of Toronto]. ProQuest Dissertations and Theses Global.

Grace, V. M., & MacBride-Stewart, S. (2007). 'Women get this': gendered meanings of chronic pelvic pain. *Health*, *11*(1), 47-67. https://doi.org/10.1177/1363459307070803

Groven, K. S., Råheim, M., Håkonsen, E., & Haugstad, G. K. (2016). "Will I ever be a true woman?" An exploration of the experiences of women with vestibulodynia. *Health Care for Women International*, *37*(8), 818-835. https://doi.org/10.1080/07399332.2015.1103739

Hamilton, E. S. (2010). *A grounded theory of coping among women with chronic pelvic pain* [Doctoral thesis, Saybrook University]. ProQuest Dissertations and Theses Global.

Helosvuori, E., & Oikkonen, V. (2024). Sensing pain: Embodied knowledge in endometriosis. *Health, 28*(6), 937-952. https://doi.org/https://dx.doi.org/10.1177/13634593231214938

Huntington, A., & Gilmour, J. A. (2005). A life shaped by pain: women and endometriosis. *Journal of Clinical Nursing*, *14*(9), 1124-1132. https://doi.org/10.1111/j.1365-2702.2005.01231.x

Katz, C., Evans, S., & Mikocka-Walus, A. (2025). 'Listen to women as if they were your most cherished person': Australian women's perspectives on living with the pain of endometriosis: A mixed-methods study. *Journal of health psychology*, *30*(8), 1899-1914. https://doi.org/10.1177/13591053241250101

Katz, S. R. (1995). *The experience of chronic vulvar pain: Psychosocial dimensions and the sense of self* [Doctoral thesis, University of Lethbridge]. ProQuest Dissertations and Theses Global.

LePage, K., & Selk, A. (2016). What Do Patients Want? A Needs Assessment of Vulvodynia Patients Attending a Vulvar Diseases Clinic. *Journal of Sexual Medicine*, *4*(4), e242-e248. https://doi.org/10.1016/j.esxm.2016.06.003

Lightbourne, A., Foley, S., Dempsey, M., & Cronin, M. (2023). Living With Endometriosis: A Reflexive Thematic Analysis Examining Women's Experiences With the Irish Healthcare Services. *Qualitative Health Research*. https://doi.org/https://dx.doi.org/10.1177/10497323231214114

Meana, M., Binik, Y. M., Khalife, S., & Cohen, D. (1999). Psychosocial Correlates of Pain Attributions in Women With Dyspareunia. *Psychosomatics*, *40*(6), 497-502. https://doi.org/10.1016/S0033-3182(99)71188-6

Myrtveit-Stensrud, L., Haugstad, G. K., Rème, S. E., Schaller, S. L., & Groven, K. S. (2023). "It's all my fault": a qualitative study of how heterosexual couples experience living with vulvodynia. *Acta Obstetricia et Gynecologica Scandinavica*, *102*(10), 1378-1389. https://doi.org/10.1111/aogs.14537

Pithavadian, R. (2021). *Understanding Women’s Help-Seeking Experiences for Vaginismus: Implications for Their Constructions of Sense of Self* [Masters thesis, Western Sydney University]. Sydney, Australia.

Savidge, C. J., Slade, P., Stewart, P., & Li, T. C. (1998). Women's Perspectives on their Experiences of Chronic Pelvic Pain and Medical Care. *Journal of Health Psychology*, *3*(1), 103-116. https://doi.org/10.1177/135910539800300108

Shallcross, R., Dickson, J. M., Nunns, D., Taylor, K., & Kiemle, G. (2019). Women’s Experiences of Vulvodynia: An Interpretative Phenomenological Analysis of the Journey Toward Diagnosis. *Archives of Sexual Behavior*, *48*(3), 961-974. https://doi.org/10.1007/s10508-018-1246-z

Twiss, C. E. (2018). *Narratives of Young Women Living with Chronic Pelvic Pain* [Doctoral thesis, New Mexico State University]. ProQuest Dissertations and Theses Global.

Ward, E., & Ogden, J. (2010). Experiencing vaginismus-sufferers' beliefs about causes and effects. *Sexual and Relationship Therapy*, *25*(4), 434-446. https://doi.org/10.1080/14681994.2010.518724

Young, A. L., & Miller, A. D. (2019). "This Girl is on Fire" Sensemaking in an Online Health Community for Vulvodynia. Proceedings of the 2019 CHI Conference on Human Factors in Computing Systems, 1-13. https://doi.org/10.1145/3290605.3300359

# Table S4. Coding tree

| **Themes** | **Categories** | **Codes** |
| --- | --- | --- |
| It’s happening to me because I’m a woman | Women’s lot        Normal      Luck/chance      Menstruation      Not bad enough | How women are  Being a woman  Women’s pain    Normal for women  Part of life    Unlucky rather than ill  Random    Periods  Part of menstrual cycle    Not a legitimate medical issue  Not that serious |
| It’s happening to me in my body | Diagnosed/undiagnosed health condition                    Genital size/structure    I’m dysfunctional    Surgical removal | Undiagnosed pathology  Cyst rupturing  Kidney stone  Endometriosis  Medical condition  Lesions  Something medically wrong  Pelvic joints  Yeast  UTI    Vagina too small     Dysfunctional vulva    Vulva needs to be removed |
| It’s happening to me due to my actions | Activities/behaviours              Self-blame    Penetration      Bad at sex | Sitting/working long hours  Certain activities  Working too hard  Physical activity  Diet  Idleness    Doing it to myself  Doing something wrong    Tampons  Amount of sex  Friction  Sexual inexperience |
| It’s happening to me due to my life | Environment      Stress      Emotions        Childbirth/childbearing      Trauma | Environmental sensitivity  Environmental triggers    Stressful life events  Stressful situations    Pain decreases as fear decreases  Feeling unsafe  Bereavement    Traumatic birth  Miscarriage    Sexual abuse  Traumatic toilet training  Witnessing abuse  Childhood trauma |

# Table S5. Sensitivity analysis

A sensitivity analysis compared the presence of themes across all studies versus those that scored 10/10 on the CASP checklist to assess whether key themes were consistent among the highest-quality studies.

|  | All included studies  (n=30) | High-quality studies  (CASP score 10/10, n = 7) |
| --- | --- | --- |
| Theme 1: It’s happening to me because I’m a woman | 70.00% | 66.67% |
| Theme 2: It’s happening to me in my body | 100.00% | 100.00% |
| Theme 3:  It’s happening to me due to my actions | 76.67% | 100.00% |
| Theme 4: It’s happening to me due to my life | 83.33% | 83.33% |

# Table S6. GRADE-CERQual analysis (Lewin et al., 2018)

The confidence in each review finding was assessed using the GRADE-CERQual approach, which evaluates four components: methodological limitations, coherence, adequacy of data, and relevance. For each theme or finding, we considered the extent to which these factors could lower confidence, and assigned an overall rating of high, moderate, low, or very low confidence. Assessments were conducted independently by two reviewers, with discrepancies resolved through discussion and consensus within the research team.

| Review finding | Methodological limitations | Relevance | Coherence | Adequacy | Overall confidence |
| --- | --- | --- | --- | --- | --- |
| Theme 1:  It’s happening to me because I’m a woman | moderate | minor | minor | none | moderate |
| Theme 2:  It’s happening to  me in my body | moderate | minor | none | none | high |
| Theme 3:  It’s happening to me due to my actions | moderate | minor | minor | none | moderate |
| Theme 4:  It’s happening to  me due to my life | moderate | minor | minor | none | moderate |

Lewin, S., Booth, A., Glenton, C., Munthe-Kaas, H., Rashidian, A., Wainwright, M., Bohren, M. A., Tunçalp, Ö., Colvin, C. J., Garside, R., Carlsen, B., Langlois, E. V., & Noyes, J. (2018). Applying GRADE-CERQual to qualitative evidence synthesis findings: introduction to the series. *Implementation Science*, *13*(1), 2. https://doi.org/10.1186/s13012-017-0688-3
